# Dynamic S-acylation controls CMG2 maturation extracellular matrix regulation and anthrax toxin susceptibility in vivo

**DOI:** 10.1038/s41467-026-75028-2

**Published:** 2026-07-06

**Authors:** Laurence Abrami, Octave Joliot, Sanja Blaskovic, Guillaume Valentin, Covadonga F. Hevia, Vincent Mercier, Béatrice Kunz, Francisco S. Mesquita, F. Gisou van der Goot

**Affiliations:** 1 Global Health Institute, School of Life Sciences, Ecole Polytechnique Federale de Lausanne (EPFL), Lausanne, Switzerland; 2 Center of PhenoGenomics, School of Life Sciences, EPFL, Lausanne, Switzerland; 3 ACCESS-Geneva biomolecular screening platform, University of Geneva, Geneva, Switzerland; 4 Lee Kong Chian School of Medicine, Nanyang Technological University, Singapore, Singapore

**Keywords:** Protein folding, Mechanisms of disease, Post-translational modifications

## Abstract

CMG2/ANTXR2 functions as a Collagen VI receptor required for extracellular matrix homeostasis and as the primary portal for anthrax toxin entry. Mutations in CMG2 cause Hyaline Fibromatosis Syndrome (HFS), a rare and often fatal genetic disorder characterized by excessive extracellular matrix accumulation, yet the molecular mechanisms regulating CMG2 function remain poorly understood. We show that CMG2 is controlled by ordered cycles of S-acylation and deacylation that regulate its folding, trafficking, and signalling competence. S-acylation by ZDHHC7 on two juxtamembrane cysteines protects CMG2 from ER-associated degradation by stabilizing folding intermediates, leading to a ~ 5-fold increase in folded receptors competent for ER exit. In the Golgi, ZDHHC3-dependent acylation of a third cysteine promotes Arf6-mediated CMG2 transport to the plasma membrane, where it exerts its functions. Ligand binding triggers recruitment of the thioesterase APT2, which enables release of CMG2 from the actin cytoskeleton and endocytosis, linking extracellular recognition to intracellular signalling and uptake. Inhibition of APT2 reduces Collagen VI turnover and strongly attenuates anthrax toxin toxicity in a zebrafish model, showing lipidation cycles as regulators of receptor function and potential therapeutic targets.

## Abstract (web summary)

Regulation of membrane receptors governs extracellular matrix homeostasis and host-pathogen interactions. Here, authors show that reversible S-acylation controls CMG2 maturation, trafficking, and signaling, and that inhibiting deacylation protects against anthrax toxin in vivo.

## Introduction

Capillary Morphogenesis Gene 2 (CMG2/ANTXR2) is a ubiquitously expressed type I membrane protein that plays a central role in extracellular matrix (ECM) homeostasis^1–4^. Loss-of-function mutations in CMG2 cause Hyaline Fibromatosis Syndrome (HFS), a severe genetic disease characterized by excessive accumulation of collagen type VI (COL6) and other ECM components^4–7^. CMG2 also serves as the major mammalian receptor for the anthrax toxins^8^. Despite its central roles in both physiology and infection, how CMG2 biogenesis and signalling are regulated at the molecular level remains poorly understood.

We previously reported that CMG2, and its paralog TEM8 (ANTXR1), which is also an anthrax toxin receptor^9^ and associated with genetic disease^10^, GAPO syndrome, undergo S-acylation and that this is required for productive anthrax toxin internalization^11^. S-acylation refers to the covalent attachment of medium-chain fatty acids, typically palmitate, to cytosolic cysteine residues via thioester bonds^12–14^. This process is catalysed by the transmembrane ZDHHC family of acyltransferases and reversed by cytosolic acyl-protein thioesterases such as APT1/LYPLA1, APT2/LYPLA2, and ABHD17 family members^13–15^. This dynamic modification can regulate protein conformation, subcellular trafficking, protein-protein interactions, turnover, and function^16^. While S-acylation delays anthrax toxin endocytosis^11^, the molecular consequences of CMG2 acylation, its dynamics, and the enzymes involved have remained unknown.

**Fig. 1:**
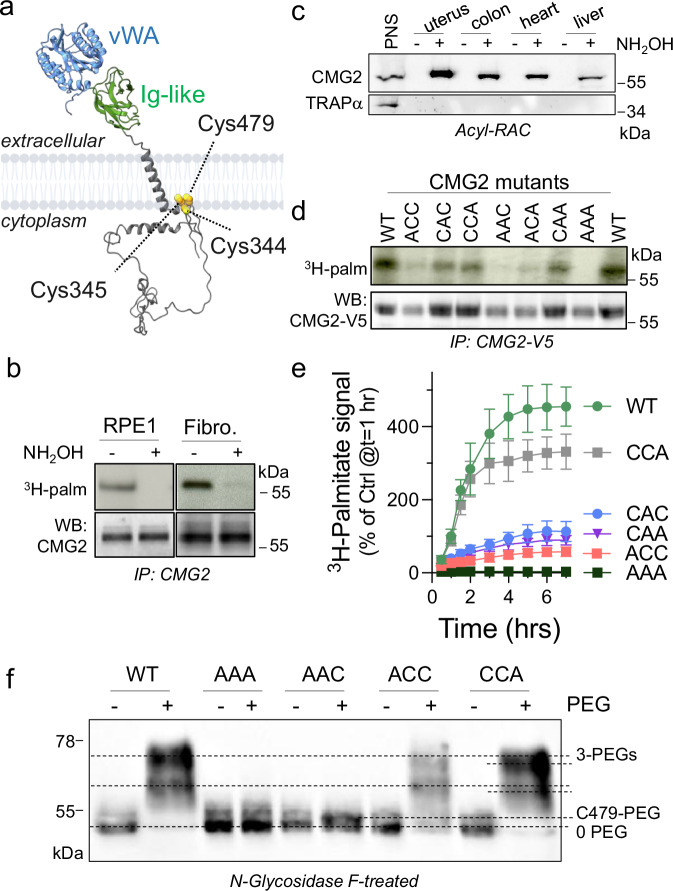
CMG2 is S-acylated on Cys-344, Cys-345 and Cys-479. **a** Ribbon representation of CMG2 domain organization. The extracellular region, composed of a von Willebrand A (vWA) and an Immunoglobulin-like (Ig-like) is based on the 2.6 Å cryo-EM structure (EMD-54238)^18^. The transmembrane helix and cytosolic domain were predicted using AlphaFold3. The position of the TMD was predicted using PPM3.0 Web Server. The cytosolic helix was manually adjusted to allow space for the membrane. The three cytosolic cysteines present in the tail of CMG2 isoform 4 are indicated. **b** RPE1 cells or primary human fibroblasts were metabolically labeled for 2 h at 37 °C with [³H]-palmitic acid. CMG2 was immunoprecipitated and samples were treated or not with hydroxylamine ( + NH₂OH) to cleave thioester-linked acyl chains. Proteins were separated by SDS–PAGE and analyzed by autoradiography (³H-palm) and by immunoblotting against CMG2. **c** CMG2 in mouse organs was analyzed using Acyl-RAC. Equal volumes of input (Inp.) and Acyl-RAC eluates ( + NH₂OH) were separated by 4–20% SDS–PAGE. Immunoblotting was performed with anti-mouse CMG2 (rat monoclonal 8F7) or anti-TRAPα as a control. A Post Nuclear Supernatant (PNS) from RPE1 cells was added as positive control for the CMG2 and TRAPα bands. **d** HeLa cells were transfected for 24 h with WT and mutants CMG2-V5. AAA corresponds to the three cysteines mutated to alanine, CCC = WT to all cysteine, other mutants refer to different cysteine mutants. Cells were labeled for 2 h with [³H]-palmitate at 37 °C and CMG2-V5 was immunoprecipitated. Autoradiography and anti-V5 immunoblotting were used to detect S-acylated and total CMG2, respectively. **e** HeLa cells expressing WT or cysteine mutants were labeled for different times with [³H]-palmitate at 37 °C. CMG2-V5 was immunoprecipitated and analyzed as in (**d**). ³H-palmitate incorporation at 1 h was set to 100%, and each timepoint expressed relative to this value (*n* = 3 corresponding to 3 full kinetics with all time points, mean ± SD). **f** Hela cells were transfected 24 h with CMG2-V5 WT, AAA, AAC, ACC or CCA, and treated for acyl-PEG exchange. All lysates were then subjected for N-glycosidase F for 16 h and followed by SDS-PAGE and WB for CMG2-V5, mass-tagged proteins ( + PEG), and control (-PEG), after hydroxylamine treatment.

CMG2 is synthesized with a signal sequence leading to its co-translational insertion into the membrane of the endoplasmic reticulum (ER)^17^. There, it undergoes folding, with the assistance of glycan-binding chaperones^17^. However, the CMG2 ectodomain^18^ (Fig. 1a) folds inefficiently, which reduces tolerance to missense mutations^19^. A variety of HFS missense mutations impair CMG2 folding, resulting in its degradation via the ER-associated degradation (ERAD) and protein-loss dependent loss-of-function^3,19,20^. How CMG2 folding efficiency is regulated during biogenesis, however, remains poorly understood.

Once folded, CMG2 exits the ER and transits through the Golgi to reach the plasma membrane^19^. At the cell surface, ligand-free CMG2 associates with the cytoskeleton by interacting with a Talin-Vinculin-Actin (TVA) containing complex^21^. This TVA interaction is released when a ligand, such anthrax toxin or Collagen VI^4^ binds to the ectodomain of CMG2, so that its cytoplasmic domain can then engage proteins recruited for CMG2 signalling and endocytosis such as RhoA, β-arrestin, and Cbl^11,21,22^. This transition is essential for both toxin uptake and CMG2-mediated COL6 internalization^21^, yet whether and how S-acylation participates in this signalling switch has not been addressed.

Here we show that CMG2 function is controlled by temporally ordered S-acylation-deacylation cycles that coordinate receptor maturation, trafficking, and signalling. We show distinct acyltransferases and thioesterases acting at successive stages of the CMG2 life cycle, linking lipid modification to ER quality control, secretory transport, and ligand-induced receptor activation. Our findings reveal that reversible lipidation modulates CMG2 abundance, controls its ability to undergo ligand-induced conformational switching, and governs downstream endocytic signalling. Consistent with this regulatory role, genetic or pharmacological modulation of deacylation alters extracellular matrix turnover and influences susceptibility to anthrax toxin in vivo. These results show dynamic S-acylation as a central regulator of CMG2 biology and highlight deacylation enzymes as potential therapeutic targets in both HFS and anthrax intoxication.

## Results

### CMG2 undergoes sequential S-acylation of its three cytosolic cysteines

CMG2 and its paralogue TEM8 have been reported to undergo S-acylation^11^ (Fig. 1a). We confirmed this finding in cultured cells using [³H]-palmitate metabolic labelling, and in mouse tissues using the Acyl-RAC assay, which captures S-acylated proteins in cell extracts (Fig. 1bc).

CMG2 isoform 4 contains three cytosolic cysteines (Cys344, Cys345, and Cys479; Fig. 1a). We generated a panel of single, double, and triple cysteine-to-alanine mutants, with the wild-type (WT) corresponding to CCC and the triple C344A-C345A-C479A mutant denoted as AAA. The ACC, AAC and AAA mutants were expressed at a slightly reduced level (Supplementary Fig. 1a). Loss of all three cysteines abolished [³H]-palmitate incorporation (Fig. 1d, Supplementary Fig. 1b). Among the double mutants, the AAC variant (where Cys344 and Cys345 are mutated) showed the strongest reduction, only partially explained by its lower expression level, suggesting that S-acylation of Cys344 and 345 might facilitate modification of the most distal cysteine. Kinetic labelling experiments showed that efficient S-acylation of CMG2 requires the sequential modification of cysteines from the transmembrane domain to the C-terminal cytosolic tail (Fig. 1e).

We next assessed site occupancy using PEGylation, in which hydroxylamine cleaves the thioester bond, enabling labelling of the liberated cysteine with PEG-maleimide of 5 kDa. Because CMG2 is glycosylated on two asparagine residues, it migrates as a smear in SDS-PAGE^17^, complicating interpretation of PEGylation-induced mobility shifts. We therefore treated all samples with N-glycosidase F to remove the N-linked sugars prior to SDS-PAGE. The AAA mutant did not show any band shift upon PEGylation, indicating the specificity of the assay (Fig. 1f). For the AAC mutant, a slightly shifted band could be observed, in addition to the unshifted band, indicating that some CMG2 molecules had undergone acylation on Cys479 (Fig. 1f). For the CCA mutant, the initial (unshifted) band essentially disappeared, indicating that the entire CCA CMG2 population underwent acylation. Two shifted bands were observed, the upper, and major, band thus corresponding to dual acylated CCA (Fig. 1f). The ACC mutant showed a similar pattern, but at greatly reduced intensity, suggesting that acylation of the cysteine can occur, but only does so on a limited number of molecules withing the CMG2 ACC population. Finally, the WT protein also showed a pattern similar to CCA, but with an upper band migrating slightly higher that the CCA upper band, consistent with S-acylation of all three cysteines (Fig. 1f).

Altogether, this analysis indicates that CMG2 undergoes S-acylation sequentially, first on the two juxtamembranous cysteines, which facilitates the modification of the most distant cysteine. At steady state, the entire WT CMG2 population is S-acylated, mostly on all three cysteines.

### CMG2 undergoes sequential S-acylation by ZDHHC7 and ZDHHC3

**Fig. 2:**
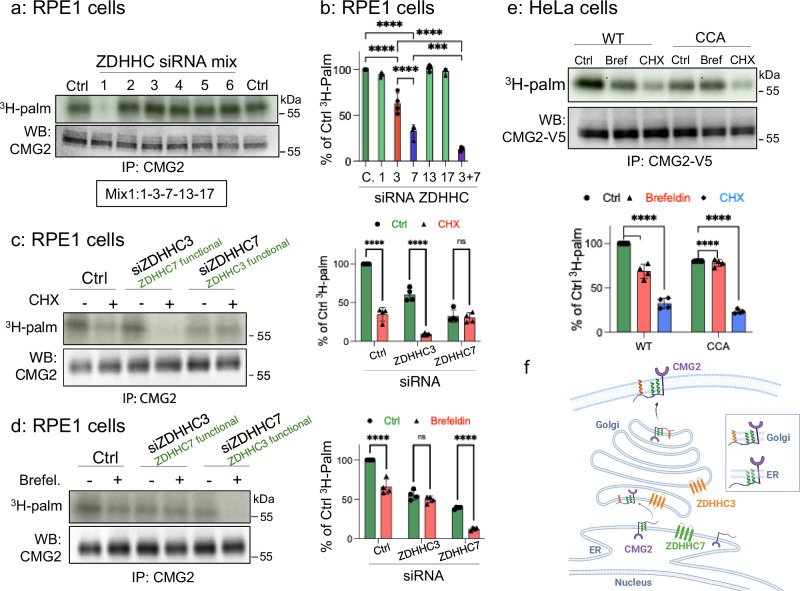
CMG2 S-acylation is mediated by ZDHHC3 and ZDHHC7. RPE1 cells were silenced with pooled/mixed (**a**, the composition of the pools is provided in the Methods section) or individual (**b**) siRNAs targeting human ZDHHC enzymes, or non-targeting control siRNA. Cells were metabolically labeled for 2 h at 37 °C with [³H]-palmitic acid, CMG2 was immunoprecipitated, and samples were analyzed by SDS–PAGE followed by autoradiography and anti-CMG2 immunoblotting. [³H]-palmitate incorporation was quantified by Typhoon imaging and normalized to control = 100% (**a**: *n* = 3; b: *n* = 4; mean ± SD). *p* values were calculated by two-way ANOVA, ****: approximate *p* < 0.0001; ***: *p* < 0.001. **c**, **d** RPE1 cells were silenced with siZDHHC3, siZDHHC7, or control siRNA, then pre-treated or not with cycloheximide (CHX) or Brefeldin A (BrefA) for 1 h and maintained under the same conditions during metabolic labeling. Cells were labeled for 2 h at 37 °C with [³H]-palmitate, CMG2 was immunoprecipitated with anti-CMG2, and samples were analyzed by SDS–PAGE, autoradiography (³H-palm), and immunoblotting with anti-CMG2. Radiolabel incorporation was quantified by Typhoon imaging and normalized to control siRNA/untreated = 100% (*n* = 4; mean ± SD). *p* values were calculated by two-way ANOVA, ****: approximate *p* < 0.0001. **e** HeLa cells were transfected with WT or CCA CMG2-V5, and then pre-treated as in **c**, **d** with cycloheximide or Brefeldin A, and treated with [³H]-palmitate. CMG2 was immunoprecipitated using an anti-V5 antibodies. Quantifications were performed as in **c**, **d** (*n* = 4; mean ± SD). *p* values were calculated by two-way ANOVA, ****: approximate *p* < 0.0001. **f** Sequential CMG2 S-acylation occurs first on Cys344/345 by ZDHHC7 in the ER, followed by Cys479 modification by ZDHHC3 in the Golgi. Created in BioRender. Abrami, L. (2026) https://BioRender.com/sx07sq9.

To identify the acyltransferases responsible for CMG2 modification, we performed a siRNA screen targeting all 23 human ZDHHC enzymes, grouped into six pools. Only pool M1 led to a reduction in CMG2 S-acylation (Fig. 2a, Supplementary Fig. 2a). Subsequent individual knockdowns within this pool revealed that ZDHHC7 and ZDHHC3 are responsible for mediating CMG2 S-acylation (Fig. 2b, Supplementary Fig. 2b,c). Recomplementation of ZDHHC3 or 7 silenced cells with the corresponding acyltransferase led to recovery of the S-acylation level (Supplementary Fig. 2d). These two enzymes also S-acylate the 4 cysteines present in TEM8^11^ (Supplementary Fig. 2e,f).

We next investigated whether the two enzymes act on different cysteines, and possibly in different intracellular compartments. ZDHHC7 localizes to the early secretory pathway (ER and Golgi) and ZDHHC3 to the Golgi^23^. To investigate whether S-acylation occurs in the ER when CMG2 is newly synthesized, we inhibited protein synthesis with cycloheximide. We monitored ZDHHC7-mediated S-acylation by silencing ZDHHC3, and conversely ZDHHC3-mediated S-acylation by silencing ZDHHC7. ZDHHC7-mediated S-acylation was essentially abolished by cycloheximide (CHX) (Fig. 2c), indicating that ZDHHC7 modifies primarily newly synthesised proteins. In contrast, ZDHHC3-mediated S-acylation was unaffected by cycloheximide (Fig. 2c), consistent with ZDHHC3 acting in the Golgi on CMG2 molecules synthesized prior to drug addition. Next, we tested the effect of disrupting the Golgi with brefeldin A, and observed the opposite pattern: ZDHHC7-mediated S-acylation was unaffected by brefeldin A, whereas ZDHHC3-mediated S-acylation was essentially abolished (Fig. 2d). Finally, we tested whether the CCA (i.e. C479A only) mutation would phenocopy ZDHHC3 silencing. Cycloheximide treatment strongly reduced CCA S-acylation while brefeldin A treatment had no effect (Fig. 2e). Thus, CMG2 undergoes sequential S-acylation, first in the ER on Cys344 and Cys345 by ZDHHC7, and subsequently on Cys479 by ZDHHC3 in the Golgi (Fig. 2f).

### S-acylation-deacylation controls maturation of CMG2 in the ER

**Fig. 3:**
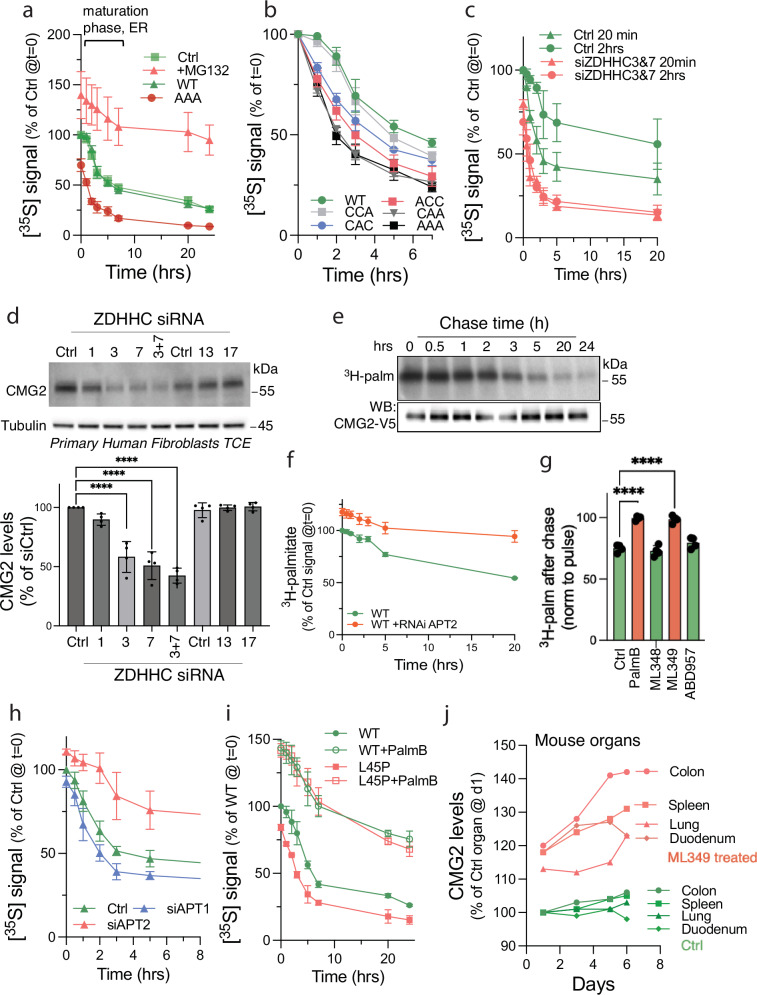
ZDHHC3 and ZDHHC7 control CMG2 biogenesis. HeLa cells expressing WT or mutant CMG2 (**a, b**), or RPE1 cells silenced with individual ZDHHC siRNAs (**c**), were pulse-labeled for 20 min with [³⁵S]-Met ± MG132 (**a**) and chased for the indicated times. CMG2 was immunoprecipitated and analyzed by SDS–PAGE, autoradiography and anti-CMG2 immunoblotting. Radiolabel incorporation was quantified (Typhoon) and normalized to either untreated WT/control = 100% for **a** and **c**, or for **b**, each condition at t = 0 (for all *n* = 3, mean ± SD). In **b**, AAA, ACC, CAC, and CAA are statistically different from WT at all time points of the chase (*p* < 0.05). CCA is statistically different from AAA at all time points, and statistically different from WT at and beyond the 5 h time point. **d** Primary fibroblasts silenced for 72 h with the indicated siRNAs were analyzed by immunoblot of total cell extracts (40 µg) for endogenous CMG2 ± Tubulin loading control (*n* = 4, mean ± SEM). *p* values were calculated by two-way ANOVA, ****: approximate *p* < 0.0001. **e** HeLa cells expressing CMG2-V5 were labeled 2 h with [³H]-palmitate and chased as indicated. CMG2 was immunoprecipitated and analyzed by autoradiography and immunoblot. **f** Hela cells were silenced or not with siAPT2 and then treated as in **e**. [³H]-palmitate signal after chase was normalized to pulse timepoint (*n* = 3, mean ± SD). **g** RPE1 cells treated with Palmostatin B, ML348, ML349 or ABD957, then labelled with [³H]-palmitate as in **e**, and chased for 5 h. [³H]-palmitate signal after chase was normalized to pulse timepoint (*n* = 4, mean ± SD). *p* values were calculated by two-way ANOVA, ****: approximate *p* < 0.0001. Effects of deacylation inhibition on folding. RPE1 cells silenced for APT1/2 (**h**) or HeLa expressing WT or L45P CMG2 ± Palmostatin B (**i**) were pulse-labeled 20 min with [³⁵S]-Met and chased. CMG2 was immunoprecipitated, quantified, and expressed relative to control = 100% (*n* = 3, mean ± SD). **j** In vivo stabilization: mice were treated i.p. with ML349 (40 mg/kg; days 0,2,4,6,8), organs collected at indicated time points, and endogenous CMG2 measured by immunoblot of tissue lysates (40 µg). The levels of CMG2 signals were quantified by densitometry.

Because CMG2 is S-acylated during or shortly after synthesis, we asked whether this modification influences its biogenesis and thus the amount of fully folded protein that exits the ER. We performed [³⁵S]-Cys/Met pulse-chase labelling with a short 20 min pulse to focus on newly synthesized molecules. CMG2 decayed with a t_₁/₂_ of ~6.5 h, both for endogenous (Ctrl) and ectopically expressed (WT) protein (Fig. 3a, Supplementary Fig. 3a,b). Inhibition of the proteasome with MG132 increased the signal at t = 0 and markedly delayed and reduced degradation, indicating that in untreated cells, a major fraction of newly synthesized CMG2 is destroyed during the first 5–6h (maturation phase, Fig. 3a) by ERAD, reminiscent of the behaviour of wild type cystic fibrosis transmembrane regulator (CFTR)^24^ and its most common ΔF508 disease mutant^25,26^. When we performed a similar experiment on the AAA triple mutant, degradation of the newly synthesized protein was significantly accelerated (t₁/₂ ≈ 1.2 h) and more pronounced, with degradation already significant during the pulse period (Fig. 3a, Supplementary Fig. 3a, b). Silencing ZDHHC7 and 3 completely recapitulated the effect of mutating the three cysteines (Supplementary Fig. 3a, b). Accelerated degradation following the metabolic pulse was also observed when mutating only Cys344 or Cys345 (Fig. 3b, normalised to t = 0 to facilitate kinetic comparison), consistent with these residues being S-acylated in the ER and protecting folding intermediates from ERAD.

The difference in the [³⁵S]-Cys/Met signal at t = 0 for WT and AAA CMG2 indicates that some WT molecules undergo S-acylation during the 20 min pulse. However S-acylation of the pool of newly synthesized CMG2 molecules may take longer than 20 min. To test this, we compared the effects of 20 min and 2 h [³⁵S]-Cys/Met labeling durations. Extending the pulse period resulted in an apparent reduction and delay in the degradation of the pool of newly synthesized WT CMG2 (Fig. 3c). The effect of the pulse length was lost when silencing ZDHHC7 and ZDHHC3 (Fig. 3c), confirming that reduced degradation observed with longer pulse duration reflects enrichment of stabilised molecules rather than altered degradation kinetics. We have made similar observations for two other S-transmembrane proteins undergoing S-acylation in the ER, calnexin and LRP6 and for both decay kinetics were strongly influenced by the [³⁵S]-Cys/Met labeling time^27,28^. Supporting the importance of S-acylation during biogenesis, silencing ZDHHC7 and ZDHHC3 in primary human fibroblasts caused a marked reduction in total CMG2 protein levels (Fig. 3d).

### Deacylation by APT2 in the ER targets CMG2 to premature ERAD

Since S-acylation is reversible, we next examined whether during biogenesis in the ER, CMG2 also undergoes deacylation. To address this, we first assessed whether CMG2 can be deacylated at all. [³H]-palmitate pulse-chase analysis, using a 2 h pulse, showed a slow and progressive loss of radiolabel from CMG2 (Fig. 3e,f). Loss of [³H]-palmitate signal can occur by deacylation as well as by protein degradation. To determine the contribution of deacylation, we tested the effects of acyl-protein thioesterase inhibitors. The pan-thioesterase inhibitor Palmostatin B and the APT2-specific inhibitor ML349, but not the APT1 inhibitor ML348 or the ABHD17 inhibitor ABD957, prevented the loss of [³H]-palmitate during the chase period (Supplementary Fig. 3c, Fig. 3g). Kinetic measurements showed that APT2 silencing indeed substantially delayed and reduced the loss of [³H]-palmitate from CMG2 (Fig. 3f, Supplementary Fig. 3d).

To more specifically determine whether APT2, which is a cytosolically expressed enzyme, can act on CMG2 during biogenesis, we again performed [³⁵S]-Cys/Met pulse-chase experiments. Knockdown of APT2 protected newly synthesized CMG2 from ERAD, with stabilization evident already during the pulse (Fig. 3f). The effect was even more striking when cells were treated with Palmostatin B, for reasons that remain to be clarified (Supplementary Fig. 3e), which was here used for practical reasons rather than the APT2 inhibitor ML349. Importantly, Palmostatin B did not affect the decay kinetics of the AAA mutant, indicating that the observed effects are related to S-acylation of WT CMG2 (Supplementary Fig. 3e).

These results show that during the folding of a newly synthesised population of CMG2, APT2 can remove the protective S-acylations, thereby promoting the premature targeting of these molecules to ERAD. Quantitatively, comparison of CMG2 levels 5 h after the pulse reveal an approximately 4 to 6-fold difference between ZDHHC7/3 silencing, APT2 silencing and PalmB (*p* = 0.001, Fig. 3c,h, Extended Fig. 3f), highlighting the major contribution of S-acylation to CMG2 folding efficiency.

This mechanism has implications for Hyaline Fibromatosis Syndrome (HFS) missense mutations that affect folding^3,19,20^. We previously showed that several of these mutations, which map to the extracellular von Willebrand A domain, fold properly when produced in *E. coli*^19^, indicating that in human cells it is the slower folding kinetics—rather than an intrinsic inability to fold—that triggers ERAD recognition. Our current findings suggest that inhibiting deacylation, to protect folding intermediates from ERAD targeting, should lead to an increase in the amount of successfully folded receptors. We tested this hypothesis on the L45P HFS mutation^19^. Indeed, Palmostatin B prevented the premature degradation of CMG2 L45P and led to an increase of CMG2 levels, reaching similar levels as observed for WT CMG2 (Fig. 3i).

Because L45P is retained in the ER through recognition by the ER quality control machinery, these observations suggest that CMG2 deacylation can occur at the cytosolic surface of the ER. To test whether APT2 can access CMG2 in this compartment, we immunoprecipitated the ER-retained L45P mutant and assessed its interaction with APT2. We observed robust co-immunoprecipitation between L45P CMG2 and APT2 (Extended Fig. 3g). We extended this analysis to three additional HFS-associated mutations that cause ER retention of CMG2 (G105D, I189T, and L349R), all of which also interacted with APT2 (Extended Fig. 3g).

As a further proof of concept for the therapeutic potential of APT2 inhibition to increase the CMG2 protein levels, we treated mice by intraperitoneal injection with the APT2 inhibitor ML349 for several days, sacrificed the animals at defined time points, and collected multiple organs for analysis. ML349 treatment led to a time-dependent increase in CMG2 abundance across all tissues tested, as most apparent in the colon, (Fig. 3j, Supplementary Fig. 3h), without significant changes in mRNA levels (Supplementary Fig. 3i). Altogether, the present findings show that newly synthesized CMG2 is robustly protected from ERAD through the S-acylation of juxtamembrane cysteines and indicate that inhibition of APT2 could be a potential strategy for improving CMG2 expression in contexts where receptor folding is impaired.

### CMG2 trafficking from Golgi to the plasma membrane is ZDHHC3-dependent and Arf6-mediated

**Fig. 4:**
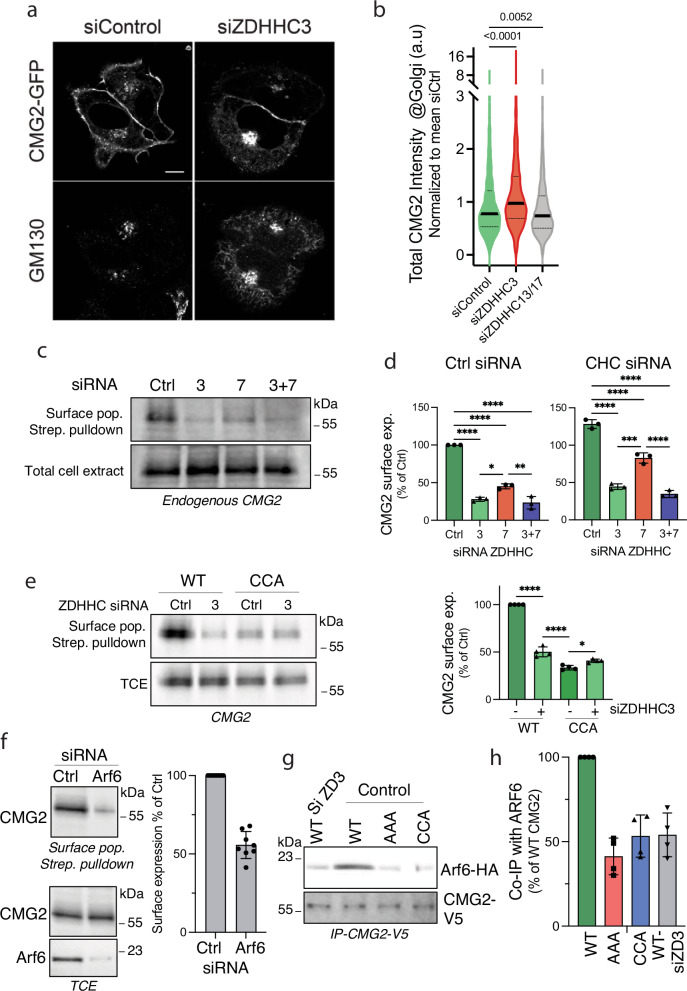
ZDHHC3-dependent, Arf6-mediated trafficking of CMG2 to the plasma membrane. **a** Immunofluorescence microscopy of CMG2-GFP–expressing HeLa cells treated for 72 h with control or ZDHHC3 siRNA. GM130 marks the Golgi. Scale bar, 10 µm. **b** High-throughput automated immunofluorescence quantification of CMG2-GFP localization in HeLa cells transfected with control siRNA, siZDHHC3, or siZDHHC13/17 (72 h) and expressing CMG2-GFP for 24 h. Data represent integrative intensity values at the Golgi, defined by GM130-positive regions, and normalized to siCtrl. Bar graphs show mean values ± s.e.m. from one of two independent experiments with equivalent results. *n* = 2548 (siCtrl), 2540 (siZDHHC3), and 2756 (siZDHHC13/17) cells analyzed. *p* values calculated by one-way ANOVA followed by Dunnett’s multiple-comparisons test against siCtrl. The *p* value for the siCtrl vs. siZDHHC3 comparison is approximate. **c**.RPE1 cells were silenced for 3 days with siZDHHC3, siZDHHC7, both, or control RNAi. Plasma membrane proteins were biotinylated, total lysates collected, and streptavidin pull-downs probed for endogenous CMG2 by western blot (*n* = 3, mean ± SE). **d** Clathrin Heavy Chain was silenced in order to inhibit anthrax PA induced endocytosis of CMG2. Quantification of biotinylated CMG2 surface levels under conditions in (**c**) (*n* = 3, mean ± SE). *p* values were calculated using one-way ANOVA. ****p < 0.0001, ***p < 0.001, **p < 0.01, *p > 0.05. **e** HeLa cells were silenced for 3 days with siZDHHC3 or control RNAi, then transfected for 24 h with CMG2-V5 WT or CCA. Surface proteins were biotinylated and streptavidin precipitates probed with anti-V5 (*n* = 4, mean ± SE) *p* values were calculated using one-way ANOVA. ****p < 0.0001, *p > 0.05. **f** RPE1 cells were silenced for 3 days with siARF6 or control RNAi, plasma membrane proteins biotinylated, precipitated with streptavidin, and membranes blotted for endogenous CMG2 and ARF6 (*n* = 8, mean ± SE). **g** RPE1 cells were silenced for 3 d with siZDHHC3 or control RNAi, transfected 24 h with CMG2-V5 WT, CCA, or AAA together with ARF6-HA, immunoprecipitated using V5 beads, and blotted for CMG2-V5 and ARF6-HA (*n* = 4, mean ± SE). **h** Quantification of co-immunoprecipitated ARF6 with different CMG2 constructs or with cells silenced with siZDHHC3 (siZD3) (*n* = 4, mean ± SE).

We next investigated the functional consequences of ZDHHC3-mediated S-acylation of CMG2 at its distal cytosolic cysteine. In control cells, CMG2 was predominantly detected at the plasma membrane, whereas ZDHHC3 knockdown caused a marked accumulation of CMG2 in the Golgi (Fig. 4a), a result confirmed by high-content image analysis (Fig. 4b). Consistently, surface biotinylation revealed a strong reduction in CMG2 plasma membrane levels upon ZDHHC3 silencing (Fig. 4c,d). To exclude that the lower plasma membrane CMG2 was due to enhanced CMG2 endocytosis in ZDHHC3 silenced cells as an alternative explanation, we repeated the surface biotinylation assay upon silencing of clathrin heavy chain (CHC)-silenced cells, which prevents CMG2 endocytosis^22^. Under these conditions, the effect of ZDHHC3 depletion on CMG2 surface abundance was even more striking (Fig. 4d). Thus, the decrease in surface CMG2 upon ZDHHC3 silencing is due to a defect in anterograde trafficking to the plasma membrane rather than increased internalization. This might not be specific for CMG2, possibly also occurring for other ZDHHC3 targets.

Because ZDHHC3 S-acylates Cys479, we tested whether the CCA mutant would match the behaviour of WT CMG2 in ZDHHC3 silenced cells. This mutant exhibited reduced plasma membrane localization and was insensitive to ZDHHC3 knockdown (Fig. 4e), confirming that S-acylation of Cys479 promotes CMG2 surface delivery. Since Golgi-to-plasma membrane transport of S-acylated proteins has been proposed to depend on Arf6^29^, we tested its potential role. Silencing Arf6 led to decreased CMG2 surface levels (Fig. 4f), and the interaction between CMG2 and Arf6 was significantly reduced when Cys479 was mutated, or ZDHHC3 was depleted (Fig. 4g,h).

Together, these findings show that ZDHHC3-mediated S-acylation of Cys479 allows CMG2 to engage Arf6 and traffic efficiently from the Golgi to the plasma membrane.

### CMG2 S-acylation is required for ligand-induced cytoskeletal disengagement and signalling

**Fig. 5:**
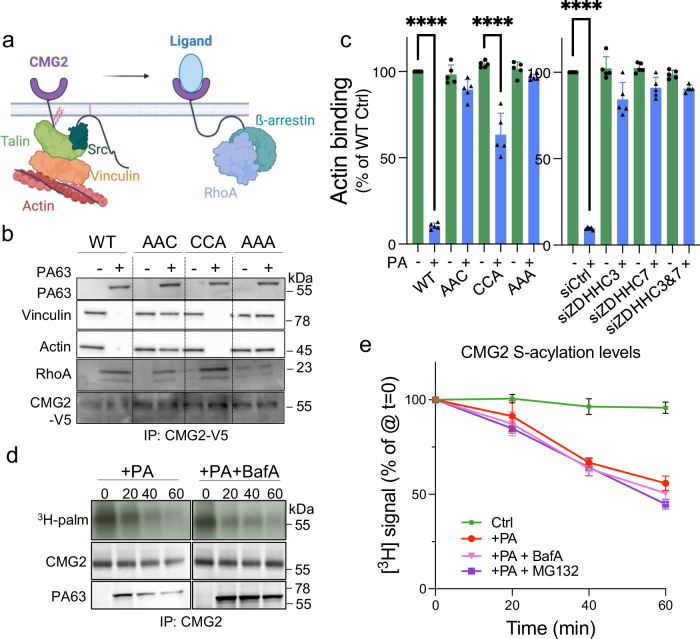
S-acylation controls the CMG2 TVA to RhoA signalling switch and ligand-induced deacylation. **a** Schematic of CMG2 signalling at the plasma membrane. In the resting state, CMG2 associates with Talin-Vinculin-Actin (TVA). Ligand binding (Protective Antigen PA) disrupts TVA and promotes recruitment of RhoA and β-arrestin, enabling endocytosis and downstream cytoskeletal signalling. Created in BioRender. Abrami, L. (2026) https://BioRender.com/9nbx8cq**b** HeLa cells were transfected for 48 h with CMG2-V5 WT or cysteine mutants, then incubated ± PA (500 µg/ml; 1 h at 4 °C followed by 30 min at 37 °C). CMG2 was immunoprecipitated in PBS/1% Triton X-100 with anti-V5 for 4 h at 4 °C and analyzed by SDS-PAGE and immunoblotting using anti-PA, anti-Vinculin, anti-Actin, anti-RhoA, and anti-V5. **c** Quantification of CMG2 association with Actin and APT2 from (**b**) for WT and cysteine mutants, and upon silencing of siZDHHC3+siZDHHC7 (*n* = 5, mean ± SD). *p* values were calculated by one-way ANOVA, ****: approximate *p* < 0.0001. **d** RPE1 cells were labelled 2 h with [³H]-palmitate at 37 °C, washed into chase medium for 2.5 h, then treated ± PA (500 µg/ml) ± Bafilomycin A (BafA, 100 nM) or ± MG132 (10 µM). CMG2 was immunoprecipitated with anti-CMG2, analyzed by SDS-PAGE and autoradiography (³H-palm) and immunoblotting with anti-CMG2 and anti-PA. **e** Radiolabel incorporation was quantified by Typhoon; [³H]-palmitate at 2.5 h (pre-chase) was set to 100%, and timepoints were expressed relative to this (*n* = 3, mean ± SD).

We next investigated whether the S-acylation status of CMG2 influences its functions, first focusing on ligand-induced signalling at the plasma membrane^21^. In the absence of ligand, surface CMG2 interacts with Talin and thereby Vinculin and Actin (TVA), anchoring it to the actin cytoskeleton^21^. Ligand binding however triggers a switch from TVA binding to recruitment of the actin regulators RhoA and mDia, as well as with proteins required for signalling and endocytosis such as the scaffold protein β-arrestin or the kinase src, which then phosphorylates CMG2^11,21,22,30,31^, (Fig. 5a). This switch is readily visible when analysing CMG2 immunoprecipitates under conditions where cells are treated or not with the receptor binding component of the anthrax toxin, the protective antigen (PA), either in its 83 or 63 kDa form at 4 °C followed by a 30 min incubation at 37 °C: in control cells, vinculin and actin co-IP with CMG2, while in toxin treated cells, the receptor is associated with RhoA (Fig. 5b). The TVA release is not observed when cells are kept at 4 °C^21^. The AAA CMG2 mutant failed to release TVA components in response to PA binding, and consistently did not recruit RhoA (Fig. 5b,c). Note that the presence of PA in the CMG2 IP indicates that transient transfection of AAA leads to sufficient expression of the mutant at the cell surface to monitor the binding. The CCA mutant exhibited a partial phenotype, with experiment-to-experiment variability, while the AAC mutant retained significant TVA binding and consistently showed limited RhoA recruitment (Fig. 5b,c). We believe that this reflects a heterogenous population, with some AAC CMG2 molecules binding TVA and others RhoA, rather than a simultaneous binding to TVA and RhoA, which our previous data indicates to be incompatible^21^. Similarly, silencing ZDHHC7 and ZDHHC3 prevented PA-induced TVA release, despite PA binding, and RhoA recruitment (Fig. 5c). This was also the case for the CMG2 ligand present in serum (so far uncharacterized)^21^, as well as for Collagen VI (Supplementary Fig. 4a,b).

These findings show that CMG2 must be S-acylated for it to transduce ligand binding information into cytosolic signalling, likely by constraining the cytoplasmic tail so that the ligand-induced conformational change in the ectodomain can be propagated across the membrane, enabling release of the TVA complex.

### Ligand-induced CMG2 deacylation

We have previously shown that non-acylated anthrax toxin receptors lead to premature unproductive toxin uptake, i.e. where monomeric PA is internalized but the enzymatic subunits of the toxin remaining outside of the cell^11^. This greater endocytic ability of non-acylated CMG2 led us to ask whether ligand binding could induce deacylation of WT CMG2. To test this, cells were labelled with [³H]-palmitate for 2 h, chased to allow CMG2 to reach the plasma membrane, and then stimulated with toxin. PA induced a time-dependent loss of [³H]-palmitate (Fig. 5d). This decay could not be prevented by treating cells with the vacuolar ATPase inhibitor Bafilomycin A, excluding the possibility that the observed loss of [³H]-palmitate occurred by lysosomal degradation of CMG2 (Fig. 5d). Similarly, the proteasome inhibitor MG132 had no effect (Fig. 5d,e, Extended Fig. 4c). Thus, extracellular ligand binding triggers intracellular CMG2 deacylation, a regulatory step that we had previously hypothesized but could not directly demonstrate without the mechanistic insight into CMG2 S-acylation provided by the present study.

### Ligand binding triggers recruitment of membrane-bound APT2

**Fig. 6:**
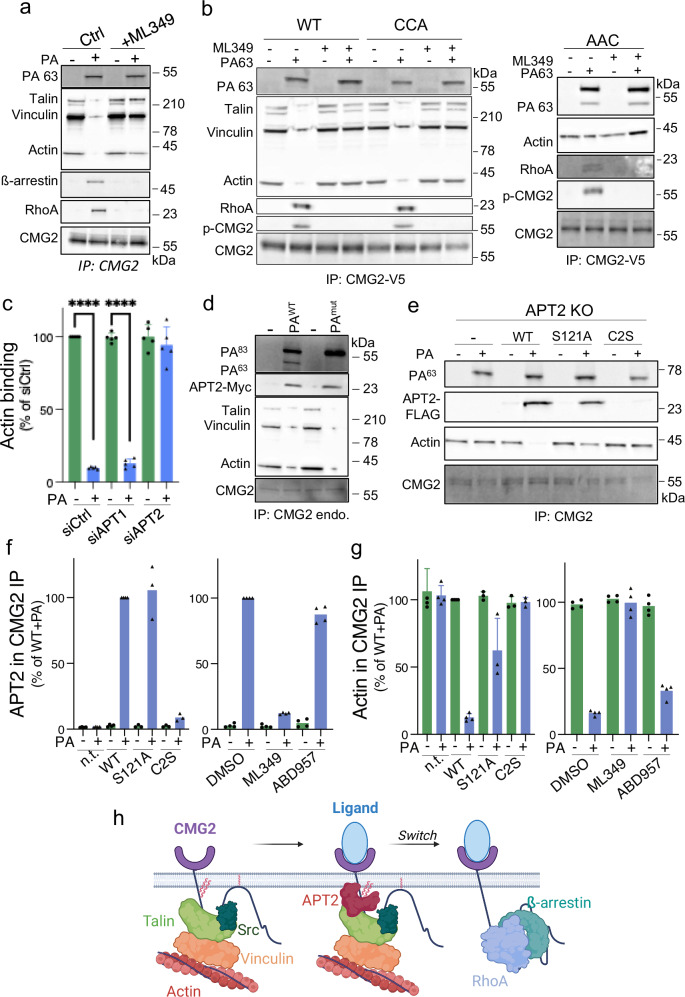
Ligand binding triggers APT2 recruitment and CMG2 deacylation. **a** RPE1 cells were serum-starved, pre-incubated ± ML349 (APT2 inhibitor), and stimulated ± PA/LF at 4 °C followed by 37 °C; CMG2 was immunoprecipitated and analyzed by SDS–PAGE and immunoblotting for PA (Protective Antigen), Talin, Vinculin, Actin, β-arrestin, RHOA and CMG2. **b** HeLa cells were transfected 48 h with WT, CCA or AAC CMG2-V5, serum-starved, pre-treated ± ML349 and stimulated ± PA/LF; CMG2-V5 immunoprecipitates were analyzed as in (**a**) and additionally probed for phosphotyrosine. **c** RPE1 cells were silenced 3 days with siAPT1 or siAPT2, serum-starved and stimulated ± PA/LF; CMG2 immunoprecipitates were analyzed for PA, Actin and CMG2, with Actin co-IP in control −PA normalized to 100% (*n* = 5, mean ± SD). *p* values were calculated by one-way ANOVA, ****: approximate *p* < 0.0001. **d** RPE1 cells expressing APT2-Myc WT were stimulated with WT or uncleavable PA (mut) together with LF, and CMG2 immunoprecipitates were probed for PA, APT2-Myc, Talin, Vinculin, Actin and CMG2. **e**, **f**, **g** APT2-KO RPE1 cells were rescued with APT2-FLAG WT, S121A, C2S or empty vector, serum-starved and stimulated with PA/LF; CMG2 immunoprecipitates were probed for PA, APT2-FLAG, Actin and CMG2. Quantification shows APT2 and Actin co-IP normalized to APT2-WT + PA = 100% (*n* = 3, mean ± SD). RPE1 cells pre-treated 4 h with DMSO, ML349 or ABD957 were analyzed in parallel and expressed relative to DMSO + PA (*n* = 4, mean ± SD). **h** Model of the CMG2 signalling switch in which ligand binding induces APT2 recruitment and CMG2 deacylation, releasing the Talin–Vinculin–Actin complex to permit endocytosis. Created in BioRender. Abrami, L. (2026) https://BioRender.com/2lsvclz.

We next tested whether ligand-induced CMG2 deacylation is mediated by APT2. We first tested whether the APT2 inhibitor ML349 would influence the TVA-to-RhoA switch. ML349 fully blocked the switch for endogenous (Fig. 6a) and ectopically expressed CMG2 (Fig. 6b), the CCA and AAC mutants (Fig. 6b), which coimmunoprecipitated with talin, vinculin and actin, but not with RhoA. The requirement for APT2 was further confirmed by APT2 silencing (Fig. 6c, Supplementary Fig. 5a) and knockout (Supplementary Fig. 5b). APT2 recruitment to CMG2 could be detected by immunoprecipitation of PA-treated cells (Fig. 6d). APT2 recruitment does not require CMG2 clustering following PA oligomerization since a PA mutant that cannot be processed by furin and therefore cannot oligomerize^32,33^, still induced APT2 recruitment (Fig. 6d). APT2 recruitment was likewise independent of CMG2 interaction with the actin cytoskeleton, as the CMG2 W396L mutant, which cannot bind Talin^21^, still recruited APT2 upon PA stimulation (Supplementary Fig. 5c).

APT2 is a soluble globular protein that can interact with membranes following a three-step process involving electrostatic interactions, insertion of a hydrophobic loop (the ß-tongue) and S-acylation on Cys2^23^. The S-acylation-deficient C2S mutant was unable to bind CMG2 (Fig. 6e,f), confirming that APT2 must itself be S-acylated to deacylate its substrates^23^. Similarly, two other APT2 mutants with impaired membrane binding capacity, the APT2^PosPatch^—in which a positively charged patch is mutated and which is unable to approach membranes through electrostatic interactions^23^, and APT2 M68E, which affects the hydrophobicity of the membrane-inserting ß-tongue^23^, also failed to be recruited to CMG2 upon ligand binding (Supplementary Fig. 5d). The catalytically inactive S121A APT2 mutant in contrast was able to bind CMG2, but failed to trigger TVA release (Fig. 6e,f), confirming the requirement for the thioesterase activity. Interestingly, inhibition of APT2 with ML349 prevented its recruitment to CMG2 (Fig. 6f, Supplementary Fig. 5e,f), while the ABHD17 inhibitor ABD957 had no effect (Fig. 6f). This was not due to an effect of ML349 on the ability of APT2 to bind membranes, since APT2 was still found in the membrane fraction upon fraction of a post nuclear supernatant generated from ML349 treated cells (Supplementary Fig. 5g), and was able to bind to liposomes in vitro in the presence of ML349 (Supplementary Fig. 5h). Since ML349 inserts into the hydrophobic acyl-chain binding pocket of APT2^34^, these results indicate that for APT2-CMG2 interactions to be detected by immunoprecipitation, a CMG2-bound acyl chain must insert into the hydrophobic pocket of APT2. APT2 recruitment always coincided with loss of binding of the actin cytoskeleton to CMG2 (Fig. 6g).

Thus, recruitment of membrane-anchored APT2 constitutes the first cytosolic response to ligand binding, and deacylation acts as the execution step of the CMG2 signalling switch, converting a cytoskeleton-anchored receptor into an internalization-competent complex (Fig. 6h).

### APT2 inhibition prevents anthrax intoxication

**Fig. 7:**
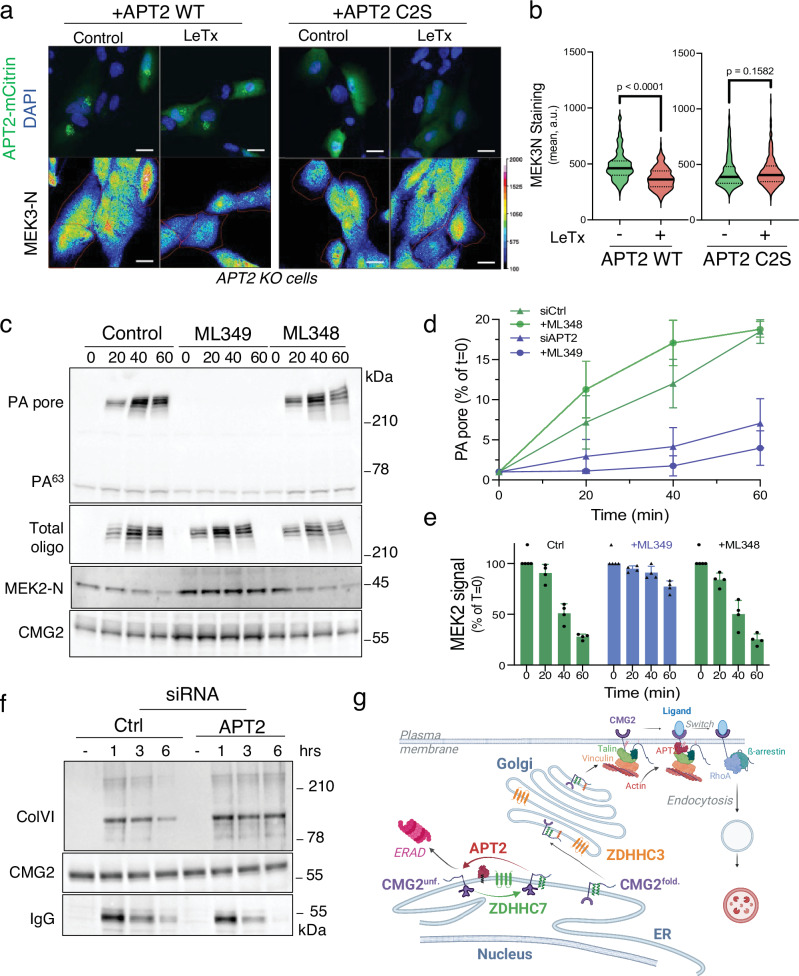
CMG2 deacylation is essential for endocytosis of Anthrax lethal toxin. **a** Representative confocal images of RPE1 APT2 KO cells, recomplemented with APT2 WT or C2A, tagged N-terminally with Citrin (upper images). Cells were incubated 1 h at 4 °C with 500 ng/ml Anthrax toxin Protective Antigen (PA) and 100 ng/ml Lethal Factor LF (jointly LeTx); cells were washed and incubated for 1 at 37 °C in serum-free medium. MEK3 was stained by immunofluorescence, and nuclei were stained with Hoechst. The APT2-citrin expressing cells are outlined in red. Scale bar = 10μm. **b** Quantification of **a**. Cells were segmented using Cellpose3 and MEK3 mean staining intensity was measured for each cell. A Kolmogoroc-test was used to test differences in the distribution of the two groups. Differences between the distributions of the two groups were assessed. The *p* value for the APT2 WT condition is approximate. **c** RPE1 cells were pre-incubated 4 h at 37 °C with ML348 or ML349. Cells were incubated 1 h at 4 °C with 500 µg/ml PA and 50 ng/ml LF and chased different indicated times at 37 °C. 40 µg of protein samples were submitted to SDS-PAGE, and analyzed by immunoblotting with rabbit anti-human CMG2, rabbit anti-PA or rabbit anti-MEK2 N-terminal. The amount of PA pore (SDS-resistant) (**d**) and cleaved MEK2N-terminal (**e**) were quantified, and set to 1 after 1 h at 4 °C before chase, and different time of chase were expressed relative to this (*n* = 4, mean ± SD). **f** RPE1 cells were silenced for 3 days with human APT2 siRNA or control siRNA. Cells were changed 24 h prior experiment in serum free medium, incubated with 1ug/ml Collagen6 indicated hours. 40 µg of protein samples were submitted to SDS-PAGE, and analyzed by immunoblotting with rabbit anti-collagen VI, anti- CMG2, and anti-IgG rabbit. **g** Cartoon of the S-acylation and deacylation events during the life cycle of CMG2. Created in BioRender. Abrami, L. (2026) https://BioRender.com/iqtwk20.

We next examined the role of APT2-mediated deacylation in anthrax toxin entry. The mode of action of anthrax lethal toxin, namely the combination of Protective Antigen (PA) with lethal Factor (LF), involves binding of the PA to CMG2/TEM8, formation of the PA63 oligomer to which LF can bind, endocytosis, and conversion of the PA oligomer into a PA pore, which is required for LF to cross endosomal membranes and ultimately reach the cytosol^35,36^. In the cytosol, LF eventually cleaves the N-terminus of MAP kinase kinases such as MEK1, 2 or 3. We first tested the role of APT2 in anthrax toxin intoxication by monitoring MEK3 levels by immunofluorescence using an antibody against its N-terminus^12^. We used APT2 KO cells which were recomplemented with either WT APT2, or APT2 C2A, which is inactive^23^. In APT2 WT expressing cells, lethal toxin (LeTx) led to a significant decrease in N-terminal MEK signal (Fig. 7a,b), as expected^37^. In contrast, the MEK3 level was unaffected by lethal toxin in APT2 C2A expressing cells (Fig. 7a,b).

We therefore monitored the different steps of lethal toxin’s mode of action biochemically. Treatment with the APT2 inhibitor ML349, but not the APT1 inhibitor ML348, drastically inhibited PA pore formation (Fig. 7c,d). This effect was not due to impaired PA oligomerization, as acidification of cell lysates prior to SDS–PAGE^22^ revealed normal oligomer assembly (Fig. 7c, Supplementary Fig 6a). Consistent with impaired PA pore formation, MEK2 cleavage was not detected in ML349-treated cells (Fig. 7c). In further agreement, APT2 silencing (Supplementary Fig 6b) and APT2 knockout (Supplementary Fig 6c) strongly reduced PA pore formation and MAPK kinase cleavage, and both phenotypes were rescued by re-expression of APT2 (Supplementary Fig. 6d). Importantly, APT2 knockout did not cause a general defect in endocytosis or in the endo-lysosomal trafficking pathway, as assessed by the normal EGF internalization and degradation observed in APT2 KO cells (Supplementary Fig. 7abc). APT2-mediated deacylation was also required for the physiological function of CMG2, as ML349 treatment impaired collagen VI degradation (Fig. 7f).

Together, these findings demonstrate that the activity of APT2 is essential for CMG2 to transition into an internalization-competent state, and is therefore essential for anthrax toxin entry into cells and collagen VI homeostasis (Fig. 7g).

### Deacylation inhibitors rescue LeTx-induced vascular phenotypes in vivo

**Fig. 8:**
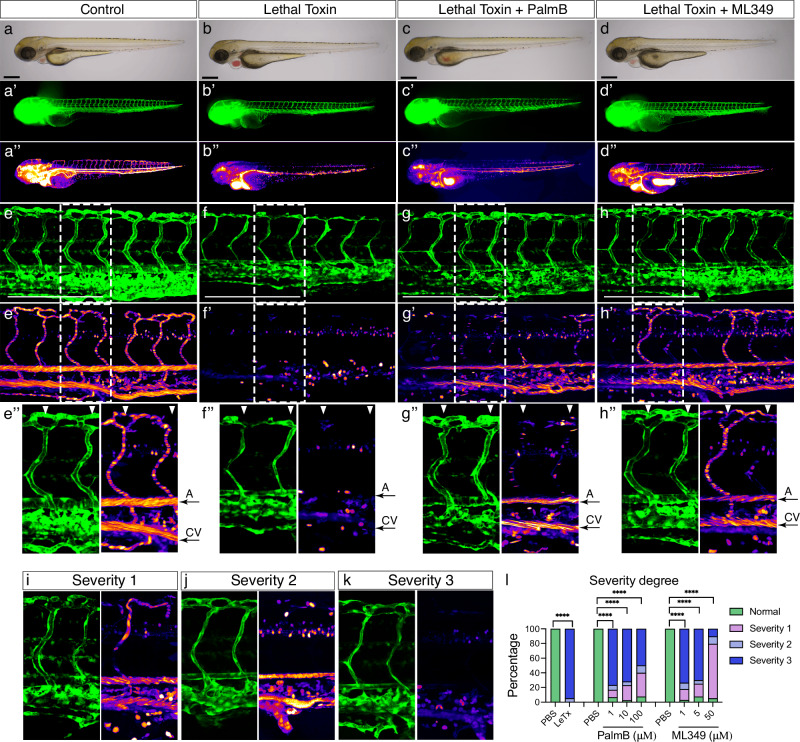
Inhibiting deacylation prevents anthrax toxin-induced vascular defects in zebrafish embryos. **a-i** Tg(*fli1*:eGFP; *gata1*:dsRed) live embryos at 3dpf representing the different experimental conditions: Control, Lethal Toxin (LeTx), and Lethal Toxin combined with either 100 μM Palmostatin B (LeTx + PalmB), or 50 μM ML349 (LeTx + ML349). fli1:eGFP expression is shown in green, and gata1:DsRed is visualized using the Fire LUT in ImageJ. **a-d** Full-embryo images showing normal macroscopic morphology and vascular patterning under all experimental conditions. Injection of LeTx dramatically impaired red blood cell circulation (**b”**), whereas co-injection of PalmB and ML349 partially prevented these circulation defects (**c”-d”**); (**a–d**) Bright-field, and (**a’–d’, a”–d”**) fluorescent channels. **e–h** Confocal images showing a constant portion of the trunk, to visualize the perfusion and blood flow in the intersegmental vessels (ISVs), aorta and caudal vein. **e”–h”** are magnifications of the framed regions in **e–h**, and indicate the position of the ISVs (arrowheads), aorta(A) and caudal vein (CV) (arrows). LeTx injection leads to narrowed ISVs (f, f”), and impaired red blood cell perfusion through ISVs, aorta and caudal vein (f’,f”). Co-injection of PalmB or ML349 rescues the circulation in the aorta and caudal vein, and partially restores ISV diameter and perfusion (**g–g”, h–h”**). **i–k** Confocal images showing representative examples of our severity degree classification (increasing from 1 to 3) observed upon LeTx injection. Severity 1 corresponds to embryos with axial flow in the aorta and caudal vein, and partial ISV perfusion; severity 2 retains axial circulation but lacks ISV perfusion; and severity 3 shows absent or markedly reduced axial blood flow. **l** Plot showing the phenotypic quantification of the different experimental conditions, demonstrating a concentration-dependent prevention of LeTx-induced defects by co-injecting PalmB or ML349. Groups were compared using Fisher’s exact test –applied to a 4×2 table (4 severity degree x 2 experimental conditions)– with a minimum number of 20 embryos per group. ****: approximative *p* < 0.0001. Scale bar represents 300μm (**a–d**, **e–h**).

To determine whether pharmacological inhibition of protein deacylation can counteract anthrax lethal toxin (LeTx) activity in vivo, we made use of a previously established zebrafish intoxication model^38^. As in mammalian models of *Bacillus anthracis* infection or injection with LeTx, toxin injection in zebrafish embryos lead to vascular dysfunction that can be visualized by in vivo imaging^38^. Zebrafish embryos co-expressing two fluorescent reporters –one in the vasculature (fli:eGFP) and the second in circulating red blood cells (gata1:dsRed) (Fig. 8a-a”)– were injected with LeTx, with and without varying concentrations of Palmostatin B or ML349 (Fig. 8).

Injection of PBS had no effect on the morphology of the vasculature or blood flow (Fig. 8a-f”, movies S1 and S5). Injection of LeTx severely impaired red blood cell circulation within 24 h post-injection, without affecting gross morphology or cardiac contractility (Fig.7 b,b”, movies S2 and S6), as previously reported^38^ and consistent with the observation that LeTx does not induce cell death in embryonic tissues or blood vessels^38^. Whole-embryo imaging revealed a complete or near-complete arrest of red blood cell circulation through the axial vessels and intersegmental vessels (ISVs) (Fig. 8b”, movie S2 and S6). Confocal imaging of the trunk vasculature further revealed a marked reduction in ISV diameter in LeTx-injected embryos (Fig. 8 f,f” vs. e,e”).

To enable systematic and reproducible phenotypic scoring, LeTx-induced circulatory defects were classified into three severity degree based on the perfusion of the vasculature by red blood cell, as illustrated by representative confocal images (Fig. 8i-k). Severity grade 1 corresponds to embryos retaining axial blood flow in the dorsal aorta and caudal vein, with partial red blood cell perfusion of the ISVs. Grade 2 describes embryos with preserved axial circulation but a complete absence of ISV perfusion. Grade 3, the most severe phenotype, is characterized by the absence or markedly reduced red blood cell flow in the axial vessels.

We next co-injected LeTx together with Palmostatin B. We observed a concentration-dependent rescue of red blood cell circulation (Fig. 8l, movie S3 and S7), with a decrease in grade 3 to 77, 72 and 50% at 1, 10 and 100 µM respectively, with a concomitant increase in grade 1 and grade 2 phenotypes. At 100 µM, 33% of Palmostatin B-treated embryos achieved grade 1 rescue. Confocal imaging confirmed that partial restoration of red blood cell circulation in the axial vessels was accompanied by a recovery of ISV diameter and partial re-establishment of ISV perfusion (Fig. 8g–g”).

ML349 was significant more potent in rescuing the embryo phenotype (Fig. 8l, movie S4 and S8). At 1 µM and 5 µM, the proportion of severely affected embryos remained elevated at 74% and 70%, respectively, with limited grade 1 rescue (3% and 8%). At 50 µM, however, a striking phenotypic redistribution was observed: the proportion of grade 3 embryos collapsed to 10%, while 74% of embryos achieved grade 1 rescue and 10% grade 2, representing near-complete restoration of red blood cell circulation at this concentration (Fig. 8d”). Confocal imaging corroborated these findings, demonstrating substantial recovery of ISV diameter and red blood cell perfusion in ML349-treated embryos at 50 µM, approaching values observed in PBS-injected controls (Fig. 8h–h”).

Taken together, these results demonstrate that both Palmostatin B and ML349 rescue LeTx-induced impairment of red blood cell circulation in a concentration-dependent manner in vivo, with ML349 exhibiting markedly greater effect (Fig. 8l).

Notably, in this co-injection paradigm, inhibitors must enter cells and inhibit deacylation while the toxin is simultaneously available for internalization to exert its effects. The observed rescue is therefore particularly striking. These findings support the idea that inhibition of deacylation can counteract toxin activity in vivo and suggest that this approach may have therapeutic potential in the context of infection, where toxin exposure occurs progressively rather than as a single bolus.

## Discussion

CMG2 is a multifunctional receptor that links the extracellular matrix to cytoskeletal signalling and endocytosis, and is also hijacked as the primary host receptor by anthrax toxin. Here we show that CMG2 biogenesis and function are controlled by sequential S-acylation–deacylation cycles acting across the secretory and endocytic pathways. These cycles enable CMG2 to adopt distinct conformational or interaction states that correspond to discrete stages of its lifecycle: folding in the ER, transport to the plasma membrane, and ligand-induced signalling and internalization. The lipidation events we describe therefore couple CMG2 biogenesis with its function as a signalling and endocytic receptor at the plasma membrane.

Our findings demonstrate that S-acylation of the juxtamembrane cysteines by ZDHHC7 in the ER promotes productive folding by preventing premature degradation of newly synthesized CMG2 by ERAD. Conversely, APT2-mediated removal of these acyl chains renders folding intermediates vulnerable to ERAD. Thus, acute APT2 inhibition increases CMG2 levels in cells and in vivo. These observations establish S-acylation as a key positive determinant of CMG2 protein homeostasis. Since a subset of Hyaline Fibromatosis Syndrome-associated missense mutations result in impaired CMG2 folding kinetics, chronic partial APT2 inhibition represents a rational therapeutic strategy to increase CMG2 expression in these patients. This mechanism is conceptually related to pharmacological strategies aimed at stabilizing CFTR and other folding-compromised mutated transmembrane proteins in the ER.

After ER exit, a second S-acylation event, mediated by ZDHHC3 in the Golgi on Cys479, allows CMG2 to interact with Arf6 and traffic to the plasma membrane. This indicates that CMG2 biogenesis is regulated not only at the folding stage but also during transport through the secretory pathway, with distinct S-acylation events conferring sequential benefits.

At the cell surface, we find that CMG2 must be S-acylated for the ligand-induced release from Talin-Vinculin-Actin and for the recruitment of RhoA, and other proteins required for signalling and endocytosis such as β-arrestin, mDia and myosin light chain^11,21,22^. These data support a model in which S-acylation constrains the cytoplasmic tail in a conformation that is competent to propagate the ligand-induced conformational change across the transmembrane helix, allowing CMG2 to reposition its cytosolic interactions. In the absence of S-acylation, ligand binding still occurs but the conformational signal fails to cross the membrane, preventing receptor activation.

Finally, we show that ligand binding triggers the recruitment of APT2 to CMG2, involving the sequential insertion of the CMG2-bound acyl chains into the hydrophobic acyl-chain binding pocket of APT2. APT2 recruitment precedes TVA release, and mediates CMG2 deacylation, which converts the receptor from a cytoskeleton-anchored to actin regulatory and internalization-competent state. While regulated endocytosis of full-length CMG2 requires this S-acylation dependent release from cytoskeletal interactions, such regulatory constraints can be bypassed by truncation mutants that lack the cytosolic tail^39,40^.

Consistent with our observations, genetic or pharmacological inhibition of APT2 blocks anthrax lethal toxin internalization, pore formation in endosomes, and downstream MAPK cleavage in the cytosol. Similarly, in the physiological setting, APT2 inhibition prevents CMG2-mediated collagen VI degradation. Finally, APT2 inhibition has a protective effect in zebrafish towards Lethal Toxin induced vascular damage. These results demonstrate that deacylation is not simply the reversal of a modification that favours biogenesis, but a signalling execution step.

The opposing effects of APT2 on CMG2 biogenesis and ligand-induced internalization highlight the stage-specific, temporally separated, nature of S-acylation-deacylation cycles and therefore that APT2 inhibition may have different outcomes depending on timing and duration. Post-exposure prophylaxis toward *Bacillus anthracis* infection would require an acute and maximal inhibition of APT2 activity, allowing the immune system to clear the infection, without long-term consequences due to receptor inactivation. In contrast, HFS treatment would require a chronic low concentration or intermittent treatment, allowing an increase in CMG2 protein expression, while at least intermittently retaining CMG2’s signalling ability and ECM regulation. More generally, these findings underscore the need to consider temporal dynamics when targeting lipid-modifying enzymes therapeutically.

In summary, this work identifies coordinated and temporally ordered S-acylation-deacylation cycles as a central regulatory principle controlling CMG2 folding, trafficking, and function, both physiological and pathological. Hence, this study demonstrates that S-acylation-deacylation cycles can control sequential trafficking steps along the biosynthetic and endocytic pathway. Beyond clarifying the molecular logic of CMG2 S-acylation, our findings establish APT2 as a druggable regulator of receptor function, with implications for both HFS therapy and anthrax infection, as well as other genetic diseases affecting the folding of membrane proteins, 50 to 70% of which undergo S-acylation^41–44^.

## Methods

With the exception of the experiments in Fig. 3j and Supplementary Fig. 3h,i and Fig. 8, none of the experiments described in this manuscript require ethical approval. Experiments in mice depicted in Fig. 3j and Supplementary Fig. 3h,i were approved by Swiss cantonal veterinary office (authorization VD3794b). For experiments described in Fig. 8 on zebrafish embryos, all experimental procedures were conducted in accordance with Swiss federal legislation on animal experimentation (Animal Welfare Act, TSchG; Animal Welfare Ordinance, TSchV), under authorization granted by the Federal Food Safety and Veterinary Office (FSVO) of the Canton of Vaud, Switzerland (authorization no. VD-H23).

### Cell lines

HeLa cells (ATCC CCL-2) were grown in Modified Eagle’s Medium (Sigma Life Science) supplemented with 10% FBS, 2 mM of L-glutamine, non-essential amino acids, penicillin, and streptomycin (GIBCO). Primary fibroblasts and RPE1 (ATCC CRL-4000) and RPE1 KO APT2^23^ were grown in Dulbecco’s Modified Eagle Medium (Sigma Life Science) supplemented with 10% FBS, penicillin, and streptomycin (GIBCO).

### Plasmids and transfection

Human CMG2 (isoform 4, UniProt P58335-4) WT was cloned in a pcDNA3.5/V5-HIS-TOPO expression vector or in pHS003-EGFP. Cystein mutants and L45P, G105D, I186T, L349R, W396L mutant were generated in pcDNA3.5/V5-HIS-TOPO using the QuikChange Site-directed Mutagenesis Kit (Agilent) according to the manufacturer’s protocol.

Human APT2/LYPLA2 WT was cloned in a pCMV6-myc-flag expression vector. S121A, M68E, Pospatch and C2S APT2 mutants were generated in pCMV6-myc-flag using QuikChange Site-directed Mutagenesis Kit (Agilent) according to the manufacturer’s protocol.

MYC fusion human ZDHHC3 and human ZDHHC7 were cloned in pcDNA3.1^45^.

Plasmids were transfected into cells for 24 h or 48 h, depending on the experiment, using *TRANS*IT–X2 according to manufacturer’s protocol (MIRUS).

Verified siRNA for human ZDHHCs and for human APT1 and APT2 were purchased from Qiagen. APT1 target sequence: 5’-AACAAACTTATGGGTAATAAA-3’. APT2 target sequence: 5’-CAGCTGCTTCTCAGTCATGAA-3’. ARF6 target sequence: 5’-TGCGACCACTATGATAATATT-3’. The pool of siRNA were done as followed: Mix1 (ZDHHC1 (5’-ACCGGCTGTGATGCTCCAATA-3’) + ZDHHC3 (5’-TCCGTTCTCATGAATGTTTAA-3’) + ZDHHC7 (5’-CCCGTGGTTACTATGAATGTA-3’) + ZDHHC13 (5’-CAGCATAGTAGCCTTTCTATA-3’) + ZDHHC17 (5’-CAGTACCTGTTTGATACGAAA-3’); Mix2 (ZDHHC2 (5’-TAGCTACTGCTAGAAGTCTTA-3’) + ZDHHC6 (5’-GAGGTTTACGATACTGGTTAT-3’) + ZDHHC4 (5’-ATGGATTGCTTCATTACCTTT-3’) + ZDHHC16 (5’-CTCGGGTGCTCTTACCTTCTA-3’)); Mix3 (ZDHHC5 (5’-ACCACCATTGCCAGACTACAA-3’) + ZDHHC8(5’- CCGGGCTCCGCTGTACAAGAA-3’) + ZDHHC15 (5’-ATCGCTATATCAAGTATCTAA-3’) + ZDHHC20 (5’-TACCTGTTATGAGTTGCCTATA-3’)); Mix4 (ZDHHC9 (5’-CTCAACCAGACAACCAATGAA-3’) + ZDHHC12 (5’-CAGATACTGCCTGGTGCTGCA-3’) + ZDHHC18 (5’-AAGCCTGATGCCAGCATGGTA-3’)); Mix5 (ZDHHC11 (5’-CGCGTGGAAATACATTGCCTA-3’) + ZDHHC23 (5’-CTGCGAGTACATAGATCGGAA-3’) + ZDHHC24 (5’-CCGCTGCGTGGGGCTTCGGCAA-3’); Mix6 (ZDHHC14 (5’-CTGGGTGTCCTCGGCAAAGTT-3’) + ZDHHC19 (5’-GACCCTGGCATCTTACATCAA-3’) + ZDHHC21 (5’-GTGGGACTAATACAAGATCTA-3’) + ZDHHC22 (5’-TGGGTTCATTTATGCCCTATA-3’).

As a control siRNA, a sequence targeting the viral glycoprotein VSV-G (5’-ATTGAACAAACGAAACAAGGA-3’) was used. Transfections of 50 nM of siRNA were carried out using *TRANS*IT-X2 (MIRUS), and the cells were analyzed at least 72 h after transfection.

### Quantitative RT-PCR

RNA was extracted from cells or tissues with RNeasykit (Qiagen). Reverse transcription was performed with random hexamers and superscript II (Invitrogen). SYBR green Master Mix was used to perform real time PCR with housekeeping genes TBP and ALAS1 and the specific following primers:

-h ZDHHC3 CTGCAGCATCAAGCCCGACC; CGGTGCATTCGGAAGATGGACC

-h ZDHHC7 CCTGTGCCTTGAGGGTCTTCTG; GAAGCCCACATGGGAGCGG

-h APT1 GCGGCAATAACATGTCAACC; CATGCAGGAAAATCACCGCA

-h APT2 CCCTGTGACCCTCAACATGA; CCCATCAGGTCAAACCAGGA

-human CMG2: GGCAAGTTGGACGGTCTGGTG; CACTAGCCCCAAGTGACCTGG

-human TEM8: GGCCACTCTGGTGCTCATCTG; CCAGTGGTGCAGCACACTTCC

-m LRP6. CAGAAGAGCGCCATCAACCGC; GGATGCCCTGCCCACTACTC

-mCMG2:CCCTGAAATGAGATTGTCCTTCATTGTG; CCTTTGCCAATTTTGTACCTGTCTCCAG

-mouse TEM8: GGGGGACCAGCTTGCTACGG, GGAAGTGTGCTGCACCACTGG

Results were expressed as 2^*^(-ΔΔCt)*100%.

### Antibodies and reagents

Anti-mouse CMG2 monoclonal antibody 8F7 was generated by the genetic immunization of rats with a mouse CMG2 construct^46^ (Genovac, Germany). The other antibodies were commercially available: goat anti-human CMG2 (R&D, AF2940, RRID: AB_2056740); rabbit anti-human CMG2 (Proteintech, ref: 16723-1AP, RRID: AB_2056741); goat anti-human TEM8 (R&D, ref: AF3886, RRID: AB_2056726); rabbit anti-ARF6 (LSBio, ref: LS-C81881,RRID: AB_2058487); mouse anti-TRAP alpha (Santa Cruz, ref: 100314,RRID: AB_1130631); anti-V5 (Invitrogen, 46-0705, RRID: AB_2556564); anti-GFP (Takara Bio, ref: 632592, RRID: AB_2336883); mouse anti-Myc (SIGMA, 9E10 M4439, RRID: AB_439694), anti-human ZDHHC3 (Abcam, ab31837, RRID: AB_742236); anti-GM130 (BD biosciences, 610823, RRID:AB_398142); mouse anti-GAPDH (Acris Antibodies, 4A1-MA0100, RRID:_AB_1874646); anti-actin (Merck Millipore, ref: MAB1501, RRID: AB_2223041); anti-Talin (Sigma, ref: T3287, RRID: AB_477572); anti-Vinculin (Sigma, ref. V9131, RRID: AB_477629); anti-phospho tyrosine (Millipore, ref:05-1050, RRID: AB_916371); anti-RhoA (Cell signalling, 67B9, ref: 2117, RRID: AB_10693922); anti-SRC (Abcam, ref: 16885, RRID: AB_443522); anti-Anthrax Protective Antigen (List Biological Laboratories, ref: 771B); anti-MEK2 against N-terminal (Santa Cruz, ref:12578, RRID: AB_648958, discontinued); anti-MEK3 against N-terminal (Santa Cruz, ref:960, RRID: AB_631928, discontinued); anti-collagen VI (Fitzgerald laboratories, ref: 70R-CR009X, RRID: AB_1283876); anti-calnexin (Millipore, ref:MAB3126, RRID: AB_2069152); Anti-IgG (Santa Cruz, ref: SC-2025, RRID: AB_737182), HRP-conjugated secondary antibodies (Pierce); and for immunoprecipitation protein G beads were purchased from GE Healthcare.

Anti MEK1-N terminal antibodies were produced in our laboratory (Abrami et al., 2008) with Covalab, against N-terminal 20 amino acids cleaved by LF.

Brefeldin A (Sigma) was used at 2 µg/ml, Bafilomycin A (Sigma) was used at 100 nM, MG132 (Sigma) was used at 10uM and Cycloheximide (Sigma) was used 10 µg/ml 1 h before and during 3H-palmitic acid experiments. ABD957, ML348 and ML349 (Cayman Chemical company) were used at 10 µM indicated times. Palmostatin B (Merk) was used at 50 µM indicated times.

Wild type PA (Anthrax toxin Protective Antigen) and LF (Anthrax toxin Lethal Factor) were produced in our laboratory by overexpression in *E.coli* as described (Feld et al., 2012)^47^. Collagen VI was a gift from Bonaldo Laboratory^48^.

### Western blotting

Cells were washed three times in 1x PBS at 4 °C and lysed in Buffer (1x PBS, 1% Triton X-100, and protease inhibitor cocktail; Roche) for 30 min on ice. Lysates were then spun down at 5000 rpm on a table-top centrifuge, and the protein content of supernatant were determined, the samples were boiled in Laemmli buffer for 5 min before separation via SDS-PAGE and western blotting against the different anti-bodies used in this study. Western blots were developed using the ECL protocol (Thermo Fisher) and imaged on a Fusion Solo from Vilber Lourmat. Densitometric analysis was performed using the software Bio-1D from the manufacturer.

All uncropped and unprocessed blot are provided in the Supplementary Information file.

### Acyl-RAC capture assay

Protein S-acylation was assessed by the Acyl-RAC assay^49^ as previously described^50^ with some modifications. Mice tissues were lysed in 400 μl buffer (0.5% Triton-X100, 25 mM HEPES, 25 mM NaCl, 1 mM EDTA, pH 7.4, and protease inhibitor cocktail). Then, 200 μl of blocking buffer (100 mM HEPES, 1 mM EDTA, 87.5 mM SDS, and 1.5% [v/v] methyl methanethiosulfonate [MMTS]) was added to the lysates and incubated for 4 h at 40 °C to block free the SH groups with MMTS. Proteins were acetone precipitated and resuspended in buffer (100 mM HEPES, 1 mM EDTA, 35 mM SDS). For treatment with hydroxylamine (NH2OH) and capture by Thiopropyl Sepharose® beads, 2 M of hydroxylamine was added together with the beads (previously activated for 15 min with water) to a final concentration of 0.5 M of hydroxylamine and 10% (w/v) beads. As a negative control, 2 M Tris was used instead of hydroxylamine. These samples were then incubated overnight at room temperature on a rotating wheel. After washes, the proteins were eluted from the beads by incubation in 40 μl SDS sample buffer with ß-mercaptoethanol for 5 min at 95 °C. Finally, samples were separated by SDS-PAGE and analyzed by immunoblotting. A fraction of the tissue lysate was saved as the input.

### Acyl-peg-exchange

Cells were lysed and incubated treated in 400 µl buffer (0.2% SDS, 0.5% Tx-100, 100 mM HEPES, 1 mM EDTA, 100 mM NEM pH 7.5, and protease inhibitor cocktail) for 2 h at 40° C. To remove excess unreacted NEM, proteins were acetone precipitated and re-suspended in buffer (100 mM HEPES, 1 mM EDTA, 1% SDS, pH 7.5). Previously palmitoylated cysteines were revealed by treatment with 500 mM hydroxylamine (NH_2_OH) for 1 h at 37 °C. Cell lysates were desalted using Zebra spin columns and incubated 1 h at 37 C with 2 mM 5 kDa methoxypolyethylene glycol maleimide mPEG. Reaction was stopped by incubation in SDS sample buffer for 5 min at 95 °C. Samples were separated by SDS-PAGE and analyzed by Western blot.

### ^3^H-palmitic acid radiolabeling experiments and immunoprecipitation

HeLa, RPE1, human fibroblast cells were transfected or not with different constructs, incubated for 2 h in Glasgow minimal essential medium (IM; buffered with 10 mM HEPES, pH 7.4) with 200 µCi/ml of ^3^H palmitic acid (9,10-^3^H[N]) (American Radiolabeled Chemicals, Inc.)^50^. The cells were washed, and incubated in DMEM complete medium for the indicated time of chase or directly lysed for immunoprecipitation with the indicated antibodies.

For immunoprecipitation, cells were washed three times in PBS, lysed 30 min at 4 °C in the IP buffer (0.5% Nonidet P-40, 500 mM Tris pH 7.4, 20 mM EDTA, 10 mM NaF, 2 mM benzamidin and protease inhibitor cocktail [Roche]), and centrifuged 3 min at 5000 rpm. Supernatants were subjected to preclearing with Protein G Sepharose beads prior to the immunoprecipitation reaction. Supernatants were incubated overnight with the appropriate antibodies (V5 or goat anti-human CMG2) and Protein G Sepharose beads. After immunoprecipitation, washed beads were incubated for 5 min at 90 °C in reducing sample buffer prior to 4 − 20% gradient SDS-PAGE. Gels were incubated 30 min in a fixative solution (25% isopropanol, 65% H2O, 10% acetic acid), followed by a 30 min incubation with the signal enhancer Amplify NAMP100 (GE Healthcare). The radiolabeled products were imaged using a Typhoon phosphoimager and quantified using a Typhoon Imager (ImageQuanTool, GE Healthcare). The images shown for ^3^H-palmitate labeling were obtained using fluorography on film.

### Membrane separation from cytosolic fraction

Hela cells were washed with PBS and homogenized through a 22 G injection needle in HB (2.9 mM imidazole and 250 mM sucrose, pH 7.4 and protease inhibitor). After centrifugation, the supernatant was collected as Post Nuclear Supernatant (PNS). PNS was centrifuged for 30 min at 100,000 *g* in Sorvall MX150 ultracentrifuge. The pellet and supernatant (sup.) were dissolved in an equal volume of reducing sample buffer and incubated for 5 min at 95 °C before separation on a 4–20% gradient SDS–PAGE^23^.

### Liposome flotation assay

APT2 purified protein (50 µg) were incubated with 200 µl of liposomes (2:2:1 molar ratio of L-apha-phosphatidylcholine (Avanti 131601 C), L-alpha-phosphatidylserine (Avanti 840032 C); L-alpha-phosphatidylethanolamine (Avanti 840026 C)) for 2 h at 10 °C. The mixture was adjusted to 40% sucrose and loaded on the bottom of a sucrose step gradient (40%/30%/10% sucrose). After 1 h centrifugation at 100,000 g, 10 µl of each interface were collected and analyze by Coomassie bue after 4-20% SDS-PAGE (interface 1: between 10 and 30%, interface 2: between 30 and 40% and 3, bottom).

### Immunofluorescence microscopy

Cells were fixed in 4% paraformaldehyde (15 min) and permeabilized permeabilized with 0.05 % Saponin in PBS with 2% bovine serum albumin. Antibodies were diluted in PBS containing 0.05 % Saponin and 1% BSA. Coverslips were incubated overnight at 4 °C with primary antibodies, washed three times in PBS, and incubated 1 h with secondary antibodies. Coverslips were mounted onto microscope slides with Fluoromount-G^TM^ (00-4958-02, Invitrogen). Images were collected using eiter a spinning disk confocal (Inverted Nikon Eclipse Ti2 motorized) equipped with a 60x Plan apo Lambda D oil immersion objective and a sCMOS camera (Teledyne Kinetix M-C) for MEK3 cleavage experiment or a confocal laser-scanning microscope (Zeiss LSM 700) and processed using Fiji™ software.

For automated microscopy, HeLa Cells were seeded at  5× 10^3^ cells per well in Ibidi 96-well plates, silencing was performed directly in presence of siRNAs in transfection mix containing *TRANS*IT-X2 (MIRUS). After 72 h, the cells were fixed using 3% PFA, washed and subjected to immunofluorescence.

### EGF uptake and degradation by automated spinning disk confocal microscopy

RPE1 and RPE1 APT2 KO Cells were seeded at 1× 10^4^ cells per well in Ibidi 96-well plates. After 24 h, cells were transfected using *TRANS*IT-X2 (MIRUS) with 0.1 μg of APT2-citrin plasmid per well. 24 h later, the medium was replaced by warm medium containing 0.5 μg/ml of A647-EGF and the cells were incubated 15 min at 37 °C, washed twice and further incubated in warm medium at 37 °C for the specified times of the pulse chase experiment and finally fixed 15 min with 3% PFA^51^. After the fixation, the cells were incubated with PBS containing 0.01% saponin, 0.5% BSA and Hoechst (50 µg/ml). The plate was washed 3 times using a Biotek EL406 plate washer and the software. The images were segmented to generate relevant masks, which were then applied on the fluorescence images acquired using a 40X water immersion objective with 1.15NA on a spinning disk confocal automated microscope (Molecular Device). For each FOV, 5 stacks separated by 1 μm were acquired and combined into a maximum intensity projection image. Analysis of the images was done via the MetaXpress Custom Module editor images to extract the relevant measurements. To facilitate segmentation of EGF vesicles, we applied the top hat deconvolution method, to reduce the background noise and highlight bright granules.

In all of the automated microscopy experiments, a minimum of 1,000 cells per biological replicate were imaged and analyzed using our high-throughput microscopy set-up. No statistical methods were used to predetermine the sample sizes. The data were tested for Gaussian distribution using the Kolmogorov–Smirnov test (with a Dallal–Wilkinson–Lillie test for the *P* value). For non-Gaussian distributions, the following non-parametric tests were used: two-tailed Mann–Whitney U tests to compare two conditions or one-way analysis of variance (ANOVA; Kruskal–Wallis test) with a Dunn’s test to compare more than two data groups. For Gaussian distributions, two-tailed Student’s *t*-test were used for comparison of the means between two conditions and parametric one-way ANOVA with a Tukey’s honestly significant difference or Dunnett’s multiple comparison tests for comparisons between more than two data groups. The significance of the mean comparisons is represented on graphs by asterisks; *P  <  0.05; **P  <  0.01 and ***P  <  0.001.

### Surface biotinylation

Surface biotinylation was performed on RPE1 cells with endogenous CMG2 or on Hela cells transfected with CMG2. After 72 h transfection, cells were allowed to cool down shaking at 4 °C for 15 min to arrest endocytosis. Cells were then washed three times with cold PBS and treated with EZ-Link Sulfo-NHS-SS-Biotin No weight (Thermo Scientific) for 30 min shaking at 4 °C. Cells were then washed 3 times for 5 min with 100 mM NH4Cl and lysed in IP Buffer for 1 h at 4 °C. Lysate were then centrifuged for 5 min at 5000 rpm and the supernatant incubated with streptavidin agarose beads (Sigma) overnight on a wheel at 4 °C. Western blots were developed using the ECL protocol (Thermo Fisher) and imaged on a Fusion Solo from Vilber Lourmat. Densitometric analysis was performed using the software Bio-1D from the manufacturer.

### Mouse experiments

ML349 was dissolved in 50%PEG300 in PBS. ML349 was injected every 2 days at 40 mg/kg intraperitoneally in 11-week-old male and female C57BL/6-K18 mice^52,53^,. Control mice were injected with same volume of PEG300. After different days of treatment, different organs were collected and protein were extracted.

### Zebrafish husbandry and embryo collection

Adult zebrafish (*Danio rerio*) were maintained in polycarbonate tanks at a density of 8 fish per liter, connected to a flow-through recirculating system under standard husbandry conditions^54^. Water temperature was kept at 26 °C under a 14 h light/10 h dark photoperiod. Water quality parameters were continuously monitored and maintained at a pH of 7.5 (adjusted with sodium bicarbonate), and a conductivity of 600 µS/cm^2^ (adjusted with calcium chloride and Instant Ocean). Adult fish were fed five times daily with *Artemia* nauplii and dry pellets (Sparos).

The transgenic lines Tg(*fli1*:eGFP)^*y1*^ ^55 ^and Tg(*gata1*:DsRed)^*sd2 *^^56^ were obtained from the Mercader laboratory (University of Bern, Switzerland).

Embryos were obtained by natural spawning and raised in system water at 28.5 °C. To inhibit pigmentation and facilitate live imaging, embryos were treated with 0.002% N-phenylthiourea (PTU) from 1 day post-fertilization (dpf) onwards. All experiments were performed on embryos younger than 4 dpf, a stage at which sex determination is not yet possible.

### Microinjection of Zebrafish embryos and imaging

At 2 dpf, zebrafish embryos were mechanically dechorionated and screened for co-expression of the *fli1*:eGFP and *gata1*:DsRed transgenes. Shortly before injection, embryos were anesthetized with 0.016% tricaine (MS-222), immobilized by embedding them in 0.8% low-melting-point agarose, and mounted laterally on a glass-bottom dish.

Micro-injections were performed into the systemic circulation via the duct of Cuvier using a gas-driven microinjector (PV820, World Precision Instruments) coupled to a micromanipulator (IM-300, Narishige), with pulled borosilicate glass microcapillary pipettes (World Precision Instruments), and visualized under a SZ61 stereomicroscope (Olympus).

Anthrax lethal toxin (LeTx) was prepared by combining 9.25 μM lethal factor (LF) and 6.25 μM protective antigen (PA), supplemented with 0.1% phenol red and kept on ice until use. For deacylation inhibition treatments, LeTx was mixed with either Palmostatin B or ML349 at three different concentrations (1, 10 and 100 μM for Palmostatin B, and 1, 5, and 50 μM for ML349). Each embryo received two consecutive pressure pulses of approximately 2 nL each, for a total injected volume of 4 nL, resulting in a final quantity of 37 fmol of LF and 25 fmol of PA per embryo as described in Bolcome et al., 2008^38^. Control embryos received an equivalent volume of phosphate-buffered saline (PBS). Timing for downstream analyses was initiated immediately following the last pulse.

Following injection, embryos were transferred to fresh system water containing 0.002% PTU and incubated at 28.5 °C. Phenotypic scoring was performed 24 h post-injection. For imaging, embryos were embedded in 0.8% low-melting-point agarose in glass-bottom dishes (MatTek Corporation, USA) and anesthetized with 0.008% tricaine. Fluorescence images and time-lapse movies were acquired using an MVX10 MacroView microscope (Olympus) equipped with an MVPLAPO 1x objective and a SOLA solid-state light engine (Lumencor), operated with cellSens Standard 2.3 software (Olympus).

To visualize perfusion and blood flow in the intersegmental vessels, aorta and caudal vein, same embryos were imaged with an inverted LEICA confocal SP8 microscope equipped with a HC FLUOTAR 25×0.95NA water objective, using hybrid photon counting detectors (HyD). For each embryo a z-stack was captured with a 1 μm step size. Each image was acquired using the same settings across all samples. Images were further processed with ImageJ v1.54p. Figures show maximum intensity projections of 40-50 μm depth corresponding to approximately half of the embryo volume, including the aorta, caudal vein, and ISVs. Crops were performed using a constant ROI size.

### Fish data analysis

Data were expressed as the percentage of affected embryos relative to total embryos per treatment group. A minimum of 20 embryos were analysed in each experimental condition, across 4 independent repetitions.

### Statistics and reproducibility

No statistical method was used to predetermine sample size. No data were excluded from the analyses; The experiments were not randomized; The Investigators were not blinded to allocation during experiments and outcome assessment.

### Reporting summary

Further information on research design is available in the Nature Portfolio Reporting Summary linked to this article.

## Supplementary information

Supplementary Information

Description of Supplementary Information

Supplementary Movie 1

Supplementary Movie 2

Supplementary Movie 3

Supplementary Movie 4

Supplementary Movie 5

Supplementary Movie 6

Supplementary Movie 7

Supplementary Movie 8

Reporting Summary

Transparent Peer Review file

## Source data

Source Data

## Data Availability

The source data underlying all Figures, and Supplementary Figs. are provided in the Source Data file. All uncropped and unprocessed blot are provided in the Supplementary Information file. Source data are provided with this paper.
