# Supplementary material for: Dynamic S-acylation controls CMG2 maturation extracellular matrix regulation and anthrax toxin susceptibility in vivo
Source: Nat Commun. 2026 Jul 6;17:8364. doi: 10.1038/s41467-026-75028-2 (PMC13473502; doi:10.1038/s41467-026-75028-2)
Supplement: Supplementary file 1 [file 41467_2026_75028_MOESM11_ESM.pdf]

nature portfolio

Corresponding author(s): Gisou van der Goot

Last updated by author(s): 16/06/26

Reporting Summary

Nature Portfolio wishes to improve the reproducibility of the work that we publish. This form provides structure for consistency and transparency in reporting. For further information on Nature Portfolio policies, see our Editorial Policies and the Editorial Policy Checklist.

Statistics

For all statistical analyses, confirm that the following items are present in the figure legend, table legend, main text, or Methods section.

n/a	Confirmed	
☐	☒	The exact sample size (*n*) for each experimental group/condition, given as a discrete number and unit of measurement
☐	☒	A statement on whether measurements were taken from distinct samples or whether the same sample was measured repeatedly
☐	☒	The statistical test(s) used AND whether they are one- or two-sided<br>*Only common tests should be described solely by name; describe more complex techniques in the Methods section.*
☒	☐	A description of all covariates tested
☒	☐	A description of any assumptions or corrections, such as tests of normality and adjustment for multiple comparisons
☐	☒	A full description of the statistical parameters including central tendency (e.g. means) or other basic estimates (e.g. regression coefficient) AND variation (e.g. standard deviation) or associated estimates of uncertainty (e.g. confidence intervals)
☒	☐	For null hypothesis testing, the test statistic (e.g. *F*, *t*, *r*) with confidence intervals, effect sizes, degrees of freedom and *P* value noted<br>*Give P values as exact values whenever suitable.*
☒	☐	For Bayesian analysis, information on the choice of priors and Markov chain Monte Carlo settings
☒	☐	For hierarchical and complex designs, identification of the appropriate level for tests and full reporting of outcomes
☒	☐	Estimates of effect sizes (e.g. Cohen's *d*, Pearson's *r*), indicating how they were calculated

Our web collection on statistics for biologists contains articles on many of the points above.

Software and code

Policy information about availability of computer code

Data collection — *Provide a description of all commercial, open source and custom code used to collect the data in this study, specifying the version used OR state that no software was used.*

Data analysis — *Provide a description of all commercial, open source and custom code used to analyse the data in this study, specifying the version used OR state that no software was used.*

For manuscripts utilizing custom algorithms or software that are central to the research but not yet described in published literature, software must be made available to editors and reviewers. We strongly encourage code deposition in a community repository (e.g. GitHub). See the Nature Portfolio guidelines for submitting code & software for further information.

Data

Policy information about availability of data

All manuscripts must include a data availability statement. This statement should provide the following information, where applicable:

- Accession codes, unique identifiers, or web links for publicly available datasets
- A description of any restrictions on data availability
- For clinical datasets or third party data, please ensure that the statement adheres to our policy

The data that support the findings of this study are available from the corresponding author upon reasonable request. (we are happy to attach the source files with the manuscript)

Research involving human participants, their data, or biological material

Policy information about studies with human participants or human data. See also policy information about sex, gender (identity/presentation), and sexual orientation and race, ethnicity and racism.

Reporting on sex and gender	*Use the terms sex (biological attribute) and gender (shaped by social and cultural circumstances) carefully in order to avoid confusing both terms. Indicate if findings apply to only one sex or gender; describe whether sex and gender were considered in study design; whether sex and/or gender was determined based on self-reporting or assigned and methods used.*<br>*Provide in the source data disaggregated sex and gender data, where this information has been collected, and if consent has been obtained for sharing of individual-level data; provide overall numbers in this Reporting Summary. Please state if this information has not been collected.*<br>*Report sex- and gender-based analyses where performed, justify reasons for lack of sex- and gender-based analysis.*
Reporting on race, ethnicity, or other socially relevant groupings	*Please specify the socially constructed or socially relevant categorization variable(s) used in your manuscript and explain why they were used. Please note that such variables should not be used as proxies for other socially constructed/relevant variables (for example, race or ethnicity should not be used as a proxy for socioeconomic status).*<br>*Provide clear definitions of the relevant terms used, how they were provided (by the participants/respondents, the researchers, or third parties), and the method(s) used to classify people into the different categories (e.g. self-report, census or administrative data, social media data, etc.)*<br>*Please provide details about how you controlled for confounding variables in your analyses.*
Population characteristics	*Describe the covariate-relevant population characteristics of the human research participants (e.g. age, genotypic information, past and current diagnosis and treatment categories). If you filled out the behavioural & social sciences study design questions and have nothing to add here, write "See above."*
Recruitment	*Describe how participants were recruited. Outline any potential self-selection bias or other biases that may be present and how these are likely to impact results.*
Ethics oversight	*Identify the organization(s) that approved the study protocol.*

Note that full information on the approval of the study protocol must also be provided in the manuscript.

Field-specific reporting

Please select the one below that is the best fit for your research. If you are not sure, read the appropriate sections before making your selection.

[x] Life sciences   [ ] Behavioural & social sciences   [ ] Ecological, evolutionary & environmental sciences

For a reference copy of the document with all sections, see nature.com/documents/nr-reporting-summary-flat.pdf

Life sciences study design

All studies must disclose on these points even when the disclosure is negative.

Sample size	this is applicable for quantification of immunofluoresecene analysis, we chose to monitor at least 1000 cells
Data exclusions	no data exclusion
Replication	at least three independent experiments where performed for each of the assays presented in the manuscript
Randomization	no randomization
Blinding	Blinding was performed only for the automated fluoresence image analysis

Reporting for specific materials, systems and methods

We require information from authors about some types of materials, experimental systems and methods used in many studies. Here, indicate whether each material, system or method listed is relevant to your study. If you are not sure if a list item applies to your research, read the appropriate section before selecting a response.

Materials & experimental systems

n/a	Involved in the study	
☐	☒	Antibodies
☐	☒	Eukaryotic cell lines
☒	☐	Palaeontology and archaeology
☐	☒	Animals and other organisms
☒	☐	Clinical data
☒	☐	Dual use research of concern
☒	☐	Plants

Methods

n/a	Involved in the study	
☒	☐	ChIP-seq
☒	☐	Flow cytometry
☒	☐	MRI-based neuroimaging

Antibodies

Antibodies used

NAME COMPANY CATALOG number DILUTION source used

anti-CMG2 GENOVAC home made 500x rat WB
anti-MEK1 COVALAB home made 1000x rabbit WB
anti-CMG2 R&D AF2940 1000x goat IP
anti-CMG2 Proteintech 16723_1 1000x rabbit WB
anti-TEM8 R&D AF3886 1000x goat IP, WB
anti-ARF6 LSBio LS_C81881 2000x rabbit IP,WB
anti-TRAPalpha Santa Cruz ref: 100314 2000x rabbit WB
anti-CALNEXIN MILLIPORE MAB3126 2000x mouse WB
anti-TUBULIN SIGMA T5168 4000x mouse WB
anti-ACTIN MILLIPORE MAB1501 4000x mouse WB
anti-V5 Invitrogen 46-0705 2000x mouse WB
anti-GFP Takara 632592 3000x rabbit WB
anti-MYC SIGMA 9E10 M4439 2000x mouse WB
anti-ZDHHC3-GODZ ABCAM ab31837 200x rabbit ICC, WB
anti-GM130 BD clone35 610823 200x mouse ICC
anti-GAPDH Acris 4A1-MA0100 4000x mouse WB
anti-Talin SIGMA T3287 4000x mouse WB
anti-vinculin SIGMA V9131 4000x mouse WB
anti phospho Tyrosine MILLIPORE 05-1050 1000x mouse WB
anti-RHOA Cell signalling 67B9 2117 1000x rabbit WB
anti-SRC ABCAM 16885 2000x mouse WB
anti-PA List Biological Laboratories 771B 1000x rabbit WB
anti-MEK2 SANTA CRUZ 12578 1000x rabbit WB
anti-MEK3 SANTA CRUZ 960 1000x rabbit WB
anti-Collagen VI Fitzgerald Laboratories 70R-CR009X 1000x rabbit WB
anti-IgG SANTA CRUZ 2025 2000x mouse IP
anti-mouse HRP GEHealthcare AB_772210 NA931V 16991100 4000x sheep WB
anti-rabbit-HRP GEHealthcare AB_772206 NA934V 17090198 4000x donkey WB

Validation

Antibodies were validated using siRNA against the gene of interest

Eukaryotic cell lines

Policy information about cell lines and Sex and Gender in Research

Cell line source(s)

HeLa cells, ATCC CCL-2
RPE1 cells , ATCC CRL 4000

Authentication

Profiling of human cell line was done using highly-polymorphic short tandem repeat loci (STRs). STR loci were amplified using the PowerPlex® 16 HS System (Promega). Fragment analysis was done on an ABI3730xl (Life Technologies) and the resulting data were analyzed with GeneMarker HID software (Softgenetics).

Mycoplasma contamination

Cells were tested negative every 3 month by PCR, last PCR February 4th, 2026.

Commonly misidentified lines
(See ICLAC register)

No misidentified lines

Animals and other research organisms

Policy information about studies involving animals; ARRIVE guidelines recommended for reporting animal research, and Sex and Gender in Research

Laboratory animals	fish:All experiments involved genetically modified zebrafish (Danio rerio) embryos carrying the Tg(fli1:eGFP)y1 and Tg(gata1:DsRed) sd2 transgenes before 5 days post-fertilisation.<br>mouse: we used 84 mice from 11 week old C57BL/6-K18 mice
Wild animals	This study did not involve wild animals.
Reporting on sex	fish:All experiments were performed on embryos younger than 5 days post-fertilisation, a stage at which sex determination is not yet possible.<br>mouse : 25 male and 59 female in the cohort
Field-collected samples	This study did not involve samples collected from the field.
Ethics oversight	All experimental procedures were conducted in accordance with Swiss federal legislation on animal experimentation (Animal Welfare Act, TSchG; Animal Welfare Ordinance, TSchV), under authorization granted by the Federal Food Safety and Veterinary Office (FSVO) of the Canton of Vaud, Switzerland (authorization no. VD-H23 for fish, authorization no. VD-3794b for mice).

Note that full information on the approval of the study protocol must also be provided in the manuscript.

Plants

Seed stocks	*Report on the source of all seed stocks or other plant material used. If applicable, state the seed stock centre and catalogue number. If plant specimens were collected from the field, describe the collection location, date and sampling procedures.*
Novel plant genotypes	*Describe the methods by which all novel plant genotypes were produced. This includes those generated by transgenic approaches, gene editing, chemical/radiation-based mutagenesis and hybridization. For transgenic lines, describe the transformation method, the number of independent lines analyzed and the generation upon which experiments were performed. For gene-edited lines, describe the editor used, the endogenous sequence targeted for editing, the targeting guide RNA sequence (if applicable) and how the editor was applied.*
Authentication	*Describe any authentication procedures for each seed stock used or novel genotype generated. Describe any experiments used to assess the effect of a mutation and, where applicable, how potential secondary effects (e.g. second site T-DNA insertions, mosiacism, off-target gene editing) were examined.*
