# Supplementary material for: Dynamic S-acylation controls CMG2 maturation extracellular matrix regulation and anthrax toxin susceptibility in vivo
Source: Nat Commun. 2026 Jul 6;17:8364. doi: 10.1038/s41467-026-75028-2 (PMC13473502; doi:10.1038/s41467-026-75028-2)
Supplement: Supplementary file 2 [file 41467_2026_75028_MOESM12_ESM.pdf]

nature portfolio

Peer Review File

Dynamic S-acylation controls CMG2 maturation extracellular matrix regulation and anthrax toxin susceptibility in vivo

Corresponding Author: Professor Françoise van der Goot

This file contains all reviewer reports in order by version, followed by all author rebuttals in order by version.

Version 0:

Reviewer comments:

Reviewer #1

(Remarks to the Author)
This study reveals that the anthrax toxin receptor CMG2 is regulated by sequential S-acylation cycles controlling its entire life cycle. In the endoplasmic reticulum, ZDHHC7-mediated acylation of juxtamembrane cysteines protects newly synthesized CMG2 from degradation, increasing folding efficiency five-fold. The thioesterase APT2 counteracts this by removing acyl chains, targeting folding intermediates for ER-associated degradation. In the Golgi, ZDHHC3 acylates a distal cysteine, enabling Arf6-dependent transport to the plasma membrane. Upon ligand binding, membrane-anchored APT2 is recruited to CMG2, triggering deacylation that releases the receptor from cytoskeletal anchors and permits endocytosis. Inhibiting APT2 blocks anthrax toxin entry and CMG2-mediated collagen VI degradation. These findings establish APT2 as a druggable target for Hyaline Fibromatosis Syndrome (by increasing CMG2 expression) and anthrax intoxication (by blocking toxin internalization). The sequential acylation-deacylation model is well supported, and the translational implications are exciting. With revisions to improve clarity, address mechanistic gaps, and strengthen data presentation, the manuscript would be suitable for publication.

Specific comments
1. The introduction provides good background but is slightly lengthy. Some paragraphs (e.g., lines 37–45) could be condensed to focus more directly on the knowledge gap and the study's objectives.
2. Figure 1d: The decreased total CMG2 protein may contribute to the observed decrease in S-acylation. The authors should check the S-acylation levels of CMG2 protein and the mutants using IP-ABE.
3. Figure 2: The siRNA screen is robust. However, the authors should clarify whether off-target effects were ruled out (e.g., by rescue experiments with siRNA-resistant ZDHHC3/7).
4. Figure 3: the claim that APT2 acts in the ER (lines 215–216) is inferred but not directly demonstrated. Consider using subcellular fractionation or ER-specific markers to confirm the localization of APT2 activity.
5. Figure 4: The Golgi retention phenotype upon ZDHHC3 silencing is compelling. However, the interaction between CMG2 and Arf6 should be quantified (e.g., by co-IP densitometry) to strengthen the claim.
6. Figure 5: the use of Bafilomycin A to exclude lysosomal degradation is appropriate, but the authors should also rule out proteasomal degradation (e.g., using MG132) for completeness.
7. Figure 6: the claim that APT2 must be S-acylated to bind CMG2 (Fig. 6e–f) is based on a single mutant (C2S). A complementary approach (e.g., membrane binding assays) would strengthen this conclusion.
8. Figure 7: the MEK2 cleavage assay in APT2 KO cells (Fig. 7a–b) shows variability in baseline MEK2 levels. The authors should normalize to total protein or use a different readout (e.g., LF activity assay) to confirm the effect.
Minor comments
9. Line 218: "five-fold difference" – is this statistically significant? Include p-value.
10. Line 496: "Treatment of an anthrax infection" – consider rephrasing to "post-exposure prophylaxis" for clarity.

Reviewer #2

(Remarks to the Author)
Summary of findings
This study identifies temporally and spatially ordered S-acylation/deacylation cycles as a regulatory mechanism controlling CMG2 folding, trafficking, and signalling. The demonstration that ER-localized ZDHHC7-mediated acylation protects folding intermediates from ERAD, while Golgi-localized ZDHHC3 acylation enables plasma membrane delivery, provides a

compelling mechanistic framework linking lipidation to receptor biogenesis. The identification of APT2 as a dynamic regulator of both CMG2 maturation and ligand-induced signalling is particularly impactful, with clear implications for both anthrax intoxication and Hyaline Fibromatosis Syndrome.
The study is an elegant, satisfying, and comprehensive mechanistic dissection of the role of acylation and deacylation in CMG2 biogenesis, trafficking and signalling, and will be a valuable contribution to the field. The work will be of significant interest to the fields of protein S-acylation, membrane protein biogenesis, and receptor trafficking and signalling. While S-acylation has been widely implicated in regulating membrane association, trafficking, and signalling of mature proteins, its role as a dynamic regulator of early biogenesis and folding efficiency in the ER is not as well established.
The identification of temporally ordered, compartment-specific S-acylation-deacylation cycles that govern CMG2 folding, ER exit, Golgi trafficking, and ligand-induced signalling represents a conceptual advance beyond prior descriptive studies of receptor palmitoylation. In particular, the demonstration that ZDHHC7-mediated acylation protects folding intermediates from ERAD, and that APT2 counteracts this process during biogenesis, builds on the functional scope of S-acylation in the domain of protein quality control.
The work also connects acylation dynamics to disease relevant contexts, including Hyaline Fibromatosis Syndrome and anthrax toxin uptake, and identifies APT2 as a pharmacologically tractable node (though global inhibition of such a key enzyme is a practical matter not discussed). Compared with earlier studies that established CMG2 and TEM8 as palmitoylated receptors, this study provides a mechanistic and temporal framework that integrates lipidation with folding, trafficking, and signalling.

The methodology is generally sound and well executed, with appropriate controls throughout. A small number of clarifications (for example, explicit normalization strategies for certain quantitative analyses and clearer annotation of figure legends) would further improve reproducibility and accessibility, but these are minor and readily addressable during revision.

General comments
Fig 3 – If zDHHC7 acylates CMG2 early in its lifetime, you would predict siRNA zDHHC7 only would be sufficient to recapitulate the phenotype of AAC mutation? Similarly, should zDHHC3 siRNA match behaviour of CCA?

Discussion – recruitment of APT2 being dependent on the active site is unusual and implies that catalysis (deacylation) is much slower than recruitment for the two proteins to co-immunoprecipitate together. Can the authors discuss this in the context of what is already known about how APT2 recognises and interacts with substrates?

Minor queries about the content of the text
Line 62:
The phrase ‘rendering the protein vulnerable to genetic mutations’ could be misread as implying increased mutagenesis. Clarifying that inefficient folding reduces tolerance to missense mutations (i.e., increases susceptibility to mutation-induced misfolding and ERAD) would improve precision.

Line 85:
Given that occupancy is never measured per site and heterogeneity is likely, “fully” is a bit strong wording?

Lines 108-110:
The authors say "The AAC variant showed the strongest reduction, indicating that acylation of these two juxtamembrane cysteines influences acylation of the third site." How do they rule out that the third site just has lower intrinsic occupancy, or faster turnover, rather than being “influenced” by the first two? While the reduced 3H-palmitate incorporation in the AAC mutant is consistent with sequential S-acylation, the current data cannot distinguish whether acylation of Cys344/345 is required for subsequent modification of Cys379 versus differences in intrinsic occupancy or turnover of the distal site. Clarifying this interpretation in the text would improve precision.

Line 110:
It is not immediately clear how the authors have come up with the suggestion that kinetic labelling experiments demonstrate later labelling of Cys379. Eventually I worked out that this comes from comparing WT with CCA in Fig 1e, but I suggest adding this explanation to the text.

Lines 155 & 156:
Wild type CFTR is also very inefficiently folded. See J Biol Chem 269 (41) 25710-25718 and EMBO J 13, 6076–6086, (1994).

Lines 169-170:
The reduced degradation observed with longer pulse duration reflects enrichment of stabilised molecules rather than altered degradation kinetics, and this interpretation could be clarified in the text.

Line 173:
Incomplete sentence. Possibly related – no reference to Fig 3d in the text?

Line 409-411:
Needs editing. Appears to contain a comma splice/run-on and ambiguous referent (differences between what? WT vs KO? replicates? conditions?). Could be clarified to better distinguish the experimental observation from the analytical limitation.

Line 415/6:
Does Fig 7e really show oligomerisation of PA?

Queries & Comments about the Figures
Figure 1
Fig 1a legend - I don't see citation for this structure - AF, PDB? Should the third cysteine be labelled as Cys379 not 478 according to the text? Better colour contrast will improve clarity.
Fig 1c - I don't see definition of PNS abbreviation in the text or the legend.
Fig 1d / 1e appears to be confounded by differences in amount of each mutant captured (according to the western blot in 1d). Did the authors normalise incorporation of 3H to CMG2 capture? The same applies to other figures – for example 2c.
Fig 1e – Suggest need a different colour for CCA and CAA for clarity (both currently grey).
Fig 1e – can the authors present also present a quantitative (statistical) comparison of WT and CCA?
Fig 1e – The graph doesn't seem to perfectly represent the 3H band intensities in panel d? Especially CCA vs CAC?
Fig 1 legend – Typo - second “panel d” should be “e”

Figure 2
It is confusing that the cell type changes during this figure. Can the authors label each panel to indicate which cells are being investigated. Also, perhaps explain why?
Fig 2a – Since acylation is found to stabilise CMG2 is it surprising that CMG2 levels don’t change when a zDHHC is silenced? This same comment applies to several other panels showing siRNA and total cell extract / CMG2 expression (2c, 2d, 4b).
Fig 2c – description of the experiment hard to follow. I initially expected to find that cycloheximide blocked all acylation. Why is that not the case? Is the zDHHC3-mediated acylation in the presence of cycloheximide of protein that was already synthesised when the cycloheximide was applied? Suggest you state this in the text.
Fig 2d - Error/edit - Labels are misaligned with lanes
Fig 2e – it is confusing that the graph and the gel are presented in a different order.
Fig 2f – resolution is poor and I cannot read. Font too small and colour is too low contrast.

Figure 3
Fig 3b – error, missing what it’s normalised to “% of …” on the y axis
Fig 3b (and other pulse chase experiments) – are statistical comparisons possible? Can the authors plot the half life of each mutant?

Figure 4
Fig 4a - It seems these siRNA treated cells are showing altered GM130 distribution? I recommend the authors put the high throughput imaging (Extended Data Figure 3) in the main figure, but perhaps plot this data differently (violin plots when n>2500?).
Fig 4b - Can the authors blot surface membrane preps for a integral membrane housekeeper to show the siRNA-mediated changes in delivery are specific for CMG2 rather than a generalised effect?
Fig 4c – the legend does not explain the second graph in this panel (CHC siRNA).

Figure 5
Fig 5b – RhoA appears to be is recruited to mutant AAC, while this mutant is also still in complex with TVA? I didn’t see the authors address this in the text. There's an issue with a blue box or something around a blot in this panel.
Fig 5bc – the mean data for actin binding of CCA (5c) don’t match the western blot presented in Fig 5b (5b appears to show no binding in the presence of PA63, 5c shows a modest decrease).
Fig 5c - Is there a corresponding blot for the siRNA data in the figures? Mention in legend?

Figure 7
All the phenotypic screens rely on pharmacological or genetic inhibition of APT2, which has numerous substrates. Can you ascribe the effect you measure solely to CMG2?
Fig 7c legend – ML349 in ug/ml or uM?
Fig 7f is not referred to in body of text
End of Fig 7 legend is trimmed / missing.

Extended Figure 1
1d – The legend says CMG2 but the graph is titled TEM8. The description of this panel includes results / conclusions – perhaps it could be rewritten to match the other parts of the legend. Recommend revise ‘dual silencing’ (legend) as this implies both were silenced in the same sample – perhaps ‘silencing either zDHHC3 or zDHHC7’.

Extended Figure 5
5a – missing ‘PA63’ label for the - / + above each lane

Extended Figure 6
6b - Error/edit - need to fix label alignment with lanes here
6d – Why is the PA pore detected in the APT2 KO cells? Is the expected result not that APT2 loss means we don’t see PApore, like in panel b?

Typographical errors
Line 47-48 – Fix punctuation

Line 72 – “recruited” typo? Mean required?

Line 72-73 - Standardise spelling - in one sentence signalling is American spelling, in the next it's British spelling.

Line 100 – reference should be a number not author-year?

Lines 150 & 158 – t (1/2) should all be subscript

Line 214 – newly synthesised

Line 403 - Typo - Seems to be missing an "and" or "then".. it's a bit awkward as is.

Line 416 – Typo, “consistent” not “consistently”

Line 508 – Typo “50to 70%”

Reviewer #3

(Remarks to the Author)
I co-reviewed this manuscript with one of the reviewers who provided the listed reports. This is part of the Nature Communications initiative to facilitate training in peer review and to provide appropriate recognition for Early Career Researchers who co-review manuscripts.

Reviewer #4

(Remarks to the Author)
CMG2 and its related protein TEM8 are the two anthrax toxin receptors present on the surface of target cells. Mutations in these proteins have been identified in human patients and are associated with abnormal extracellular matrix protein deposition, highlighting their physiological roles in ECM turnover. This group previously showed that CMG2 functions as a receptor for collagen VI in mice.
In this manuscript, Abrami et al. describe post translational palmitoylation on three cysteine residues within the CMG2 cytosolic tail and explore how these modifications influence CMG2 biogenesis, trafficking, cell surface expression, and its functions as both an anthrax toxin receptor and a collagen VI receptor. The authors provide a reasonable set of data supporting their conclusions. However, I have several concerns that should be addressed:
1. Cytosolic tail–truncated versions of CMG2 and TEM8 (which therefore lack palmitoylation sites) have been reported to remain functional as anthrax toxin receptors. How do the authors reconcile their findings with these earlier observations?
2. The study relies heavily on siRNA knockdown of ZDHHC3 and ZDHHC7 to assess their roles in CMG2 palmitoylation, yet no data are provided to demonstrate the extent of knockdown for these enzymes.
3. The authors used the pan thioesterase inhibitor Palmostatin B (more selective for APT1) in Fig. 3J and ED Fig. 2d. Why was the more APT2 specific inhibitor ML349 not used in these experiments?
4. Fig. 7c: The LF mediated cleavage of MEK2 is difficult to evaluate without information on the specificity of the MEK2 N antibody. MEK3 cleavage is typically easier to detect and could strengthen the analysis. Although the cell line used may not be highly sensitive to LF, the authors could test LF DT or LF ETA fusion to determine whether the APT2 inhibitor ML349 protects cells from toxin induced effects. The APT2 KO cells shown in Fig. 7a could also be assessed for toxin susceptibility.
5. ED Fig. 6b–d: LF mediated cleavage of MEK1 is difficult to discern. In ED Fig. 6d, the cleavage pattern appears similar between APT2 KO and APT2 KO + APT2 rescue conditions.
6. Because APT2 appears to have opposing roles, negative effect on early CMG2 biogenesis but a positive role in ligand induced internalization, it is unclear how APT2 could be considered a viable therapeutic target for anthrax, as suggested by the authors.
Minor comments:
1. Words appear to be missing at the end of line 173.
2. The titles in Fig. 4c (“Ctrl,” “siRNA CHC”) are confusing and should be clarified.
3. Fig. 2f and Fig. 7g are low in quality and difficult to read.

Version 1:

Reviewer comments:

Reviewer #1

(Remarks to the Author)
The revision satisfies me. I have no further questions.

Reviewer #4

(Remarks to the Author)
The authors have made substantial efforts to address this reviewer's concerns as well as those raised by the other reviewers. I am satisfied with the current revision.

I only have the following minor points that the authors may consider discussing or commenting on in the final version:
1. The legends for Fig. 1f appear to be missing.
2. In Fig. 2, cycloheximide treatment does not seem to affect total CMG2 protein levels. Please provide an explanation for this observation.
3. CMG2 ligand binding triggers TVA release as well as the recruitment of RhoA, β arrestin, and mDia. While TVA release is clearly critical for CMG2 internalization, please comment on whether the recruitment of RhoA, β arrestin, and mDia is functionally important for CMG2 receptor activity.

Open Access This Peer Review File is licensed under a Creative Commons Attribution 4.0 International License, which permits use, sharing, adaptation, distribution and reproduction in any medium or format, as long as you give appropriate credit to the original author(s) and the source, provide a link to the Creative Commons license, and indicate if changes were made.
In cases where reviewers are anonymous, credit should be given to 'Anonymous Referee' and the source.
The images or other third party material in this Peer Review File are included in the article's Creative Commons license, unless indicated otherwise in a credit line to the material. If material is not included in the article's Creative Commons license and your intended use is not permitted by statutory regulation or exceeds the permitted use, you will need to obtain permission directly from the copyright holder.
To view a copy of this license, visit https://creativecommons.org/licenses/by/4.0/

Point-by-point reply to the reviewers

We thank all reviewers for their constructive comments, which have helped us improve the rigour and clarity of the manuscript and enhance its overall impact, in particular through the addition of in vivo experiments in zebrafish demonstrating that inhibition of deacylation protects against anthrax lethal toxin-induced effects.

Reply to Reviewer #1 :
We thank the reviewer for the constructive comments and suggestions, which have helped improve the manuscript.

1. The introduction provides good background but is slightly lengthy. Some paragraphs (e.g., lines 37–45) could be condensed to focus more directly on the knowledge gap and the study's objectives.
The introduction has been condensed, as to focus on the knowledge gap.

2. Figure 1d: The decreased total CMG2 protein may contribute to the observed decrease in S-acylation. The authors should check the S-acylation levels of CMG2 protein and the mutants using IP-ABE
We now provide a quantification of the CMG2 expression levels, WT and mutants (Extended Data 1). The observed decrease in acylation for certain mutants clearly contribute, but cannot fully explain the decrease in acylation as now clairified in the text.
To analyze S-acylation levels, or site occupancy, we have now performed PEGylation, an ABE derived method, of WT and mutant CMG2. This analysis shows that the entire WT CMG2 population undergoes S-acylation mostly on all three cysteines (Fig. 1f).

3. Figure 2: The siRNA screen is robust. However, the authors should clarify whether off-target effects were ruled out (e.g., by rescue experiments with siRNA-resistant ZDHHC3/7).
We now show that ectopic expression of ZDHHC3 or 7 restores CMG2 S-acylation after silencing of the endogenous corresponding enzyme (Extended Fig. 2d).

4. Figure 3: the claim that APT2 acts in the ER (lines 215–216) is inferred but not directly demonstrated. Consider using subcellular fractionation or ER-specific markers to confirm the localization of APT2 activity.
We thank the reviewer for raising this important point. To directly assess whether APT2 can access CMG2 in the ER, we examined its interaction with the ER retained the L45P CMG2 mutant. We found that APT2 efficiently co-immunoprecipitates with CMG2 L45P. We extended this analysis to three additional HFS-associated folding mutants (G105D, I189T and L349R) previously shown to be retained in the ER (Deuquet et al., 2009), and also observed co-immunoprecipitation with APT2. In contrast, under the same conditions, no interaction between APT2 and WT CMG2 was detected.
These experiments are now shown in Extended Fig. 3f.

5. Figure 4: The Golgi retention phenotype upon ZDHHC3 silencing is compelling. However, the interaction between CMG2 and Arf6 should be quantified (e.g., by co-IP densitometry) to strengthen the claim.

We apologize for the unclear labelling of the right panel of Fig. 4f (now Fig. 4g), which actually showed the quantification of the IP. We have now clarified the label of the y axis.

6. Figure 5: the use of Bafilomycin A to exclude lysosomal degradation is appropriate, but the authors should also rule out proteasomal degradation (e.g., using MG132) for completeness.
We have now repeated the experiments in which we monitor the effect of PA treatment on the levels of CMG2 palmitoylation with and without MG132. As observed upon Bafilomycin A treatment, MG132 had no effect, further strengthening the conclusion that PA treatment leads to CMG2 deacylation (new Fig. 5e, Extended Fig. 4c).

7. Figure 6: the claim that APT2 must be S-acylated to bind CMG2 (Fig. 6e–f) is based on a single mutant (C2S). A complementary approach (e.g., membrane binding assays) would strengthen this conclusion.
We have now strengthened the conclusion that APT2 must associate with membranes in order to bind CMG2 by extending the analysis to two additional previously characterized APT2 mutants: $APT2^{PosPatch}$, a quintuple mutant lacking the basic surface patch required for membrane interaction, and APT2 M68E, a mutant of the hydrophobic membrane inserting loop. Both mutants these fail to bind liposomes in vitro, and are predominantly cytosolic in vivo (*Nature Chemical Biology* 2021, Fig. 3c and g). We now show in Extended Fig. 5d that these two membrane-binding defective mutants also fail to be recruited to CMG2 upon ligand binding.

8. Figure 7: the MEK2 cleavage assay in APT2 KO cells (Fig. 7a–b) shows variability in baseline MEK2 levels. The authors should normalize to total protein or use a different readout (e.g., LF activity assay) to confirm the effect.
We have repeated the experiments monitoring lethal factor activity by immunofluorescence. To avoid clonal effects, we compared APT2 KO cells reconstituted with either WT APT2 or the acylation-deficient C2S mutant, which is inactive because it cannot be interact stably with membranes. Following a suggestion from Reviewer 3, we monitored full-length MEK3 using an antibody against its N-terminus, which is cleaved by LF. MEK3 detection indeed provided a robust readout (Fig. 7a,b). These results confirm that LF-mediated cleavage of MEK3 is impaired in cells lacking active APT2.

Minor comments
9. Line 218: "five-fold difference" – is this statistically significant? Include p-value.
The 5 fold difference is significant with p=0.001 as now mentionned in the text. We also added a historgram to illustrate this in Extended Fig. 3f.

10. Line 496: "Treatment of an anthrax infection" – consider rephrasing to "post-exposure prophylaxis" for clarity.
We have included this suggestion.

Reply to Reviewer #2
We thank the reviewer for the pertinent summary of our work and are pleased that it was well received. We highly appreciate the thoughtful questions and detailed

comments on the text and figures, which helped improve the clarity and rigor of the manuscript.

1. Fig 3 – If zDHHC7 acylates CMG2 early in its lifetime, you would predict siRNA zDHHC7 only would be sufficient to recapitulate the phenotype of AAC mutation? Similarly, should zDHHC3 siRNA match behaviour of CCA?

Conceptually, the AAC mutant and ZDHHC7 knockdown only partially mimic one another. In the ER, ZDHHC7 modifies the two juxtamembrane cysteines. However, when ZDHHC7 is depleted, the subset of CMG2 molecules that successfully fold and exit the ER can subsequently encounter ZDHHC3, which we believe is likely capable of modifying these cysteines. Thus, the absence of ZDHHC7 does not fully phenocopy the AAC mutant, in which the juxtamembrane cysteines are permanently unavailable for acylation. In other words, we do not think that ZDHHC3 is intrinsically unable to modify these cysteines, but this does not represent the physiological sequence of events.

With respect to ZDHHC3, the effects of its knockdown on CMG2 more closely resembles the CCA mutant, as both specifically affect modification of the distal cysteine required for post-Golgi trafficking. The *mathcing in behavior* is now underlined in the text.

2. Discussion – recruitment of APT2 being dependent on the active site is unusual and implies that catalysis (deacylation) is much slower than recruitment for the two proteins to co-immunoprecipitate together. Can the authors discuss this in the context of what is already known about how APT2 recognises and interacts with substrates?

This is again a very insightful comment. The recruitment of APT2 does not require catalytic activity, and we have taken care to clarify this point in the revised manuscript. Indeed, the catalytically inactive APT2 S121A mutant is efficiently recruited to CMG2, demonstrating that recruitment per se is not dependent on an active catalytic site.

However, as the reviewer correctly notes, co-immunoprecipitation of APT2 with CMG2 is only observed when CMG2 is acylated. Our interpretation is that the interaction becomes experimentally detectable only when a CMG2-bound acyl chain engages the hydrophobic acyl chain-binding pocket of APT2. This indeed implies that deacylation is sufficiently slow relative to hydrolysis and separation between the enzyme and the substrate to allow co-immunoprecipitation.

This interpretation is consistent with existing structural and biochemical data on APT2, which show that deacylation involves insertion of the acyl chain into a defined hydrophobic pocket that is spatially linked, but not identical, to the catalytic site. To further support this model, we now show in Extended Fig. 5ef that ML349, which occupies this hydrophobic acyl chain-binding pocket, abolishes APT2-CMG2 co-immunoprecipitation, despite the fact that ML349 does not prevent APT2 membrane association in vivo (Extended Fig. 5g) and in vitro (Extended Fig. 5h). This provides independent pharmacological evidence that engagement of a CMG2 acyl chain by APT2 is required for stable complex detection.

Minor queries about the content of the text

Line 62:

The phrase 'rendering the protein vulnerable to genetic mutations' could be misread as implying increased mutagenesis. Clarifying that inefficient folding reduces

tolerance to missense mutations (i.e., increases susceptibility to mutation-induced misfolding and ERAD) would improve precision.
Thank you for the suggestion, which we have included

Line 85:
Given that occupancy is never measured per site and heterogeneity is likely, "fully" is a bit strong wording?
We agree that heterogeneity is likely, and we have removed the word "fully".
To probe occupancy/heterogeneity, we have now performed PEG-maleimide labeling of CMG2. To improve resolution, samples were treated with N-glycosidase to remove heterogeneous N-linked glycans, which otherwise obscure discrete mobility shift on CMG2. This analysis indicates that essentially the entire WT CMG2 pool has at least 2 acyl chains, with the majority modified on three cysteines (New Fig. 1f). We cannot exclude that PEGylation is complete, but this could explain the +2PEG band.

Lines 108-110:
The authors say "The AAC variant showed the strongest reduction, indicating that acylation of these two juxtamembrane cysteines influences acylation of the third site." How do they rule out that the third site just has lower intrinsic occupancy, or faster turnover, rather than being "influenced" by the first two? While the reduced 3H-palmitate incorporation in the AAC mutant is consistent with sequential S-acylation, the current data cannot distinguish whether acylation of Cys344/345 is required for subsequent modification of Cys379 versus differences in intrinsic occupancy or turnover of the distal site. Clarifying this interpretation in the text would improve precision.
We agree and have adapted the text accordingly.

Line 110:
It is not immediately clear how the authors have come up with the suggestion that kinetic labelling experiments demonstrate later labelling of Cys379. Eventually I worked out that this comes from comparing WT with CCA in Fig 1e, but I suggest adding this explanation to the text.
This has now been clarified.

Lines 155 & 156:
Wild type CFTR is also very inefficiently folded. See J Biol Chem 269 (41) 25710-25718 and EMBO J 13, 6076–6086, (1994).
Thank you for this important reference, we have now added.

Lines 169-170:
The reduced degradation observed with longer pulse duration reflects enrichment of stabilised molecules rather than altered degradation kinetics, and this interpretation could be clarified in the text.
We thank the reviewer for this clear wording which we have now used in the manuscript.

Line 173:
Incomplete sentence. Possibly related – no reference to Fig 3d in the text?
The end of this paragraph was inadvertently deleted during editing and has now

been fully restored. In doing so, we have also reinstated the reference to Fig. 3d in the appropriate place in the text.

Line 409-411:
Needs editing. Appears to contain a comma splice/run-on and ambiguous referent (differences between what? WT vs KO? replicates? conditions?). Could be clarified to better distinguish the experimental observation from the analytical limitation.
These sentences have been removed.

Line 415/6:
Does Fig 7e really show oligomerisation of PA?
We have corrected the panel numbering, indeed 7e did not show oligomerization of PA.

Queries & Comments about the Figures
Figure 1
Fig 1a legend - I don't see citation for this structure - AF, PDB? Should the third cysteine be labelled as Cys379 not 478 according to the text? Better colour contrast will improve clarity.
We thank the reviewer for spotting this. It is indeed Cys-479, as now corrected on the figure and in the text. We have improved the figure and the legend.

Fig 1c - I don't see definition of PNS abbreviation in the text or the legend.
The definition of the PNS (Post Nuclear Supernatant) is now provided in the legend.

Fig 1d / 1e appears to be confounded by differences in amount of each mutant captured (according to the western blot in 1d). Did the authors normalise incorporation of 3H to CMG2 capture? The same applies to other figures – for example 2c
We agree with this point. To address the potential confounding effect of differences in CMG2 expression between mutants, we have now added a quantification of the expression levels of each CMG2 mutant and their corresponding acylation levels in Extended Data Fig. 1. This analysis confirms that the differences in [^{3}H]-palmitate incorporation reported in Fig. 1d cannot be explained solely by differences in protein abundance.
Regarding Fig. 2c, the Western blot shown corresponds to the immunoprecipitated material rather than total cell extracts, in contrast to Fig. 1d. Because the immunoprecipitations yielded very similar amounts of CMG2 across conditions, we did not further normalize the [^{3}H]-palmitate signal to the Western blot. In this context, quantification of incorporated radioactivity using the Typhoon imager provides a more reliable and sensitive readout than densitometric quantification of immunoblot signals, which are inherently less linear.

Fig 1e – Suggest need a different colour for CCA and CAA for clarity (both currently grey).
The color of the CAA mutant has been changed to differentiate it from CCA.

Fig 1e – can the authors present also present a quantitative (statistical) comparison of WT and CCA?

We have now indicated on the graph that the difference between the WT and CCA signals was significantly different only for at the three latest time points.

Fig 1e – The graph doesn't seem to perfectly represent the 3H band intensities in panel d? Especially CCA vs CAC?
We agree that the correspondence between the single time-point autoradiography shown in Fig. 1d and the kinetic quantification in Fig. 1e is not perfect, particularly for the CCA versus CAC mutants. We do not believe this reflects a systematic error, but rather highlights the intrinsic variability of single time-point measurements when comparing closely related mutants.
In our experience, kinetic [^{3}H]-palmitate incorporation assays are considerably more robust and reproducible than single end-point measurements, as they integrate labeling over time and are less sensitive to small differences in expression, recovery during immunoprecipitation, or exposure conditions. Indeed, the kinetic profiles shown in Fig. 1e have been highly reproducible across independent experiments, whereas comparisons based on individual time points can show greater variability. Importantly, our conclusions regarding sequential acylation and the dominant contribution of the juxtamembrane cysteines are based on the kinetic analyses rather than on any single end-point comparison, and are further supported by independent experiments throughout the manuscript.

Fig 1 legend – Typo - second "panel d" should be "e"
This has been corrected.

Figure 2
It is confusing that the cell type changes during this figure. Can the authors label each panel to indicate which cells are being investigated. Also, perhaps explain why?
We have now clarified that RPE1 allow the analysis of endogenous CMG2, whereas Hela cells were used for transient transfection of V5-tagge WT and mutant CMG2. The figure panels now mention the cell lines.

Fig 2a – Since acylation is found to stabilise CMG2 is it surprising that CMG2 levels don't change when a zDHHC is silenced? This same comment applies to several other panels showing siRNA and total cell extract / CMG2 expression (2c, 2d, 4b).
When analyzing total cell extracts, we do observe decreases in CMG2 levels upon ZDHHC silencing, even after transient transfection. These differences are not readily visible in the immunoprecipitation experiments shown in Fig. 2a, which enrich for CMG2. The same applies to Figs. 2c, 2d, and 4b.

Fig 2c – description of the experiment hard to follow. I initially expected to find that cycloheximide blocked all acylation. Why is that not the case? Is the zDHHC3-mediated acylation in the presence of cycloheximide of protein that was already synthesised when the cycloheximide was applied? Suggest you state this in the text.
We agree with the interpretation of the reviewer, which we have now made more explicit in the text. The residual acylation observed in the presence of cycloheximide indeed reflects modification of CMG2 molecules that had already been synthesized prior to cycloheximide treatment and had reached the Golgi.

Fig 2d - Error/edit - Labels are misaligned with lanes
This has been corrected.

Fig 2e – it is confusing that the graph and the gel are presented in a different order.
The order of the graph has been adapted to that of the gel.

Fig 2f – resolution is poor and I cannot read. Font too small and colour is too low contrast.
The readability of the cartoon has been improved.

Figure 3
Fig 3b – error, missing what it's normalised to "% of …" on the y axis
We have now specified in the legend that each condition was normalized to the same condition at t=0.

Fig 3b (and other pulse chase experiments) – are statistical comparisons possible? Can the authors plot the half life of each mutant?
We have now calculated statistical differences between conditions. As stated in the figure legend, AAA, ACC, CAC, and CAA are statistically different from WT at all time points during the chase ($p < 0.05$). CCA is statistically different from AAA at all time points, and statistically different from WT at and beyond the 5 h time point. We do not wish to place emphasis on absolute half-life values of the different mutants and therefore chose not to include them in the main manuscript. This is because the apparent half-life depends on the pulse duration and thus reflects folding, acylation, and degradation kinetics rather than a single intrinsic parameter. We have nevertheless added a summary table in Extended Data Fig. 3b reporting approximate apparent half-lives for endogenous CMG2, ectopically expressed WT and AAA CMG2, and the effect of Palmostatin B treatment.

Figure 4
Fig 4a - It seems these siRNA treated cells are showing altered GM130 distribution?
We agree with the revieweer that silencing ZDHHC3 alters the GM130 staining somewhat. We have not studied the consequences in depth.

I recommend the authors put the high throughput imaging (Extended Data Figure 3) in the main figure, but perhaps plot this data differently (violin plots when n>2500?).
We have moved the high throughput imaging panel to the main figures and now show a violin plot.

Fig 4b - Can the authors blot surface membrane preps for a integral membrane housekeeper to show the siRNA-mediated changes in delivery are specific for CMG2 rather than a generalised effect?
The effect of ZDHHC3 silencing on CMG2 trafficking is phenocopied by mutation of the ZDHHC3-targeted cysteine in CMG2. However we do not wish to claim that ZDHHC3 silencing specifically affects CMG2. It likely affects other ZDHHC3 targets. This is now explicitly mentionned in the text.

Fig 4c – the legend does not explain the second graph in this panel (CHC siRNA).
The legend now describes the silencing of clathrin heavy chain (CHC).

Figure 5

Fig 5b – RhoA appears to be is recruited to mutant AAC, while this mutant is also still in complex with TVA? I didn't see the authors address this in the text. There's an issue with a blue box or something around a blot in this panel.

Our interpretation is that these immunoprecipitations report on the entire population of CMG2 molecules. In the case of the AAC mutant, the population appears heterogeneous: most of CMG2 remains associated with the Talin-Vinculin-Actin complex, while a smaller fraction is capable of recruiting RhoA. This is consistent with a partial or inefficient signaling switch rather than a fully blocked transition. This now mentionned in the text.

The blue box has been removed.

Fig 5bc – the mean data for actin binding of CCA (5c) don't match the western blot presented in Fig 5b (5b appears to show no binding in the presence of PA63, 5c shows a modest decrease).

We fully agree, this is why we had mentionned in the text that for this particular mutant we observed significant experiment-to-experiment variability.

Fig 5c - Is there a corresponding blot for the siRNA data in the figures? Mention in legend?

We have corrected the legend to indicate that for the siRNA experiments the figure only shows the quantifications.

Figure 7

All the phenotypic screens rely on pharmacological or genetic inhibition of APT2, which has numerous substrates. Can you ascribe the effect you measure solely to CMG2?

We have modified the conclusion sentence of Figure 7 referering to the "activity of APT2" rather than the deacylation of CMG2 to address the reviewers concern.

Fig 7c legend – ML349 in ug/ml or uM?

The concentration of the drugs, which is indeed 10 μM, was removed and is mentionned in the Methods for all experiments.

Fig 7f is not referred to in body of text

The correct reference to the figure has been added.

End of Fig 7 legend is trimmed / missing.

We apolize for the this, the text box was previously too small for the full legend.

Extended Figure 1

1d – The legend says CMG2 but the graph is titled TEM8. The description of this panel includes results / conclusions – perhaps it could be rewritten to match the other parts of the legend. Recommend revise 'dual silencing' (legend) as this implies both were silenced in the same sample – perhaps 'silencing either zDHHC3 or zDHHC7'

These corrections have been made.

Extended Figure 5

5a – missing ‘PA63’ label for the - / + above each lane
PA63 has been added.

Extended Figure 6
6b - Error/edit - need to fix label alignment with lanes here
The lanes have now been better aligned.

6d – Why is the PA pore detected in the APT2 KO cells? Is the expected result not that APT2 loss means we don’t see PApore, like in panel b?
We thank this reviewer and reviewer 4 for drawing attention to this point. Upon re-examination, we realized that the blot in Extended Data Fig. 6d was mislabeled, Ctrl RPE1 cells are shown next to APT2 KO +APT2. The figure has now been corrected.

Typographical errors
All the typographical errors were corrected. We thank the reviewers for picking them up.

Line 47-48 – Fix punctuation
Line 72 – “recruited” typo? Mean required?
Line 72-73 - Standardise spelling - in one sentence signalling is American spelling, in the next it's British spelling.
Line 100 – reference should be a number not author-year?
Lines 150 & 158 – t (1/2) should all be subscript
Line 214 – newly synthesised
Line 403 - Typo - Seems to be missing an "and" or "then".. it's a bit awkward as is.
Line 416 – Typo, “consistent” not “consistently”
Line 508 – Typo “50to 70%”

Reply to Reviewer #4:
We thank the reviewer or their useful comments and for creating the opportunity of testing APT2 inhibitors in a more physiological context.

1. Cytosolic tail–truncated versions of CMG2 and TEM8 (which therefore lack palmitoylation sites) have been reported to remain functional as anthrax toxin receptors. How do the authors reconcile their findings with these earlier observations?
Liu and Leppla (J. Biol. Chem., 2003) indeed showed that a TEM8 truncation mutant containing only the extracellular and transmembrane domains can function as an anthrax toxin receptor. Their conclusion was that “the TEM8 extracellular region and transmembrane domain together constitute the minimum PA receptor structure.” We do not view this as being in conflict with our findings. In the same study, the authors also showed that a GPI-anchored TEM8 ectodomain is sufficient to mediate toxin entry, emphasizing that these experiments define minimal requirements for intoxication, rather than the regulation of receptor behavior.
Since those initial studies, including the follow-up work on CMG2 (Cellular Microbiology, 2007), multiple disease-associated missense mutations mapping to the cytosolic domain of CMG2 have been identified. We and others have shown that this region controls interactions with the actin cytoskeleton and is required for regulated endocytosis and collagen VI degradation (Bürgi et al., Dev Cell, 2021). In the present

work, we further demonstrate that S-acylation of the cytosolic tail governs these interactions and their ligand-dependent release.
Taken together, the earlier truncation studies and our results support a model in which cytosolic tail-less CMG2/TEM8 variants bypass regulatory constraints that normally control the behavior of the full-length receptor. This is now mentionned in the discussion.

2. The study relies heavily on siRNA knockdown of ZDHHC3 and ZDHHC7 to assess their roles in CMG2 palmitoylation, yet no data are provided to demonstrate the extent of knockdown for these enzymes.
We agree that this information is important. These control experiments had been performed but were not included in the original submission. The extent of knockdown for ZDHHC3 and ZDHHC7 is now shown in Extended Data Fig. 2c.

3. The authors used the pan thioesterase inhibitor Palmostatin B (more selective for APT1) in Fig. 3J and ED Fig. 2d. Why was the more APT2 specific inhibitor ML349 not used in these experiments?
The rationale for using Palmostatin B in these experiments is primarily technical, as now mentionned in the text. At the time these pulse-chase experiments were initiated, Palmostatin B was the most robustly validated thioesterase inhibitor for stabilizing S-acylated proteins over extended chase periods, and it consistently produced strong and reproducible effects in metabolic labeling assays. ML349, while more selective for APT2, has a shorter effective window in these long pulse-chase conditions and we thought that it was therefore less suited for these particular experiments.
Importantly, the conclusions drawn from the Palmostatin B experiments are fully supported by multiple independent lines of evidence throughout the manuscript demonstrating a specific role for APT2 in CMG2 deacylation, including APT2 knockdown, knockout, rescue experiments, and acute inhibition with ML349 in signaling and functional assays. Thus, the use of Palmostatin B in Fig. 3j and Extended Data Fig. 3e does not alter the interpretation that APT2 is the relevant thioesterase acting on CMG2.

4. Fig. 7c: The LF mediated cleavage of MEK2 is difficult to evaluate without information on the specificity of the MEK2 N antibody. MEK3 cleavage is typically easier to detect and could strengthen the analysis. Although the cell line used may not be highly sensitive to LF, the authors could test LF DT or LF ETA fusion to determine whether the APT2 inhibitor ML349 protects cells from toxin induced effects. The APT2 KO cells shown in Fig. 7a could also be assessed for toxin susceptibility.
We appreciate this thoughtful comment.
The initial submission of the manuscript indeed did not contain a precise description of the antibodies used.We now clearly describe in the figure legends and Methods that the antibody used was generated against the N-terminus of MEK2 and does not recognize the LF-processed form, which is rapidly degraded in any case.
Importantly, we have substantial prior experience with this antibody, including its use in a previously published functional screen of anthrax toxin entry and activity, where it provided a robust and quantitative readout of LF activity (Anwar et al., Dev. Cell 2022).

We have however followed the reviewers advice of using anti-MEK3 staining in Fig. 7ab, which indeed was robust.
Susceptibility of APT2 KO cells was shown in the corresponding Extended Figure, we have now emphasize this in the text.

Finally, with respect to the use of additional readouts, we have now added a dedicated figure (Fig. 8), in which we tested PalmB and ML349 in an established zebrafish model of anthrax lethal toxin intoxication that reports vascular dysfunction (Bolcome et al., doi.org/10.1073/pnas.0712195105). We reproduced the published phenotype and show that ML349 robustly and dose-dependently attenuates toxin-induced vascular defects, resulting in a clear functional rescue in vivo. These findings substantially strengthen and extend our conclusions and highlight the therapeutic potential of targeting deacylation in the context of anthrax intoxication.

5. ED Fig. 6b–d: LF mediated cleavage of MEK1 is difficult to discern. In ED Fig. 6d, the cleavage pattern appears similar between APT2 KO and APT2 KO + APT2 rescue conditions.
We thank the reviewer for drawing attention to this point. Upon re-examination, we realized that the blot in Extended Data Fig. 6d was mislabeled, Ctrl RPE1 cells are shown next to APT2 KO +APT2. The figure has now been corrected.

6. Because APT2 appears to have opposing roles, negative effect on early CMG2 biogenesis but a positive role in ligand induced internalization, it is unclear how APT2 could be considered a viable therapeutic target for anthrax, as suggested by the authors.
We agree with the reviewer that APT2 plays temporally distinct roles in CMG2 biology, with opposing consequences depending on the cellular stage. In the early secretory pathway, APT2 activity counteracts CMG2 S-acylation and promotes clearance through ERAD, whereas at the plasma membrane APT2 activity is required for ligand-induced deacylation, cytoskeletal disengagement, and internalization.
Importantly, these opposing roles occur at different stages of the CMG2 life cycle and are therefore not simultaneously engaged, for a given molecule. In the context of anthrax intoxication, APT2 inhibition would be acute and short-term, aimed at blocking toxin-induced internalization at the plasma membrane. Such transient inhibition is unlikely to cause major side effects associated with altered CMG2 biogenesis, while effectively preventing toxin entry during infection.
In contrast, for Hyaline Fibromatosis Syndrome, we agree that sustained and complete APT2 inhibition would not be desirable, as it would increase CMG2 abundance while impairing its signaling function. However, pharmacological inhibition is inherently dynamic, and fluctuating drug levels would be expected to create alternating phases in which CMG2 folding is promoted (during higher inhibition) and receptor function is preserved (as inhibition wanes). This suggests that dosing and timing could be leveraged to bias the system toward improved CMG2 expression without chronically suppressing its activity.
Thus, while APT2 inhibition might not be a universally beneficial intervention, our data support the idea that it represents a viable and context-dependent therapeutic target, particularly for acute blockade of anthrax toxin entry.
We have modified the discussion along these lines.

Minor comments:
1. Words appear to be missing at the end of line 173.
The end of this paragraph was inadvertently deleted during editing and has now been fully restored.

2. The titles in Fig. 4c (“Ctrl,” “siRNA CHC”) are confusing and should be clarified.
This information was indeed missing from the legend, we thank the reviewer for picking this up. The legend has now been completed.

3. Fig. 2f and Fig. 7g are low in quality and difficult to read.
The quality and size of the images has been improved.

Point-by-Point Reply

Reviewer 4:

1. The legends for Fig. 1f appear to be missing.
We ave now added the legend for Fig. 1f.

2. In Fig. 2, cycloheximide treatment does not seem to affect total CMG2 protein levels. Please provide an explanation for this observation.
The cycloheximide treatment was only 1 hr and thus is not expected to change the amount of CMG2 detected by WB which monitores the total amount. To avoid confusion, we have chosen not to comment on this point in the manuscript.

3. CMG2 ligand binding triggers TVA release as well as the recruitment of RhoA, β arrestin, and mDia. While TVA release is clearly critical for CMG2 internalization, please comment on whether the recruitment of RhoA, β arrestin, and mDia is functionally important for CMG2 receptor activity.
In addition to the previous mention of the importance of ß-arrestin and RhoA for ligand-induced endocytosis in the introduction, we now also mention this point in the result section and the discussion.
