# Supplementary material for: Dynamic S-acylation controls CMG2 maturation extracellular matrix regulation and anthrax toxin susceptibility in vivo
Source: Nat Commun. 2026 Jul 6;17:8364. doi: 10.1038/s41467-026-75028-2 (PMC13473502; doi:10.1038/s41467-026-75028-2)

Supplementary Information

Supplementary Figure 1: S-acylation of CMG2 cysteine mutants.

HeLa cells were transfected for 24 h with WT and mutants CMG2-V5. Cells were labeled for 2 h with [^{3}H]-palmitate at 37 °C. **a**. Total Cell extracts (TCEs) were analyzed by Western blot against CMG2. The signal was quantified over multiple experiments (n=6). ****p<0.0001 **b.** CMG2-V5 was immunoprecipitated. Autoradiography and anti-V5 immunoblotting were used to detect S-acylated and CMG2, respectively. The [^{3}H]-palmitate signal was quantified over multiple experiments (n=6).

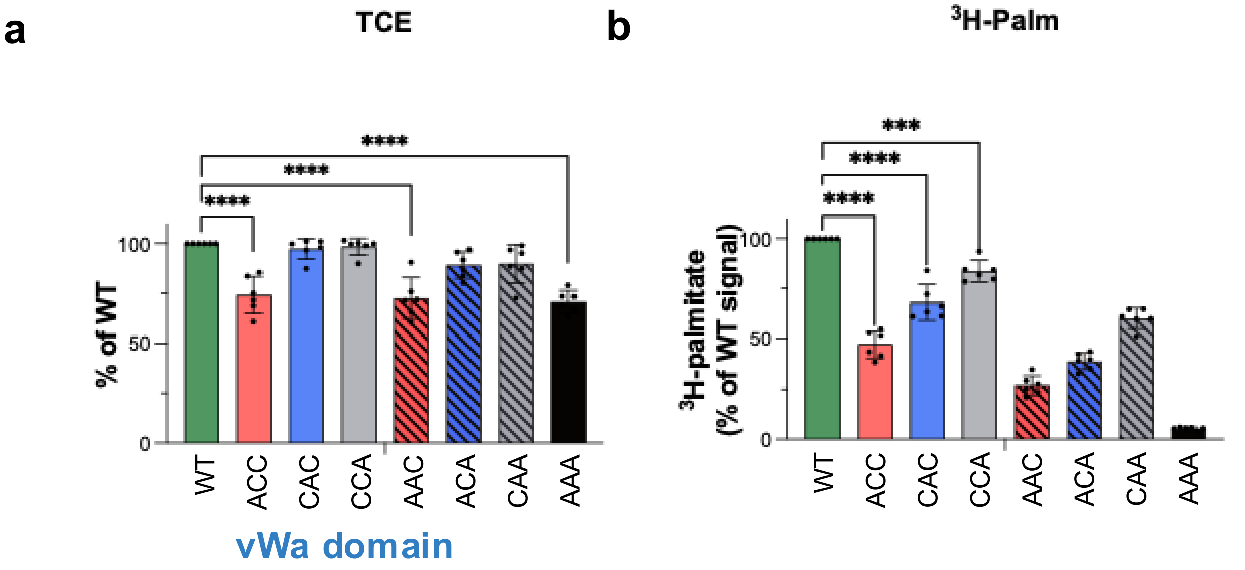
a
TCE
****
****
****
100
50
0
% of WT
WT
ACC
CAC
CCA
AAC
ACA
CAA
AAA
vWa domain
b
3H-Palm
***
****
****
100
50
0
3H-palmitate
(% of WT signal)
WT
ACC
CAC
CCA
AAC
ACA
CAA
AAA

Supplementary Figure 2: ZDHHC3/7 are the primary acyltransferases for CMG2 and TEM8.

a,b. RPE1 cells were silenced for 3 days with pooled (a) or individual (b) siRNAs targeting human ZDHHC enzymes, or control siRNA. Cells were metabolically labeled for 2 h at 37 °C with [^{3}H]-palmitic acid, CMG2 was immunoprecipitated with goat anti-human CMG2, and samples were analyzed by SDS–PAGE followed by autoradiography (^{3}H-palm) and anti-CMG2 immunoblotting. [^{3}H]-palmitate incorporation was quantified with a Typhoon scanner and normalized to control = 100% (n=3, mean ± SD). **c.** RPE1 cells were silenced for 3 days with depicted siRNAs. mRNA was extracted and quantitative RT-PCR were perfomed to measure mRNA transcript levels (mean ± SD). **d**. RPE1 cells were treated with siRNAs against ZDHHC3 or ZDHHC7 expression, or control siRNA. Last 24 hours, ZDHHC3 or ZDHHC7 were overexpressed. Cells were metabolically labelled for 2 h at 37 °C with [^{3}H]-palmitic acid, CMG2 was immunoprecipitated with goat anti-human CMG2, and samples were analysed by SDS–PAGE followed by autoradiography (^{3}H-palm) and anti-CMG2 immunoblotting. [^{3}H]-palmitate incorporation was quantified with a Typhoon scanner and normalized to control = 100% (n=3, mean ± SD). **e**. HeLa cells were silenced for 3 days with pooled or individual siRNAs against human ZDHHC enzymes, metabolically labeled with [^{3}H]-palmitate for 2 h at 37 °C, and TEM8 was immunoprecipitated with goat anti-TEM8. Samples were analyzed by SDS–PAGE, autoradiography, and anti-TEM8 immunoblotting. Radiolabel incorporation was quantified by Typhoon imaging and normalized to control = 100% (n=3, mean ± SD). **f.** Silencing of ZDHHC3 or ZDHHC7 markedly reduced [^{3}H]-palmitate incorporation into TEM8, confirming that these two acyltransferases are the principal determinants of receptor palmitoylation. Quantification was performed as in a.

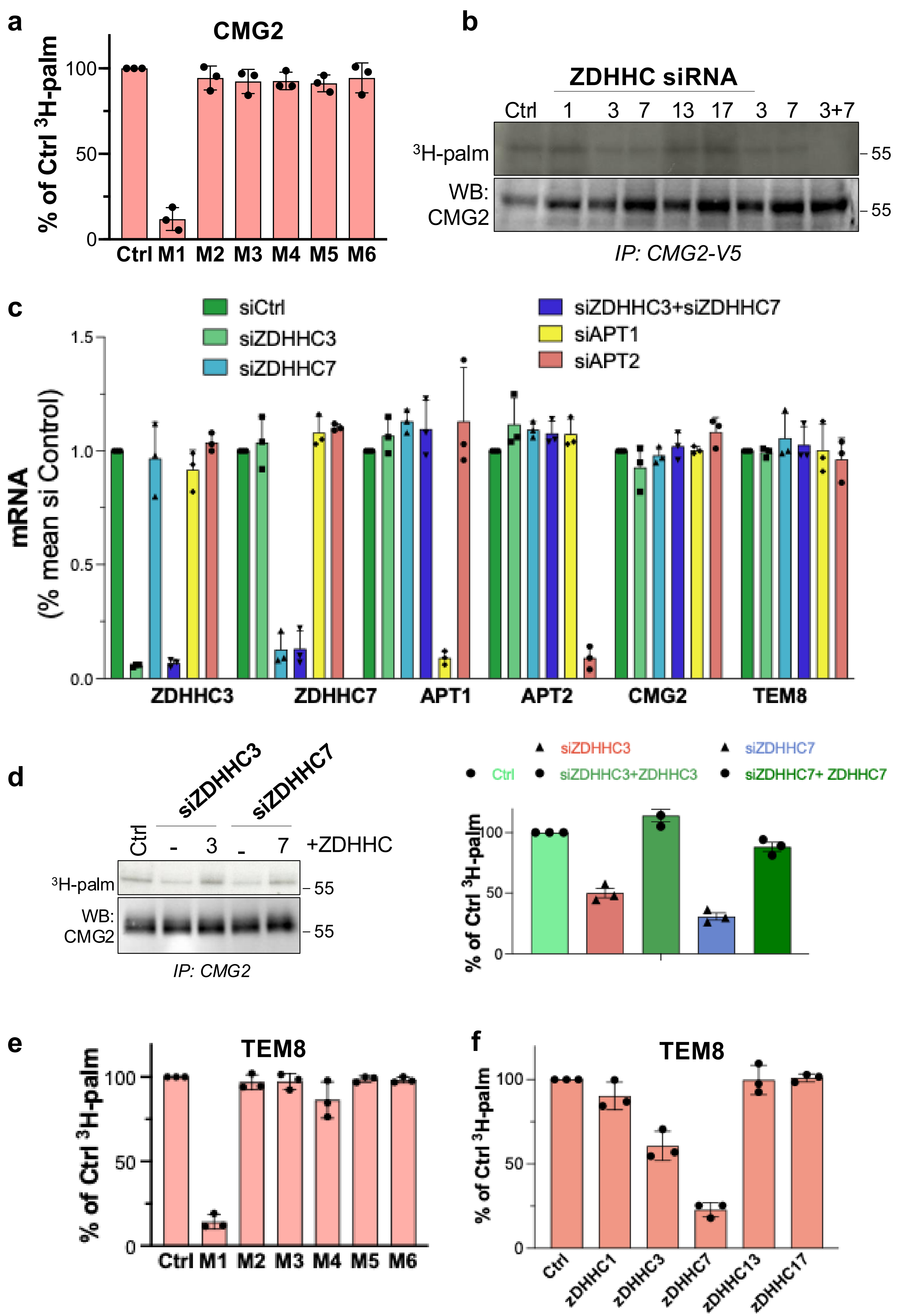
a
CMG2
% of Ctrl ³H-palm
100
50
0
Ctrl M1 M2 M3 M4 M5 M6
b
ZDHHC siRNA
Ctrl 1 3 7 13 17 3 7 3+7
³H-palm
WB: CMG2
55
55
IP: CMG2-V5
c
siCtrl
siZDHHC3
siZDHHC7
siZDHHC3+siZDHHC7
siAPT1
siAPT2
mRNA (% mean si Control)
1.5
1.0
0.5
0.0
ZDHHC3 ZDHHC7 APT1 APT2 CMG2 TEM8
d
Ctrl
siZDHHC3
siZDHHC7
- 3 - 7 +ZDHHC
³H-palm
WB: CMG2
55
55
IP: CMG2
Ctrl
siZDHHC3
siZDHHC3+ZDHHC3
siZDHHC7
siZDHHC7+ ZDHHC7
% of Ctrl ³H-palm
100
50
0
e
TEM8
% of Ctrl ³H-palm
100
50
0
Ctrl M1 M2 M3 M4 M5 M6
f
TEM8
% of Ctrl ³H-palm
100
50
0
Ctrl
zDHHC1
zDHHC3
zDHHC7
zDHHC13
zDHHC17

Supplementary Figure 3: S-Acylation–deacylation of CMG2.

a. HeLa cells expressing CMG2-V5 WT or AAA were silenced for 72 h with siZDHHC3+siZDHHC7 or control RNAi, pulse-labeled 20 min with [^{35}S]-Met and chased as indicated; CMG2-V5 was immunoprecipitated and analyzed by SDS–PAGE, autoradiography and immunoblotting, and radiolabel incorporation quantified (Typhoon) and normalized to WT/no RNAi = 100% (n=3, mean ± SD). **b.** Table with the apparent half-lifes of CMG2 variants and how these are affected by ZDHHC3 and 7 silencing or by inhibition of deacylation**.** RPE1 cells were treated 4 h before and during labeling with Palmostatin B (50 µM), ML348 (10 µM) or ML349 (10 µM), labeled 3 h with [^{3}H]-palmitate and chased 5 h; CMG2 immunoprecipitates were analyzed by SDS–PAGE and autoradiography. **d**. RPE1 cells were transfected with siAPT2 for 72 h, labeled 2 h with [^{3}H]-palmitate and chased; CMG2 was immunoprecipitated and analyzed by SDS–PAGE, autoradiography and immunoblotting. **e.** HeLa cells expressing CMG2-V5 WT or AAA were treated ± Palmostatin B (50 µM; 4 h before and during labeling), pulse-labeled 20 min with [^{35}S]-Met and chased; CMG2-V5 decay was quantified as in a. and normalized to WT/no drug = 100% (n=3, mean ± SD). **f.** ^{35}S-Cys/Met values at t=5hrs from Figs. 3c,h,I were plotted to illustrate the impact of S-acylation of CMG2 abundance after a 5hrs chase. **g.** Hela cells expressing CMG2-V5 WT and different point mutations were co-transfected with APT2 WT for 24h, immunoprecipitated with anti-V5 CMG2 and analyzed by SDS-PAGE and immunoblotting using anti-FLAG APT2 or anti-V5 CMG2. **h.** Mice received ML349 intraperitoneally (40 mg/kg on days 0, 2, 4, 6, 8); colon was collected at indicated times and CMG2 abundance assessed by immunoblotting of total lysates (40 µg) with anti-CMG2 and anti-GAPDH. **i.** Different tissues were collected from mice described in **h**. mRNA were extracted and quantitative RT-PCR were performed to measure mRNA transcript levels of mouse CMG2, TEM8 or LRP6 (mean ± SD).

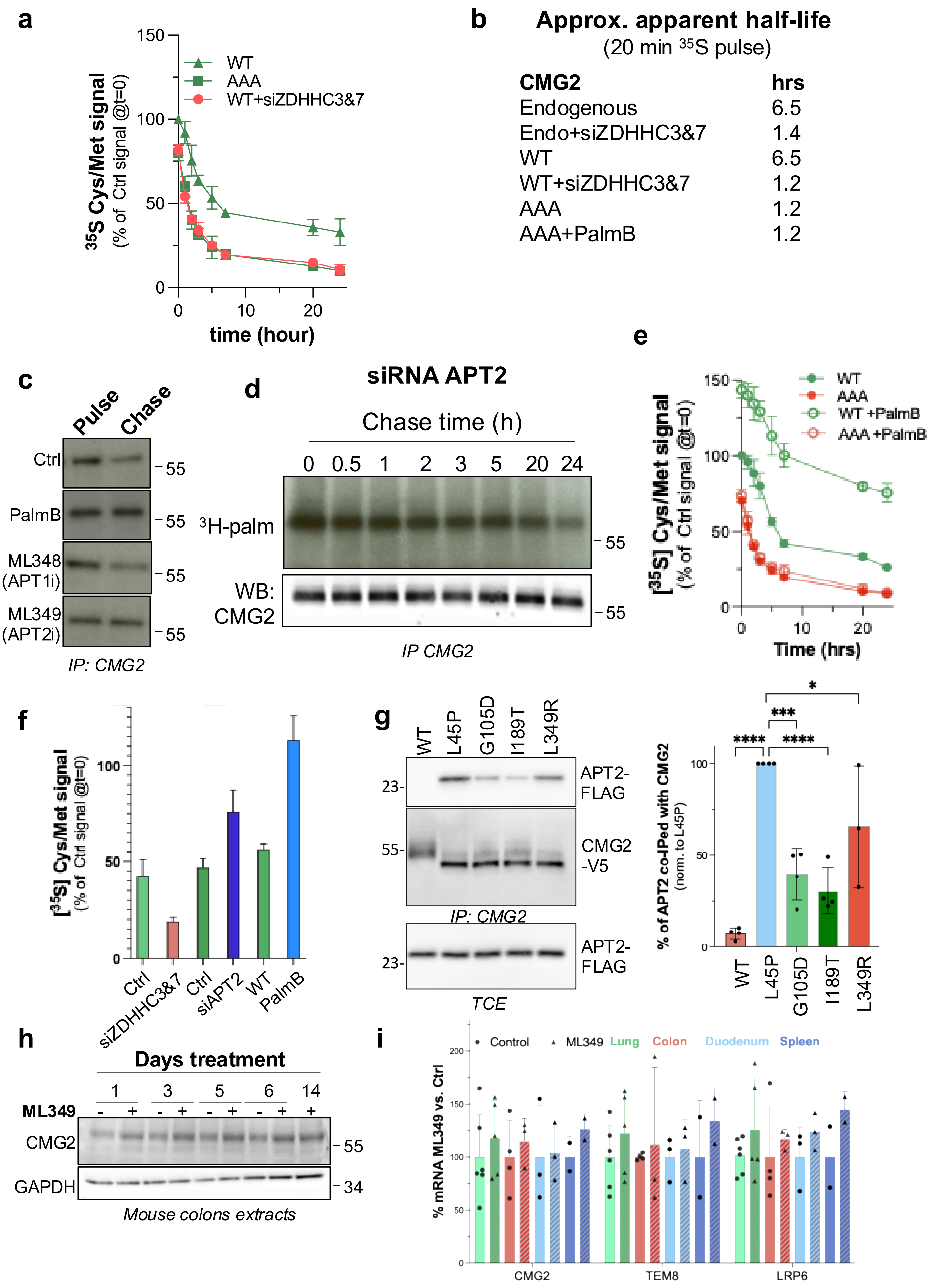
a
^{35}S Cys/Met signal
(% of Ctrl signal @t=0)
time (hour)
WT
AAA
WT+siZDHHC3&7
b
Approx. apparent half-life
(20 min ^{35}S pulse)
CMG2 hrs
Endogenous 6.5
Endo+siZDHHC3&7 1.4
WT 6.5
WT+siZDHHC3&7 1.2
AAA 1.2
AAA+PalmB 1.2
c
Pulse
Chase
Ctrl
PalmB
ML348 (APT1i)
ML349 (APT2i)
55
IP: CMG2
d
siRNA APT2
Chase time (h)
0 0.5 1 2 3 5 20 24
^{3}H-palm
WB: CMG2
IP CMG2
e
[^{35}S] Cys/Met signal
(% of Ctrl signal @t=0)
Time (hrs)
WT
AAA
WT +PalmB
AAA +PalmB
f
[^{35}S] Cys/Met signal
(% of Ctrl signal @t=0)
Ctrl
siZDHHC3&7
Ctrl
siAPT2
WT
PalmB
g
WT
L45P
G105D
I189T
L349R
APT2-FLAG
CMG2 -V5
IP: CMG2
APT2-FLAG
TCE
% of APT2 co-IPed with CMG2
(norm. to L45P)
h
Days treatment
1 3 5 6 14
ML349
CMG2
GAPDH
Mouse colons extracts
i
Control
ML349
Lung
Colon
Duodenum
Spleen
% mRNA ML349 vs. Ctrl
CMG2
TEM8
LRP6

Supplementary Figure 4: CMG2 ligands trigger the TVA to RhoA signalling switch.

a. HeLa cells were transfected for 48 h with CMG2-V5 WT or cysteine mutants, switched to serum-free medium 24 h prior, and then incubated ± PA (500 µg/ml; 1 h at 4 °C followed by 30 min at 37 °C) or with 10% serum. CMG2-V5 was immunoprecipitated in PBS/1% Triton X-100 using anti-V5 and protein G for 4 h at 4 °C, resolved by SDS-PAGE, and blotted with anti-PA, anti-Vinculin, anti-Actin, anti-RhoA, anti-mDia and anti-V5. **b.** RPE1 cells were silenced for 72 h with siZDHHC3, siZDHHC7 or control RNAi, switched to serum-free medium 24 h prior, and stimulated ± PA (500 µg/ml; 1 h at 4 °C followed by 30 min at 37 °C) or with collagen IV or VI (1 µg/ml; 3 h at 37 °C). CMG2 was immunoprecipitated in PBS/1% Triton X-100 using anti-CMG2 and protein G for 4 h at 4 °C, resolved by SDS–PAGE, and analyzed by immunoblotting with anti-Talin, anti-Vinculin, anti-Actin, anti-RHOA and anti-CMG2-V5. **c**. RPE1 cells were labelled 2 h with [^{3}H]-palmitate at 37 °C, washed into chase medium for 2.5 h, then treated ± PA (500 µg/ml) ± MG132 (10 µM). CMG2 was immunoprecipitated with anti-CMG2, analysed by SDS-PAGE and autoradiography (^{3}H-palm) and immunoblotting with anti-CMG2 and anti-PA.

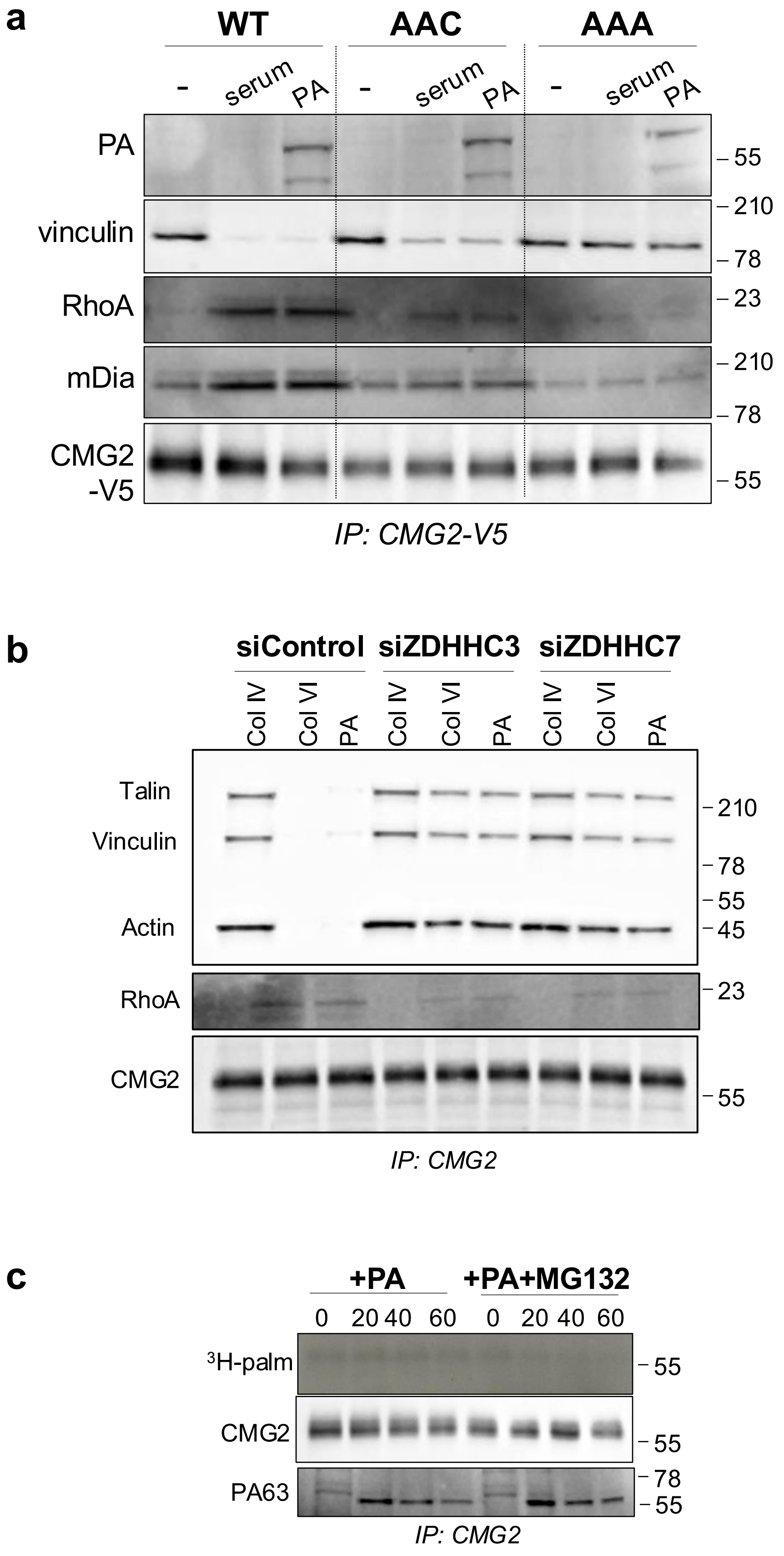
a
WT
AAC
AAA
-
serum
PA
PA
vinculin
RhoA
mDia
CMG2
-V5
55
210
78
23
210
78
55
IP: CMG2-V5
b
siControl
siZDHHC3
siZDHHC7
Col IV
Col VI
PA
Talin
Vinculin
Actin
RhoA
CMG2
210
78
55
45
23
55
IP: CMG2
c
+PA
+PA+MG132
0
20
40
60
³H-palm
CMG2
PA63
55
55
78
55
IP: CMG2

Supplementary Figure 5: Ligand binding triggers recruitment of membrane-anchored APT2 to CMG2.

RPE1 (**a,b,d,e**) or Hela (**c**) cells were serum-starved the day before, incubated or not for 1 h at 4 °C with PA (500 µg/ml), immunoprecipitated in PBS–1% TX-100 with anti-CMG2 (**a,b,d,e**) or anti-V5 (**c**) plus protein G for 4 h at 4 °C, and analyzed by SDS–PAGE and immunoblotting using anti-PA, anti-APT2-FLAG, anti-Talin, anti-Vinculin, anti-Actin and anti-CMG2 or anti-V5 antibodies. **a**. RPE1 cells were silenced for 3 days with APT2 RNAi and transfected during the last 24 h with APT2-FLAG WT, C2S or S121A. **b.** Control (Ctrl) or APT2-KO RPE1 cells were transfected for 24 h with APT2-FLAG WT. **c.** HeLa cells were transfected for 48 h with WT or W396L CMG2-V5 together with APT2-FLAG WT. **d.** APT2-KO RPE1 cells were transfected for 48 h with APT2-FLAG WT, PosPatch or M68E, **e**. APT2-KO RPE1 cells were transfected for 24 h with APT2-FLAG WT, and treated or not with ML349, before and during subsequent PA treatment. **f**. Quantification of **e**, n=4, mean ± SD. **g**. Hela cells were transfected with plasmids encoding WT APT2-FLAG for 24h. PNS were prepared and ultra-centrifuged to separate cytosolic fraction (sup.) from membrane fraction (Pellet). Equal volumes were loaded for SDS-PAGE and immunoblotting using anti-APT2-FLAG and anti-Calnexin antibodies. **h.** Purified APT2 proteins were incubated with liposomes and loaded on the bottom of a step sucrose gradient. The interfaces were collected from top (interf.1: between 10% and 30% sucrose; interf.2: between 30% and 40% sucrose) to bottom and analyzed by SDS-PAGE and Coomassie blue.

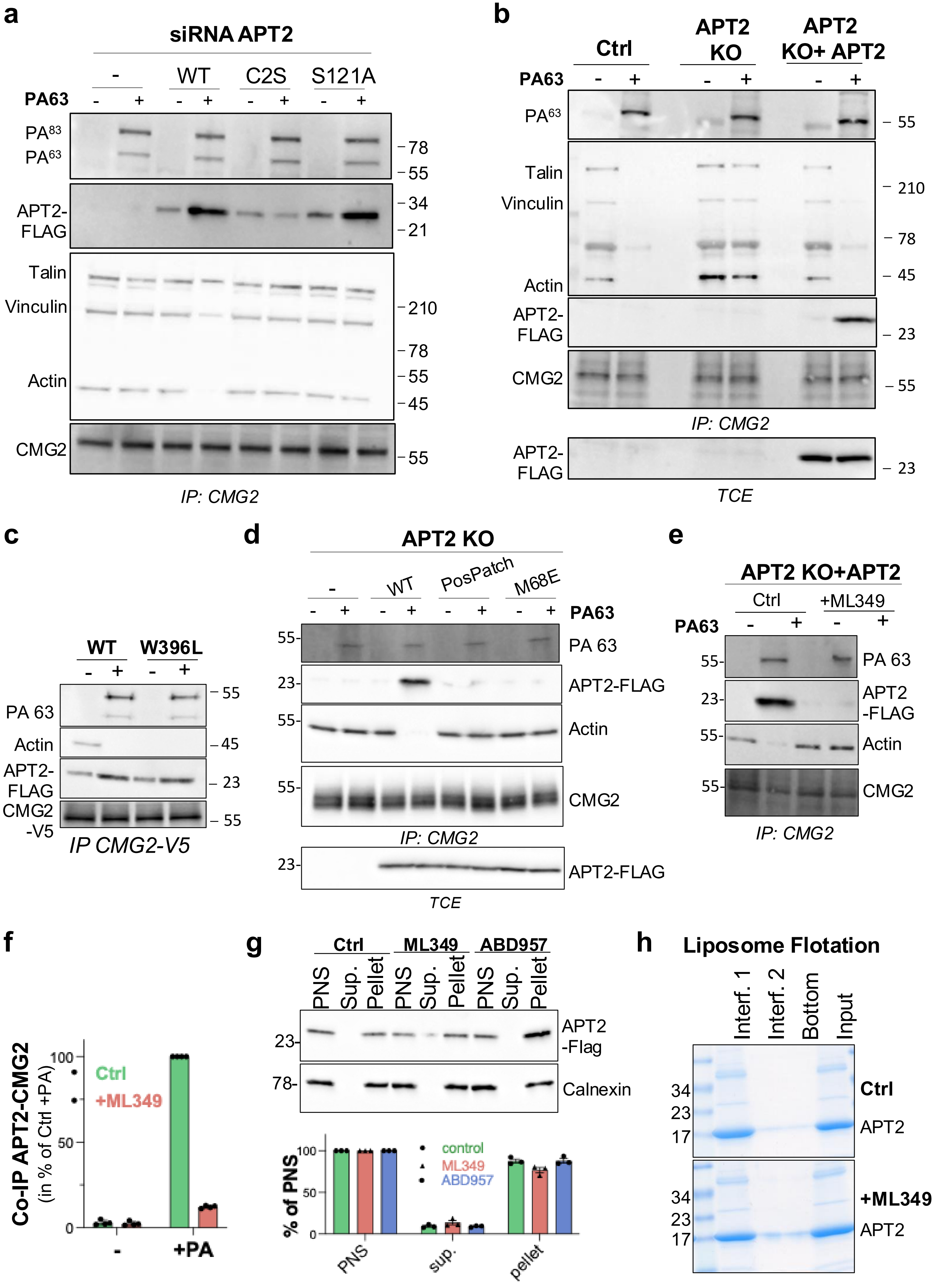
a
siRNA APT2
-
WT
C2S
S121A
PA63
PA83
PA63
APT2-FLAG
Talin
Vinculin
Actin
CMG2
78
55
34
21
210
78
55
45
55
IP: CMG2
b
Ctrl
APT2 KO
APT2 KO+ APT2
PA63
PA63
Talin
Vinculin
Actin
APT2-FLAG
CMG2
IP: CMG2
APT2-FLAG
TCE
55
210
78
45
23
55
23
c
WT
W396L
PA 63
Actin
APT2-FLAG
CMG2-V5
IP CMG2-V5
55
45
23
55
d
APT2 KO
-
WT
PosPatch
M68E
PA63
PA 63
APT2-FLAG
Actin
CMG2
IP: CMG2
APT2-FLAG
TCE
55
23
55
55
23
e
APT2 KO+APT2
Ctrl
+ML349
PA63
PA 63
APT2-FLAG
Actin
CMG2
IP: CMG2
55
23
55
55
f
Co-IP APT2-CMG2
(in % of Ctrl +PA)
Ctrl
+ML349
100
50
0
-
+PA
g
Ctrl
ML349
ABD957
PNS
Sup.
Pellet
APT2-Flag
Calnexin
23
78
% of PNS
control
ML349
ABD957
100
50
0
PNS
sup.
pellet
h
Liposome Flotation
Interf. 1
Interf. 2
Bottom
Input
34
23
17
Ctrl
APT2
+ML349
APT2

Supplementary Figure 6: RPE1 cells were pre-incubated 4 hours at 37°C with 10µg/ml ML348 or ML349 or were silence for APT2 expression during 72 hrs. Cells were incubated 1 hour at 4°C with 500µg/ml PA and 50ng/ml LF and chased different indicated times at 37°C. **a**. 40 µg of protein samples were acidified to convert all PA oligomers to the SDS resistant form, submitted to SDS-PAGE, and analyzed by immunoblotting with rabbit anti-PA or rabbit anti-MEK2 N-terminal. The amounts of PA oligomers were quantified, and plotted (n=4, mean ± SD). **b**. 40 µg of protein samples were submitted to SDS-PAGE, and analyzed by immunoblotting with rabbit anti-human CMG2, rabbit anti-PA or rabbit anti-MEK1 N-terminal. **c**, **d**. Control and APT2 KO RPE1 were treated and analyzed as in **ab**.

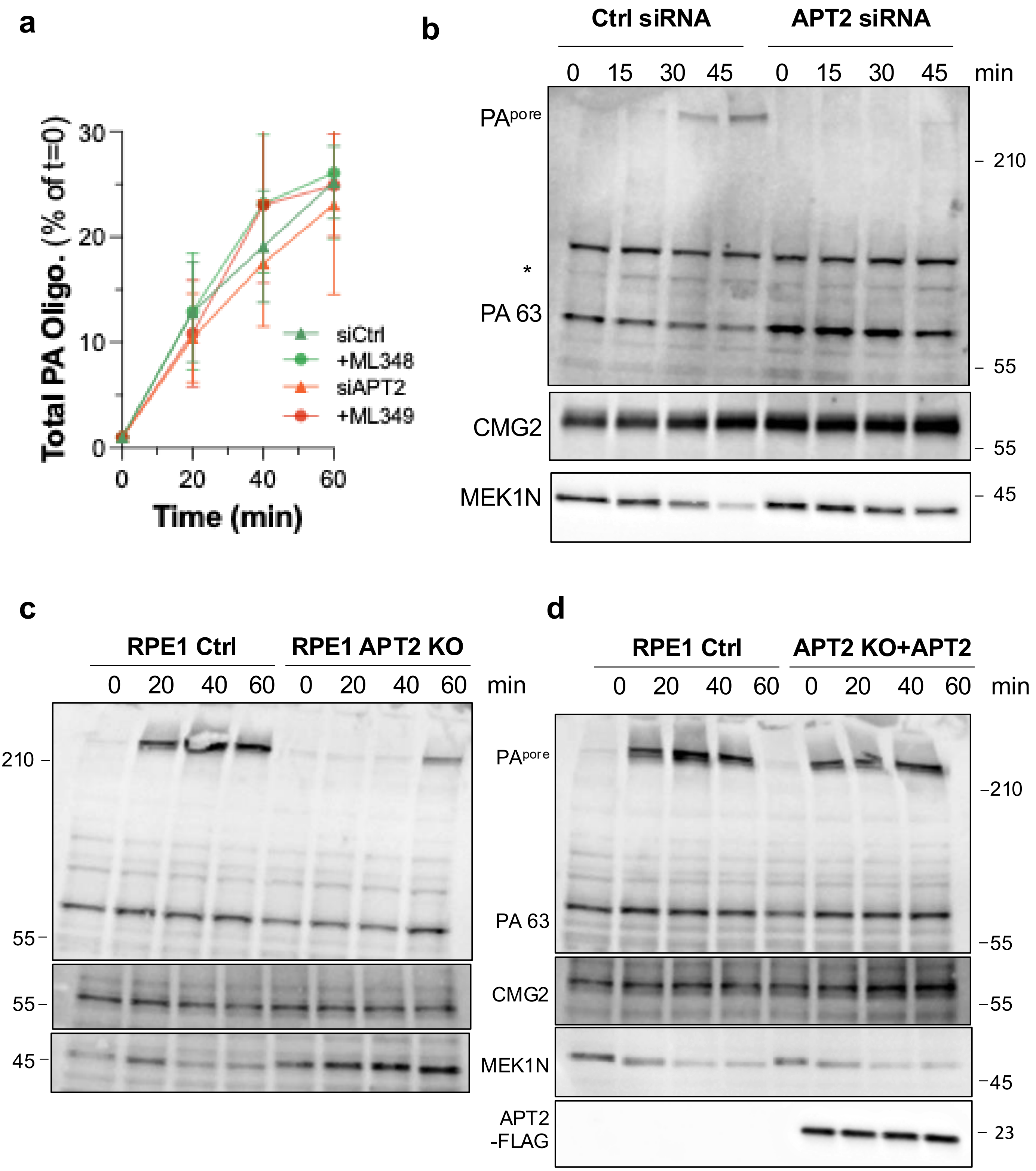
a
Total PA Oligo. (% of t=0)
30
20
10
0
0
20
40
60
Time (min)
siCtrl
+ML348
siAPT2
+ML349
b
Ctrl siRNA
APT2 siRNA
0
15
30
45
0
15
30
45
min
PApore
*
PA 63
210
55
CMG2
55
MEK1N
45
c
RPE1 Ctrl
RPE1 APT2 KO
0
20
40
60
0
20
40
60
min
210
55
55
45
d
RPE1 Ctrl
APT2 KO+APT2
0
20
40
60
0
20
40
60
min
PApore
210
PA 63
55
CMG2
55
MEK1N
45
APT2
-FLAG
23

Supplementary Figure 7: EGF uptake and endo-lysosomal trafficking are not impaired in APT2 KO cells. a. Spinning disk automated microscope confocal images showing RPE1 and RPE1 APT2 KO cells incubated during 15min at 37°C with 0.5 µg/ml A647-EGF and chased for the specified times. **b**. Graph showing the integrated intensity sum of EGF dots per cell after 15 min of A647-EGF uptake in RPE1 vs RPE1 APT2 KO transfected or not with APT2-citrin. **c**. Graph showing the normalized EGF dots integrated intensity sum per cell at the different timepoints of the pulse chase experiment in both Control and APT2 KO cells transfected. For each data point on the different graphs, a minimum of 1000 cells were quantified. Data represents the mean ±SD.

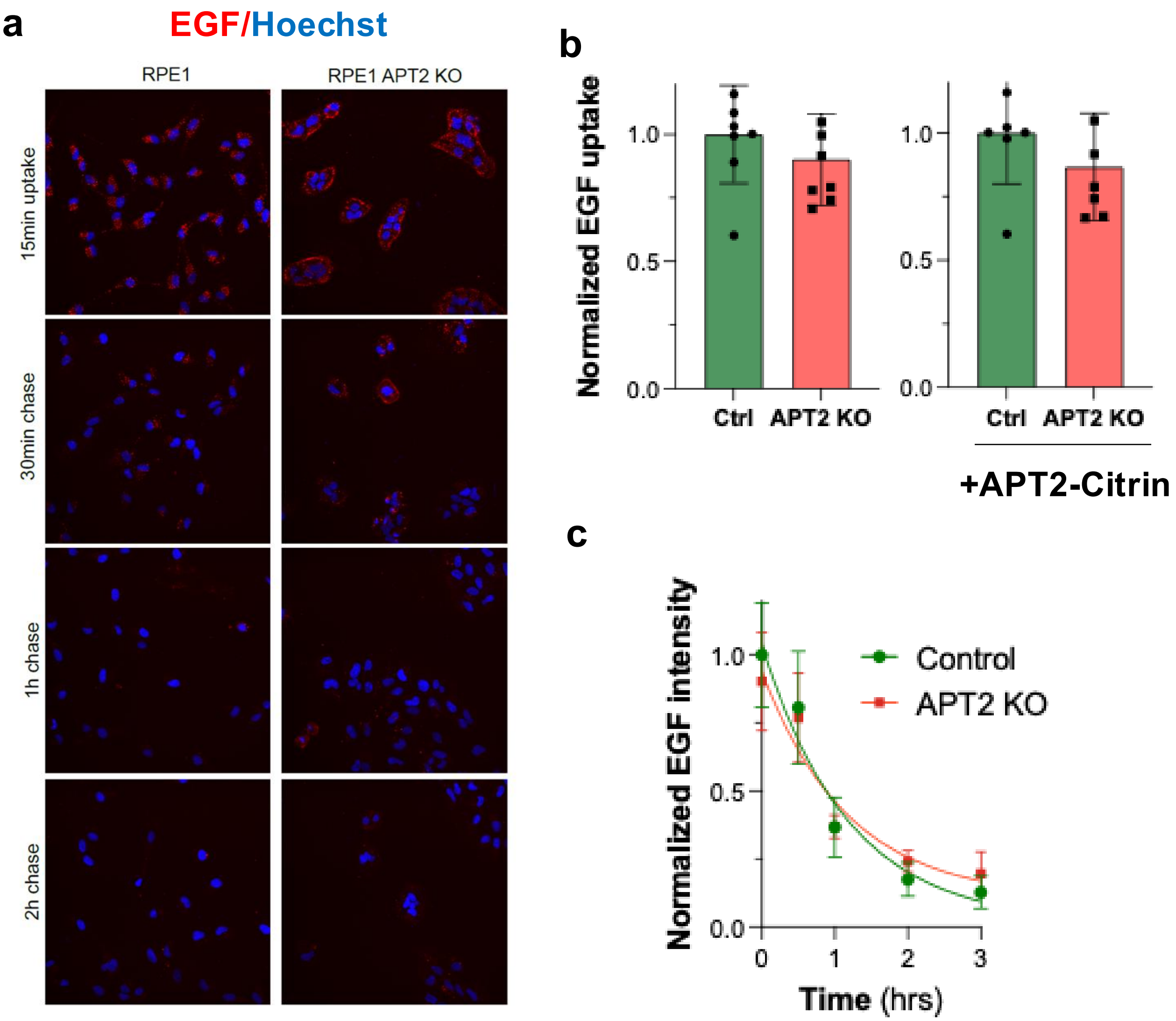
a
EGF/Hoechst
RPE1
RPE1 APT2 KO
15min uptake
30min chase
1h chase
2h chase
b
Normalized EGF uptake
1.0
0.5
0.0
Ctrl
APT2 KO
+APT2-Citrin
c
Normalized EGF intensity
Control
APT2 KO
Time (hrs)

Supplementary videos

Video recording of live Tg(*fli1*:eGFP; *gata1*:dsRed) embryos at 3dpf, under the different experimental conditions: control, Lethal Toxin (LeTx), and Lethal Toxin combined with either 100µM Palmostatin B (LeTx + PalmB), or 50µM ML349 (LeTx + ML349). gata1:DsRed expression is visualized using the fire or red lookup table in ImageJ. Note that PalmB and ML349 treatments restore red blood cell axial circulation and ISV perfusion. Scale bar represents 100µm.

Supplementary Movie 1: Live imaging of a Tg(fli1:eGFP; gata1:dsRed) zebrafish embryo at 3 dpf under control conditions.

Red blood cell circulation throughout the vasculature in a control embryo injected with PBS. Scale bar represents 100µm.

Supplementary Movie 2: Live imaging of a Tg(fli1:eGFP; gata1:dsRed) zebrafish embryo at 3 dpf injected with Lethal Toxin.

Red blood cell circulation is completely blocked throughout the embryonic vasculature upon Lethal toxin injection. Scale bar represents 100µm.

Supplementary Movie 3: Live imaging of a Tg(fli1:eGFP; gata1:dsRed) zebrafish embryo at 3 dpf co-injected with Lethal Toxin and 100µM Palmostatin B.

Co-injection of Palmostatin B partially restores the circulation defects in the ISVs, aorta and caudal vein caused by Lethal Toxin intoxication. Scale bar represents 100µm.

Supplementary Movie 4: Live imaging of a Tg(fli1:eGFP; gata1:dsRed) zebrafish embryo at 3 dpf co-injected with Lethal Toxin and 50µM ML349.

Co-injection of ML349 partially restores the circulation defects in the ISVs, aorta and caudal vein caused by Lethal Toxin intoxication. Scale bar represents 100µm.

Supplementary Movie 5: Live imaging of Tg(fli1:eGFP; gata1:dsRed) zebrafish embryos at 3 dpf under control conditions.

Red blood cell circulation throughout the vasculature in control embryos injected with PBS.

Supplementary Movie 6: Live imaging of Tg(fli1:eGFP; gata1:dsRed) zebrafish embryos at 3 dpf treated with Lethal Toxin.

Red blood cell circulation is dramatically impaired in the embryonic vasculature upon Lethal toxin injection.

Supplementary Movie 7: Live imaging of Tg(fli1:eGFP; gata1:dsRed) zebrafish embryos at 3 dpf treated with Lethal Toxin and 100µM Palmostatin B.

Co-injection of Palmostatin B significantly restores the circulation defects caused by Lethal Toxin in the embryonic vasculature.

Supplementary Movie 8: Live imaging of Tg(fli1:eGFP; gata1:dsRed) zebrafish embryos at 3 dpf

treated with Lethal Toxin and 50µM ML349.

Co-injection of ML349 significantly restores the circulation defects caused by Lethal Toxin in the embryonic vasculature.

Figure 1b

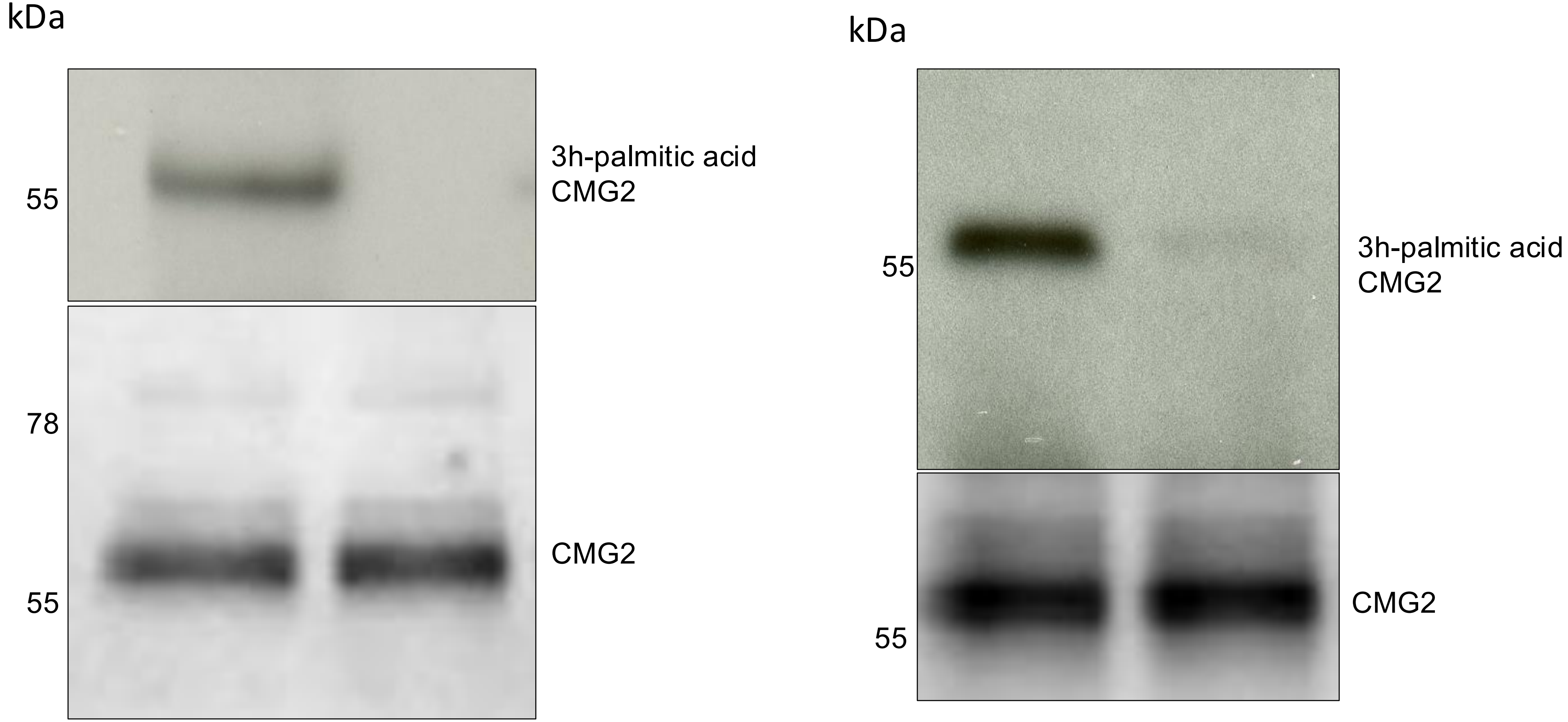
kDa
55
78
55
3h-palmitic acid
CMG2
CMG2
kDa
55
55
3h-palmitic acid
CMG2
CMG2

Figure 1c

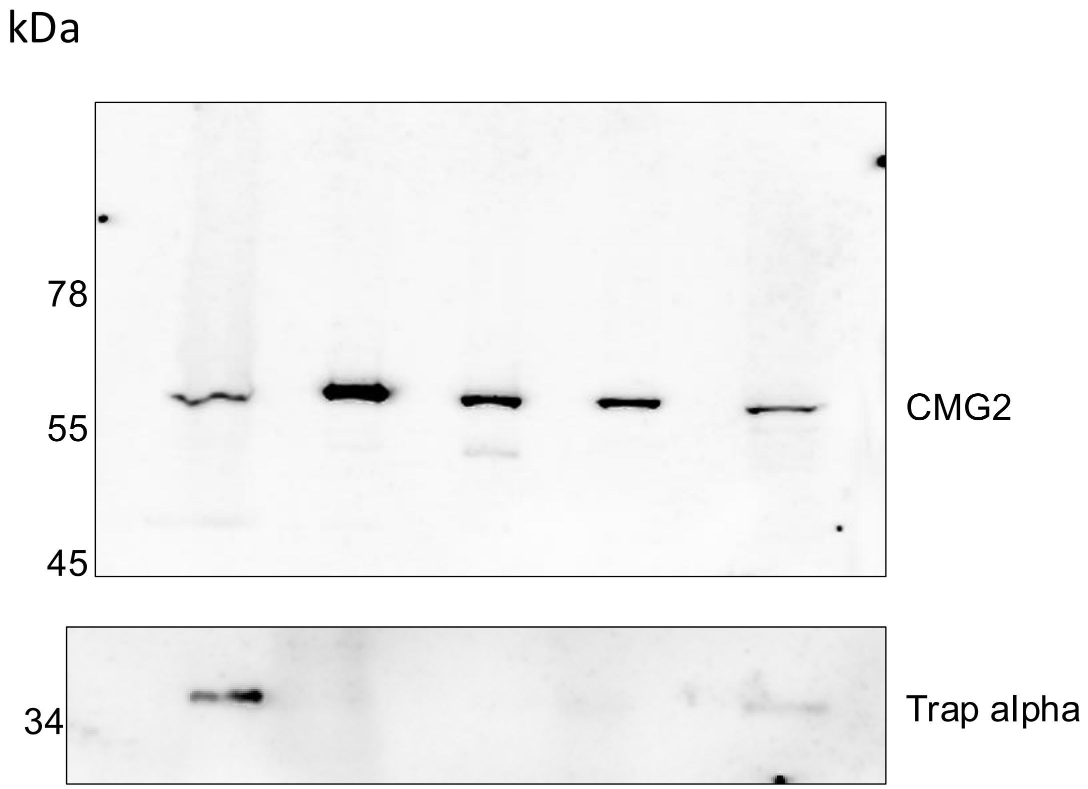

Figure 1d

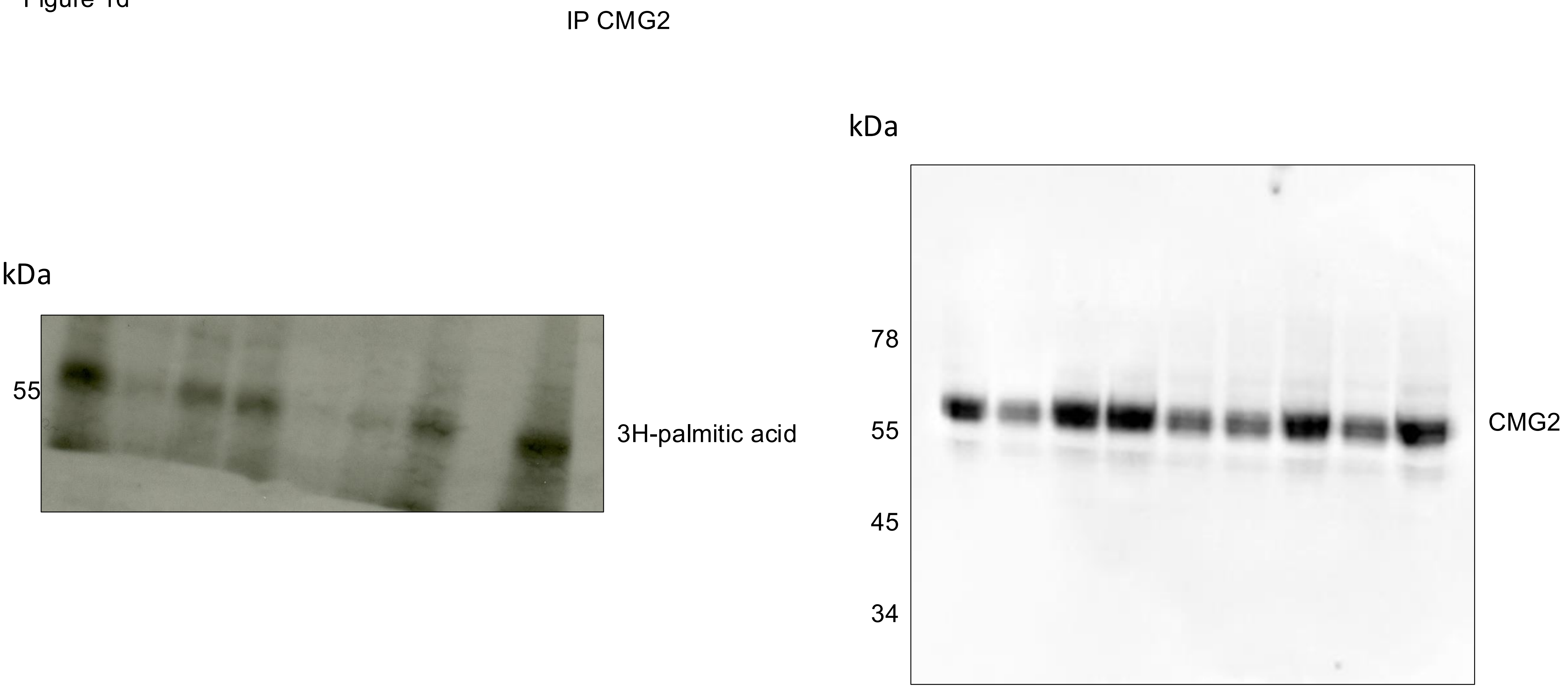

Figure 1f

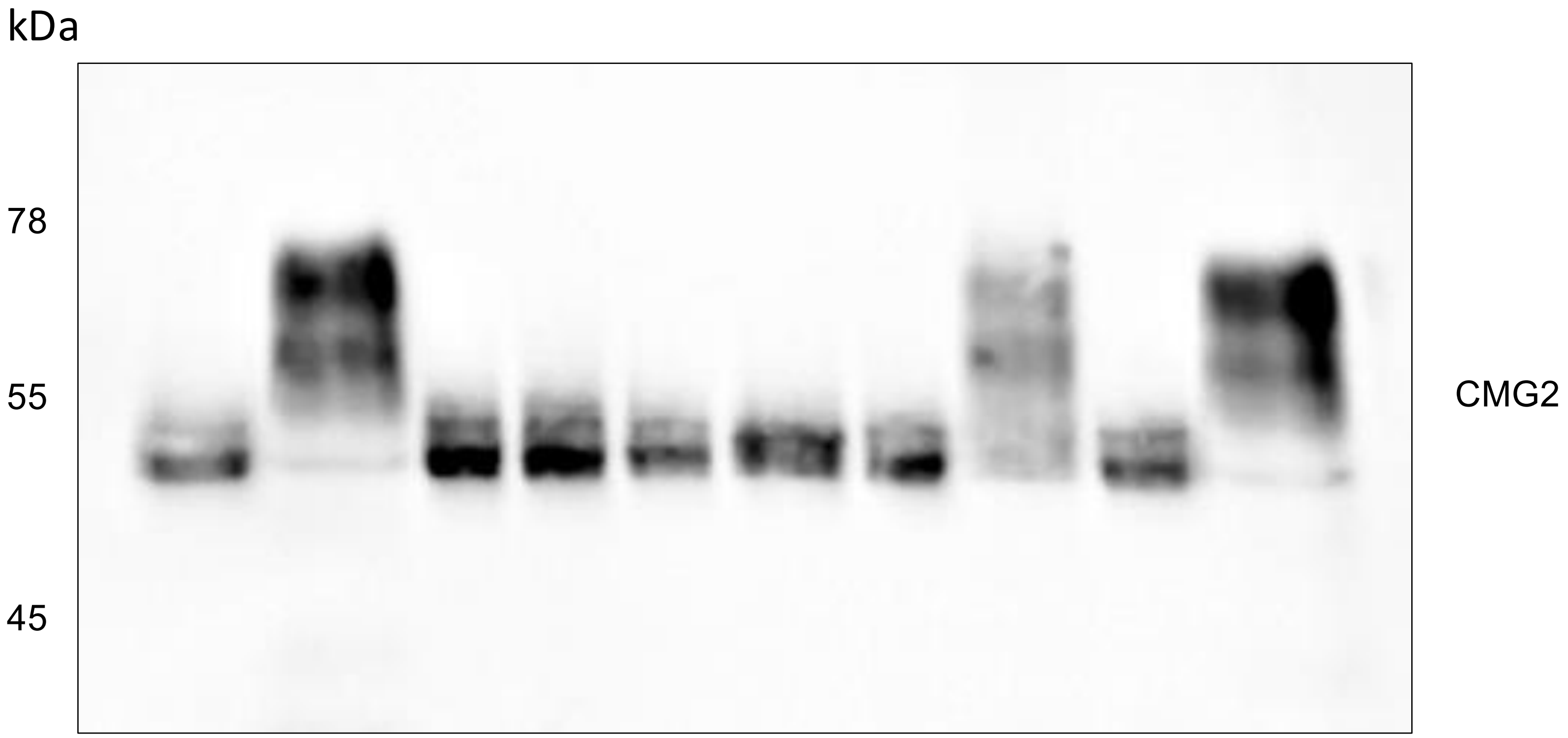

Figure 2a

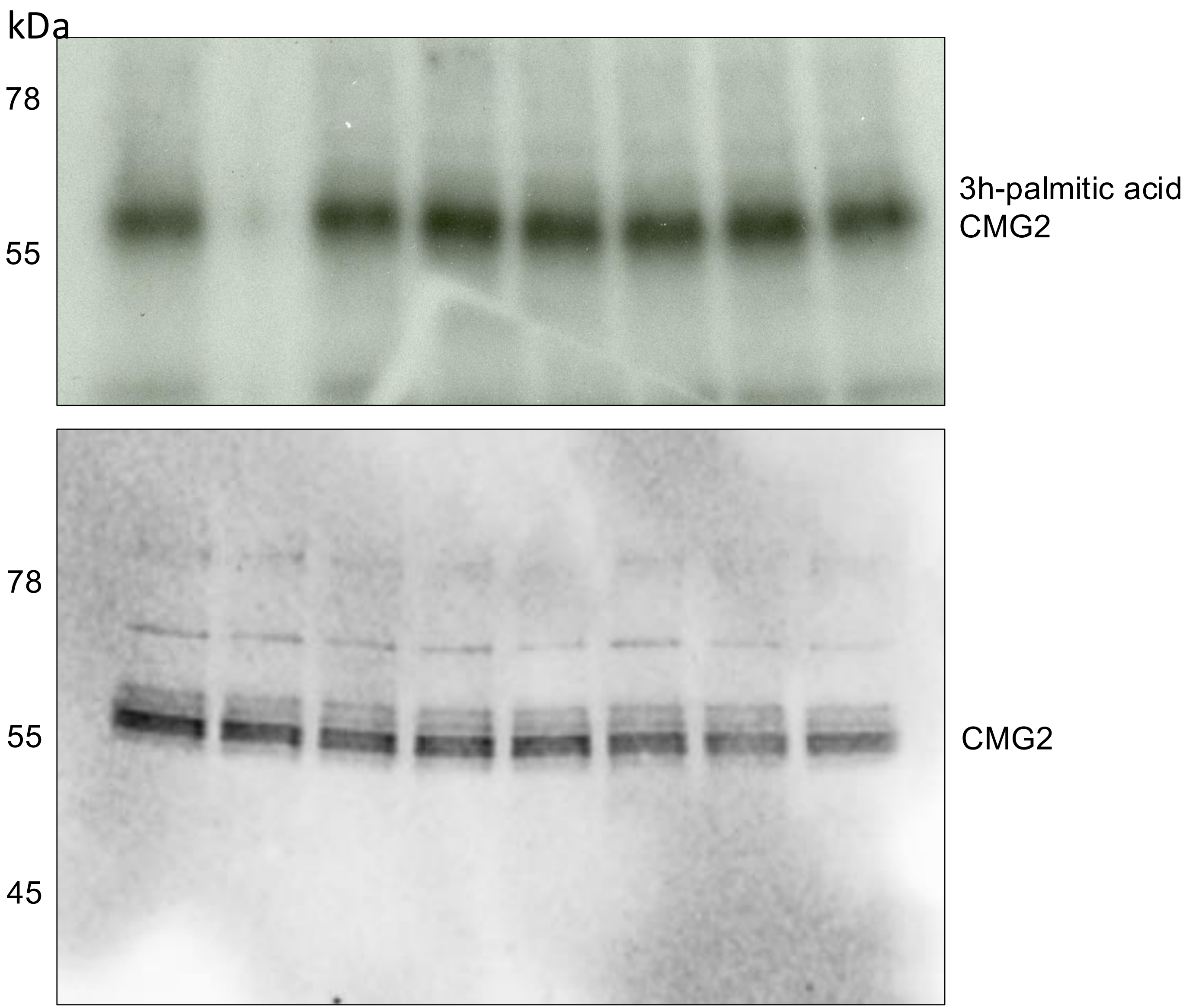

Figure 2c

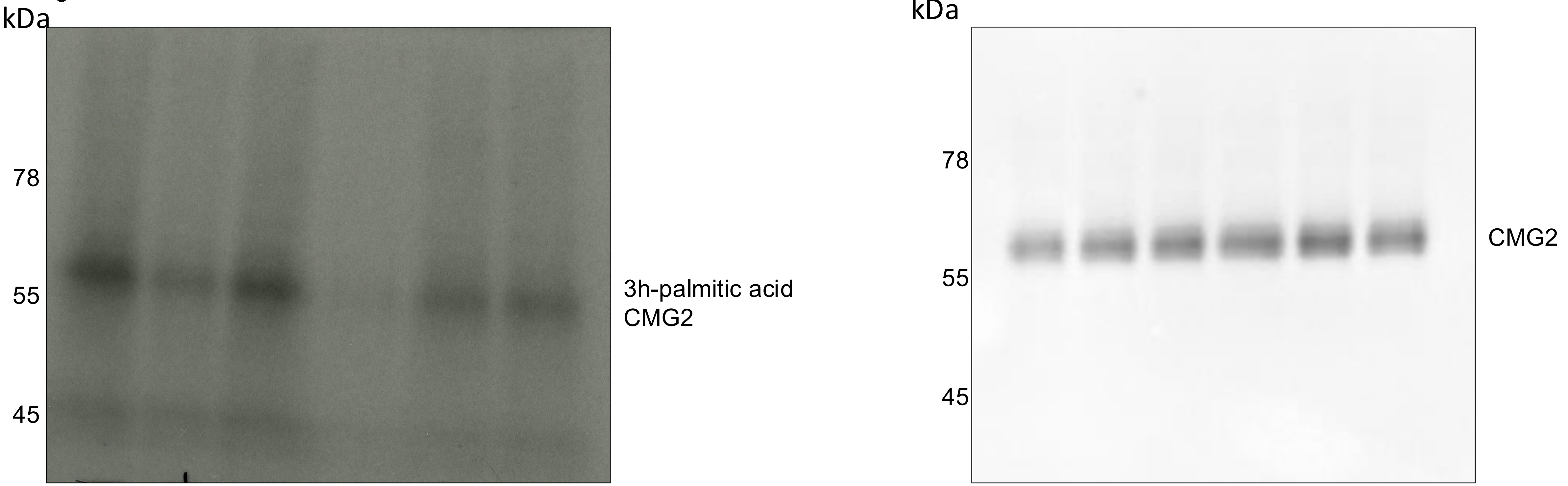

Figure 2d

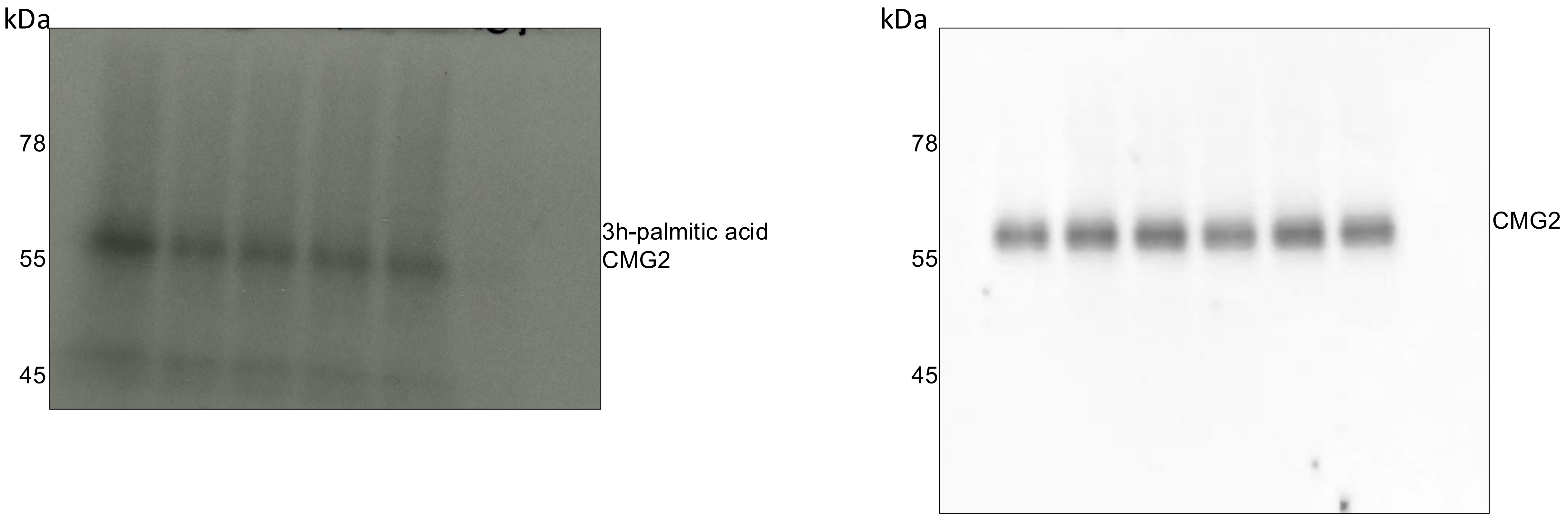

Figure 2e

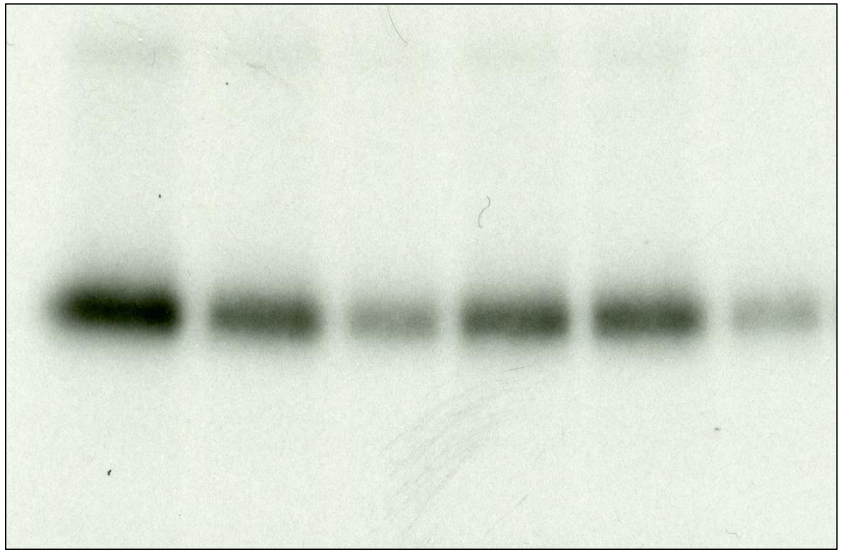

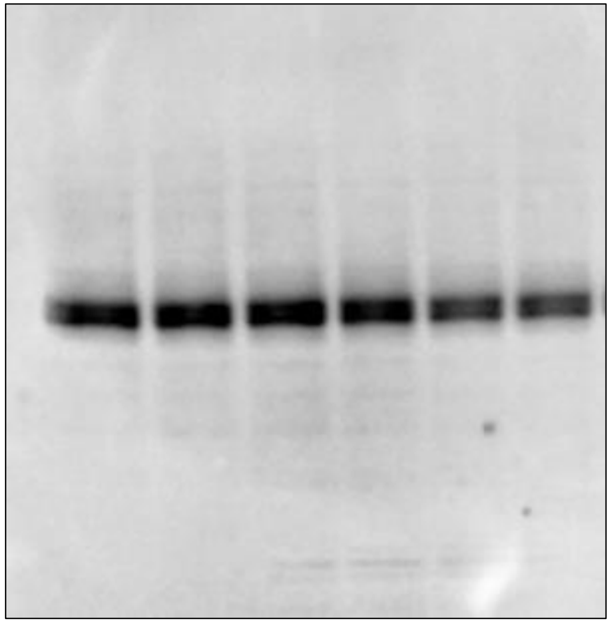

Extended Figure 2b

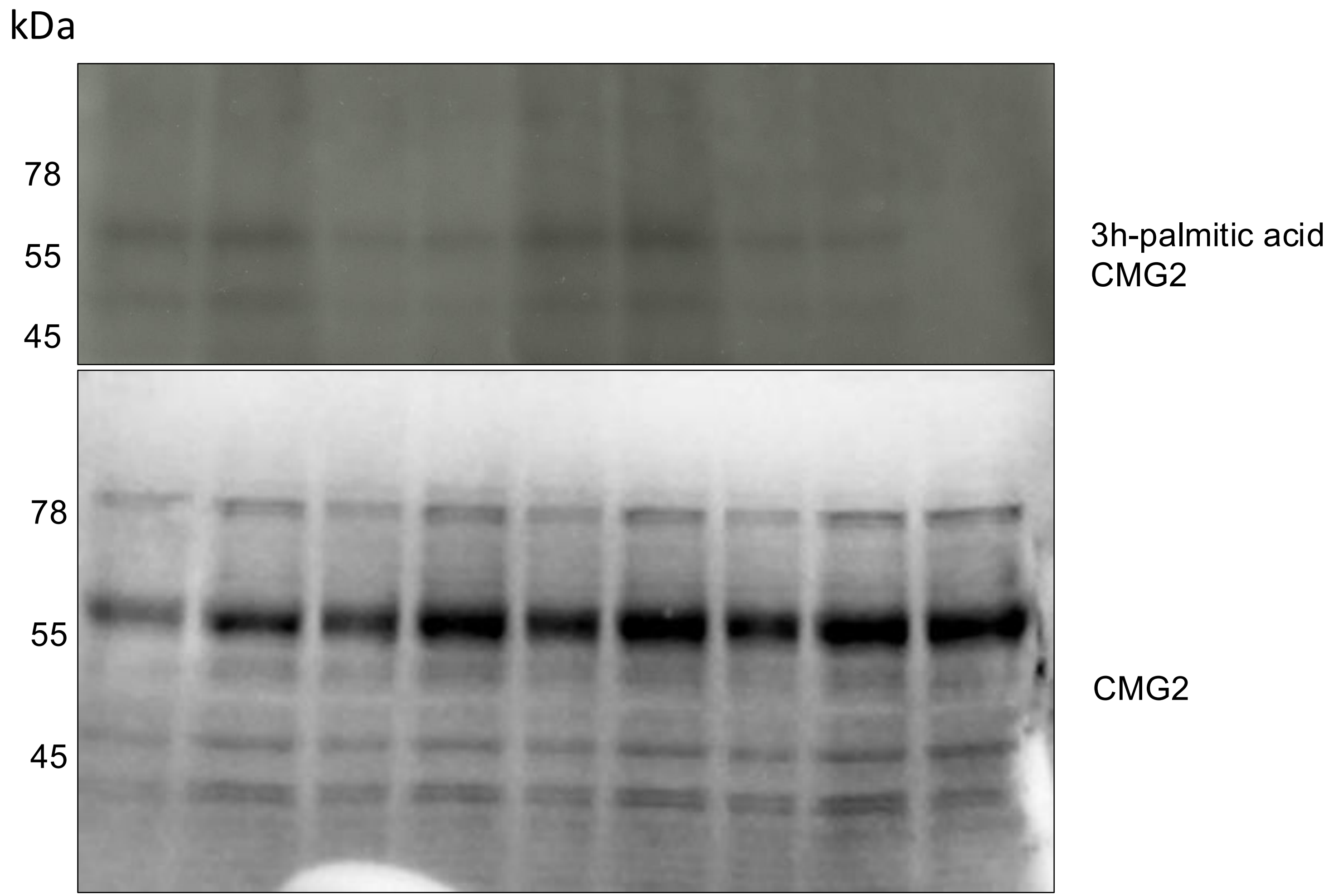

Extended Figure 2d

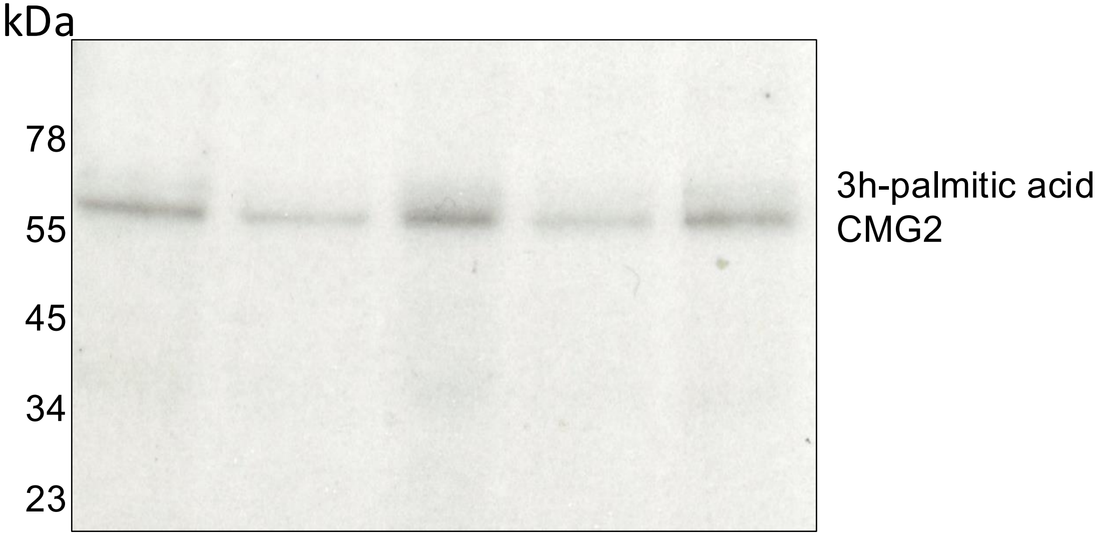

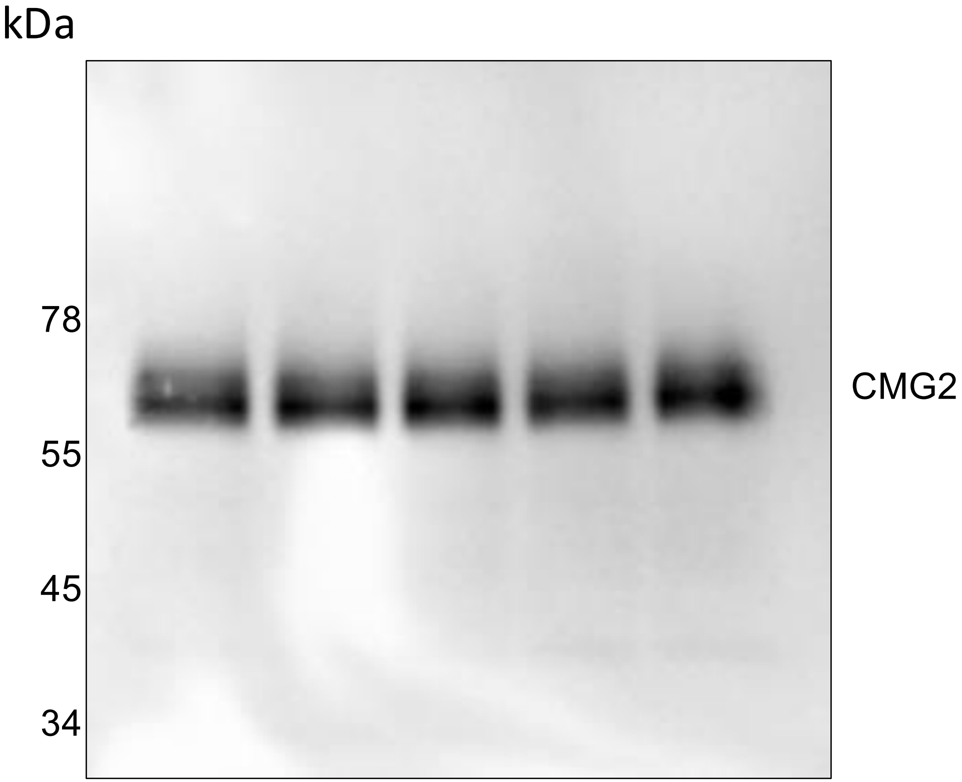

Figure 3d

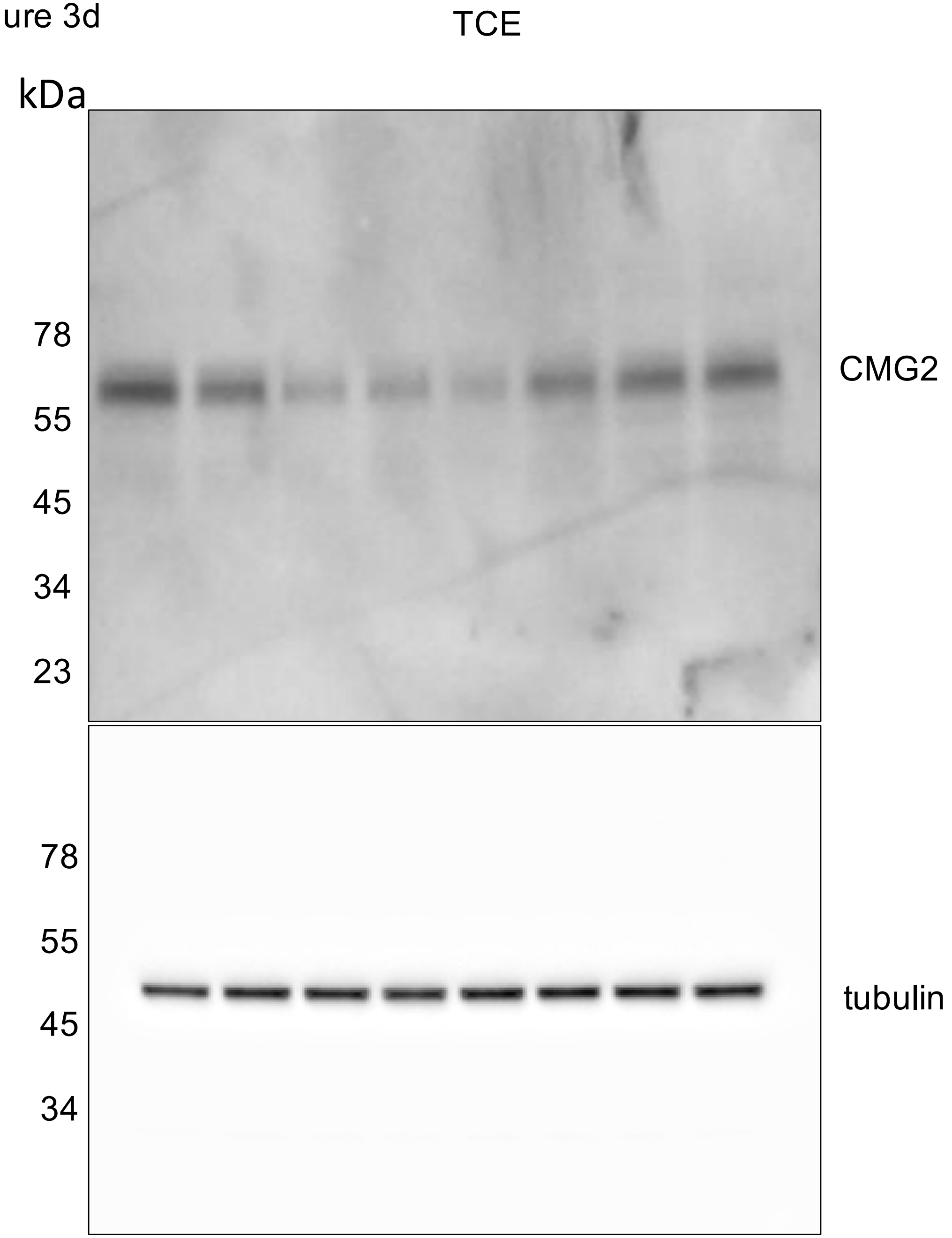

Figure 3e

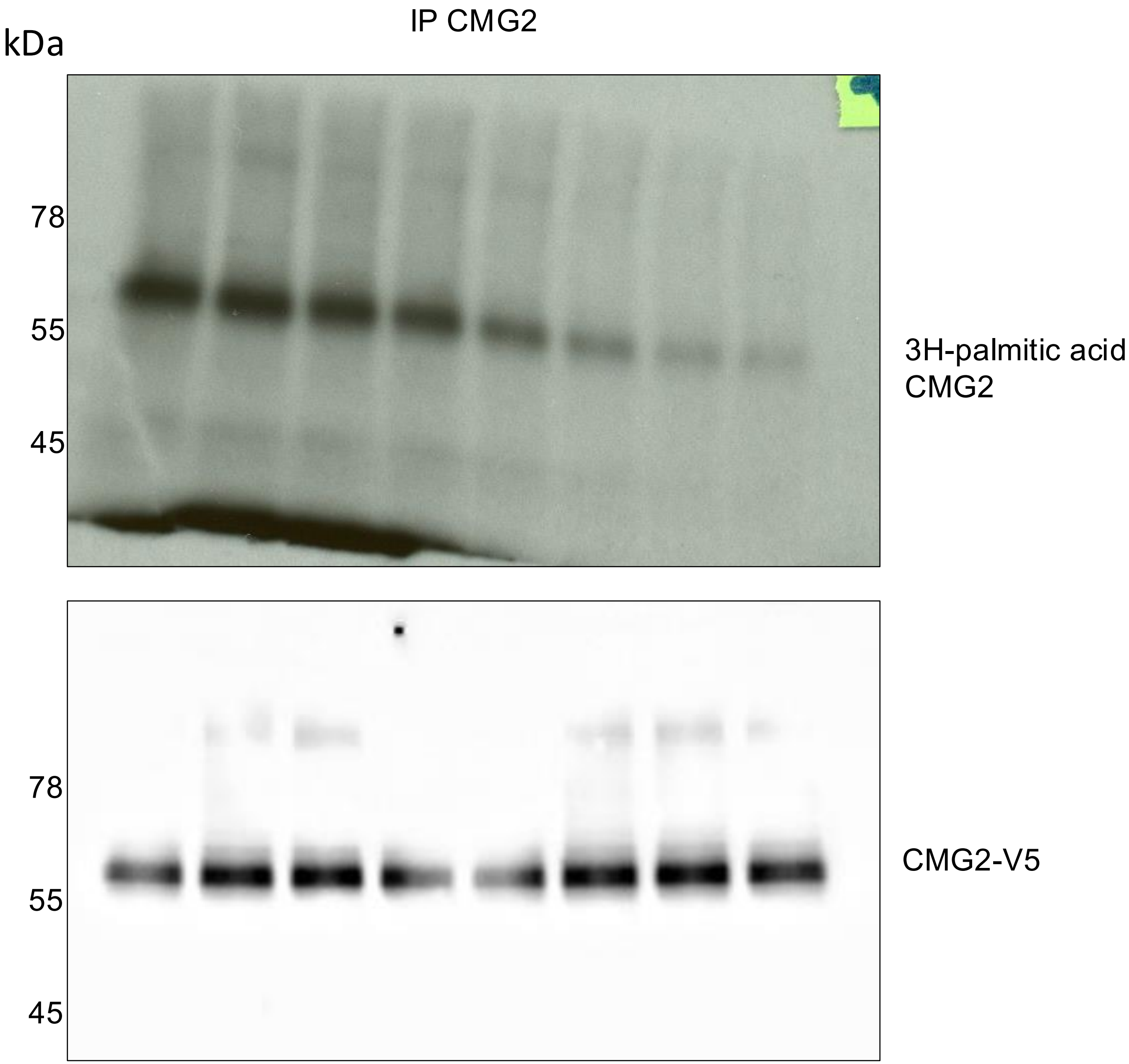

Extended figure 3c

IP CMG2

3H-palmitic acid
CMG2

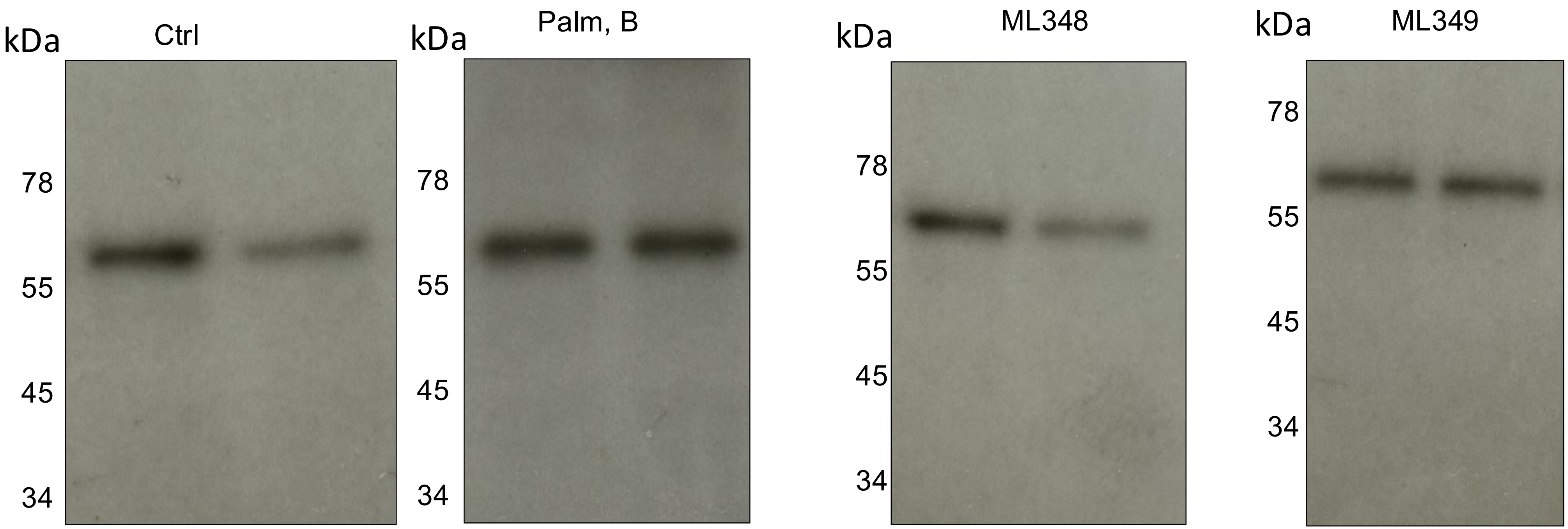

Extended figure 3d

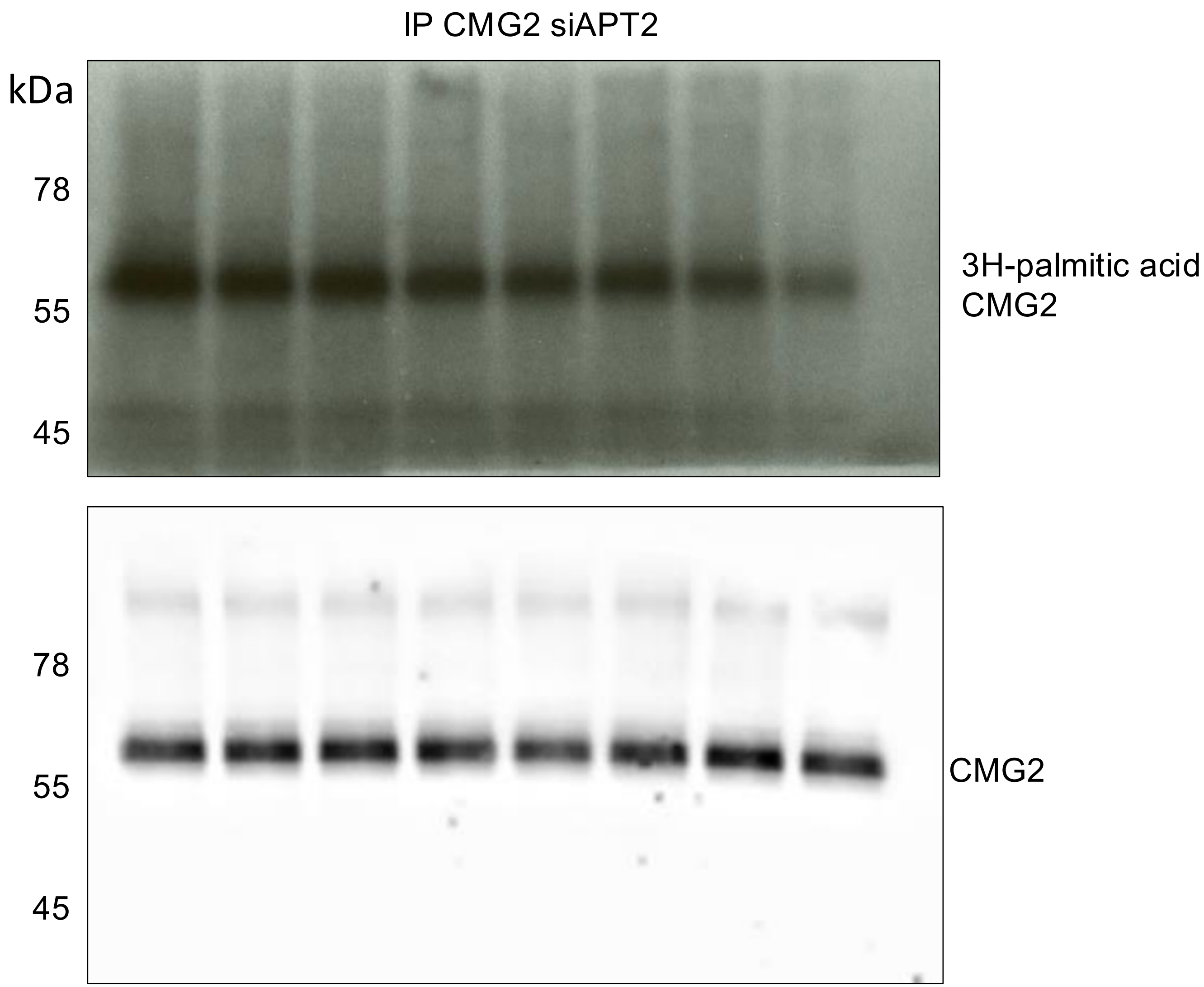

Extended figure 3g

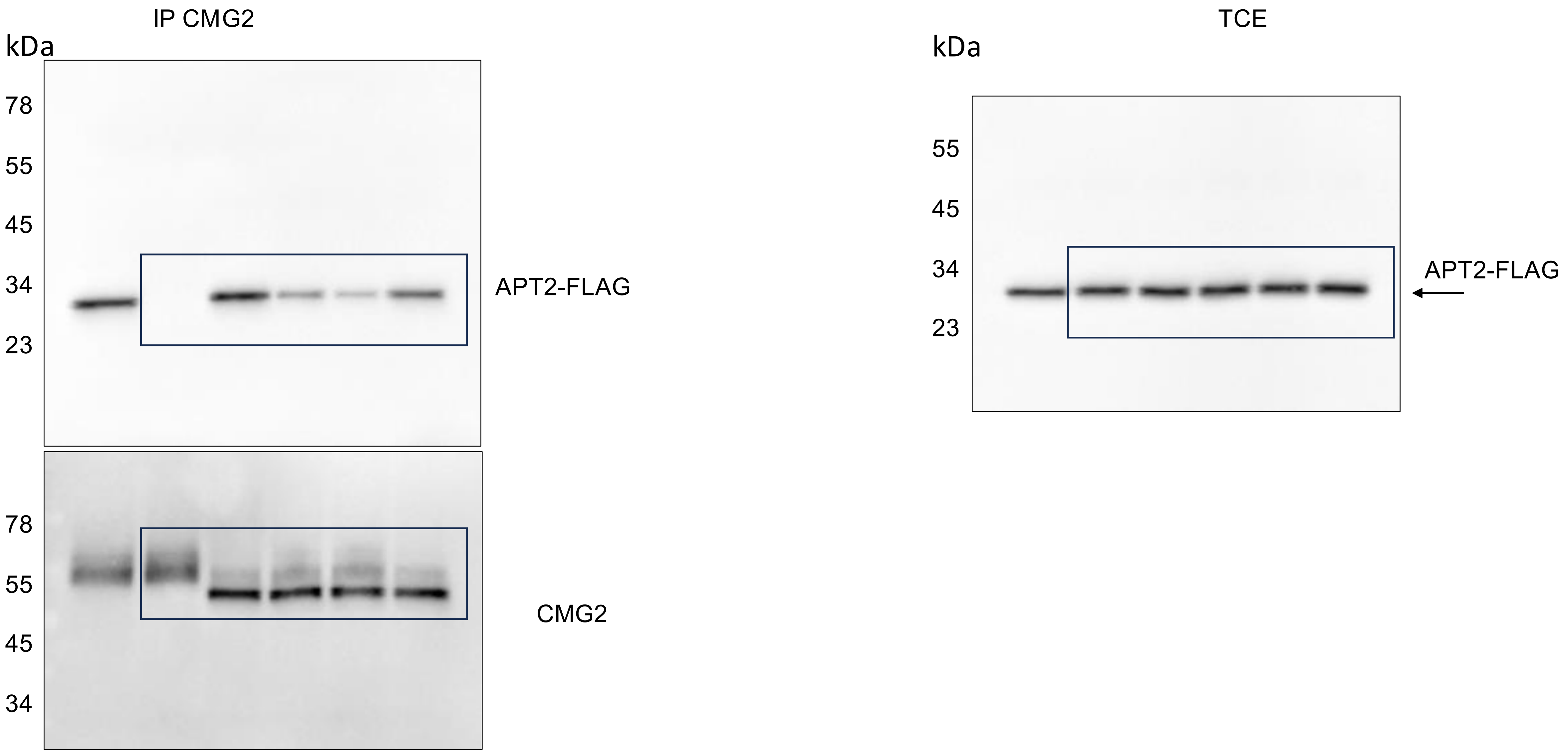

Extended figure 3h

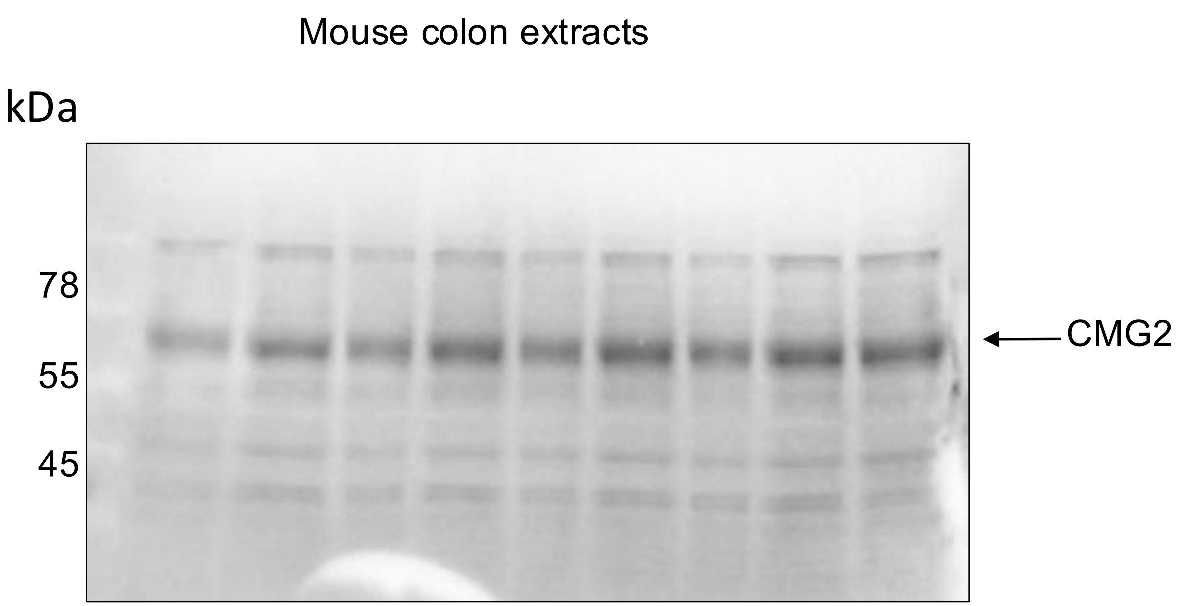

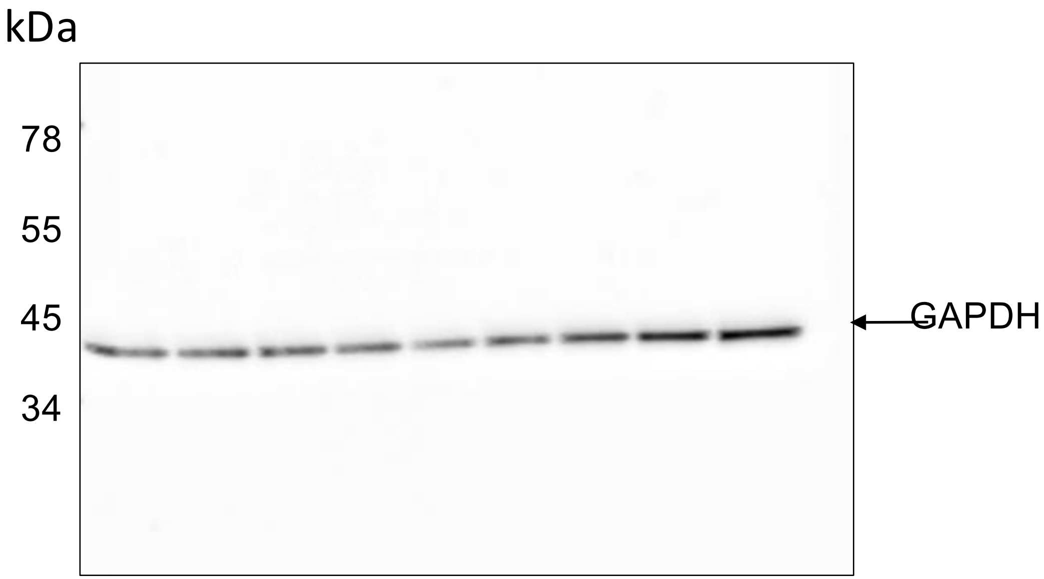

Figure 4c

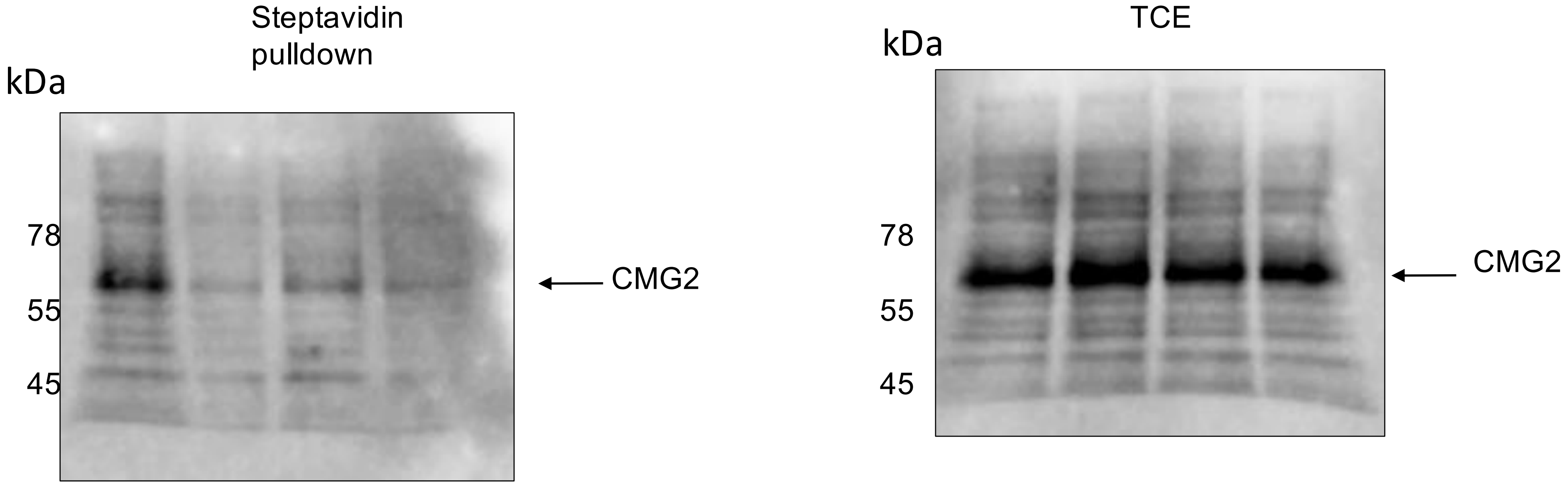

Figure 4e

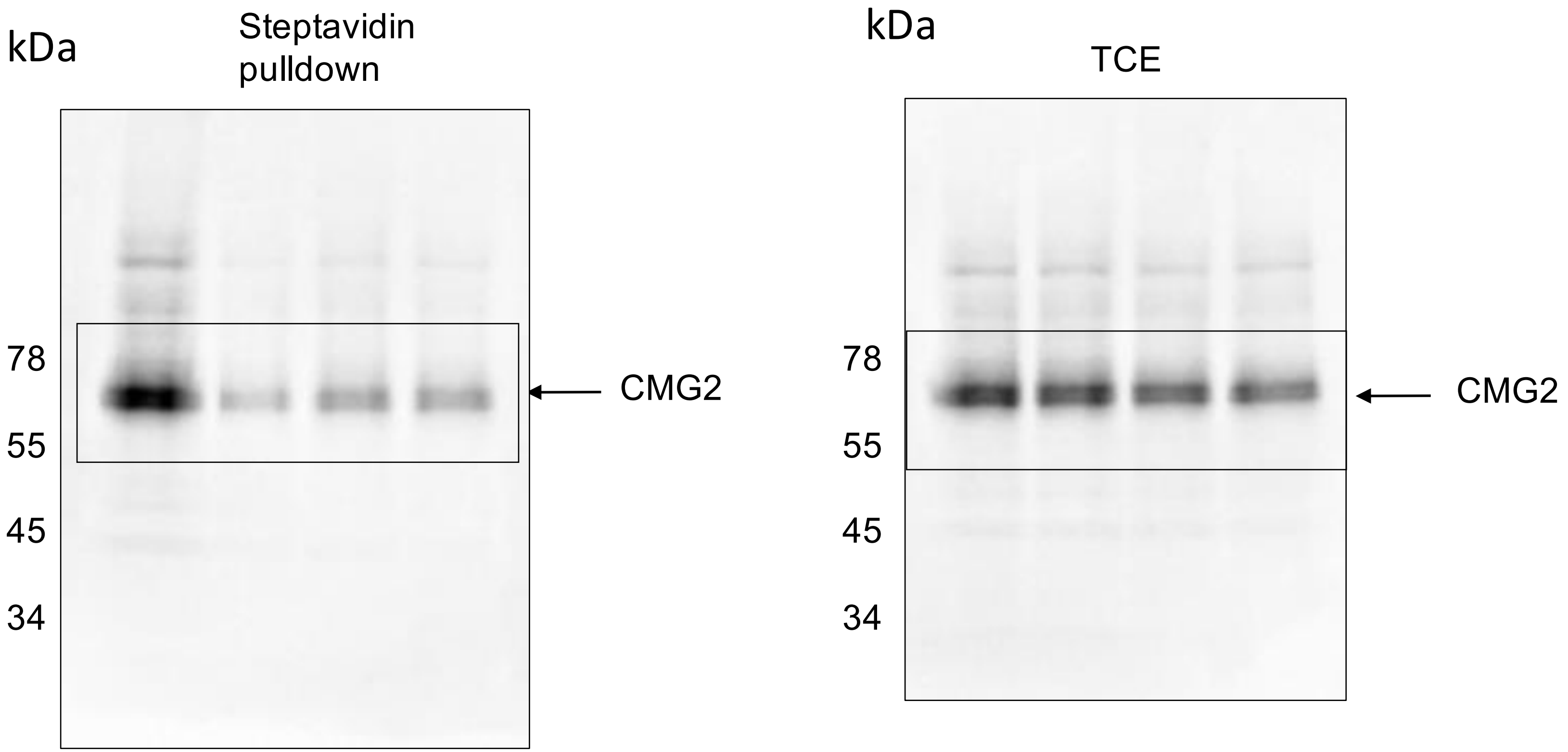

kDa
Steptavidin pulldown
78
55
45
34
CMG2
kDa
TCE
78
55
45
34
CMG2

Figure 4f

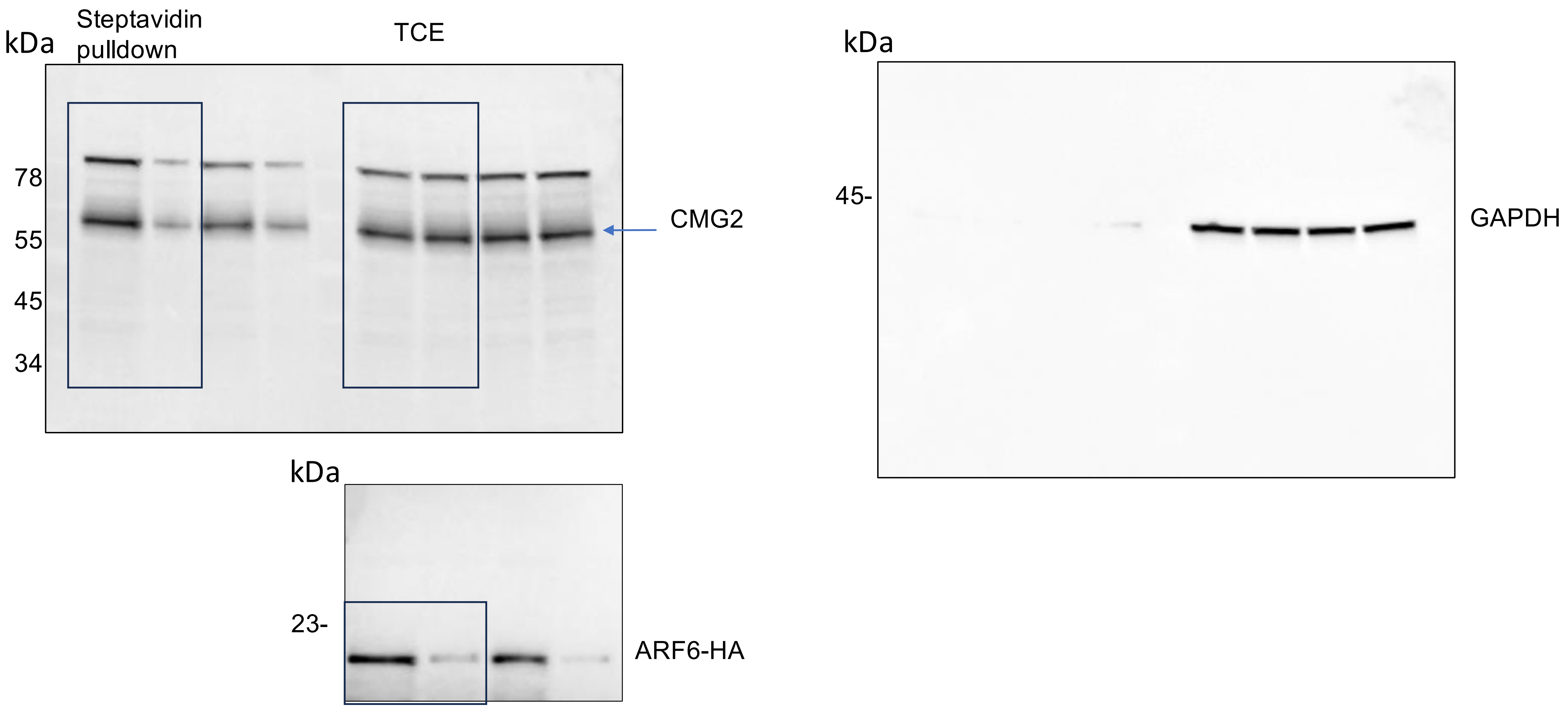
kDa
Steptavidin pulldown
TCE
78
55
45
34
CMG2
kDa
23-
ARF6-HA
kDa
45-
GAPDH

Figure 4g

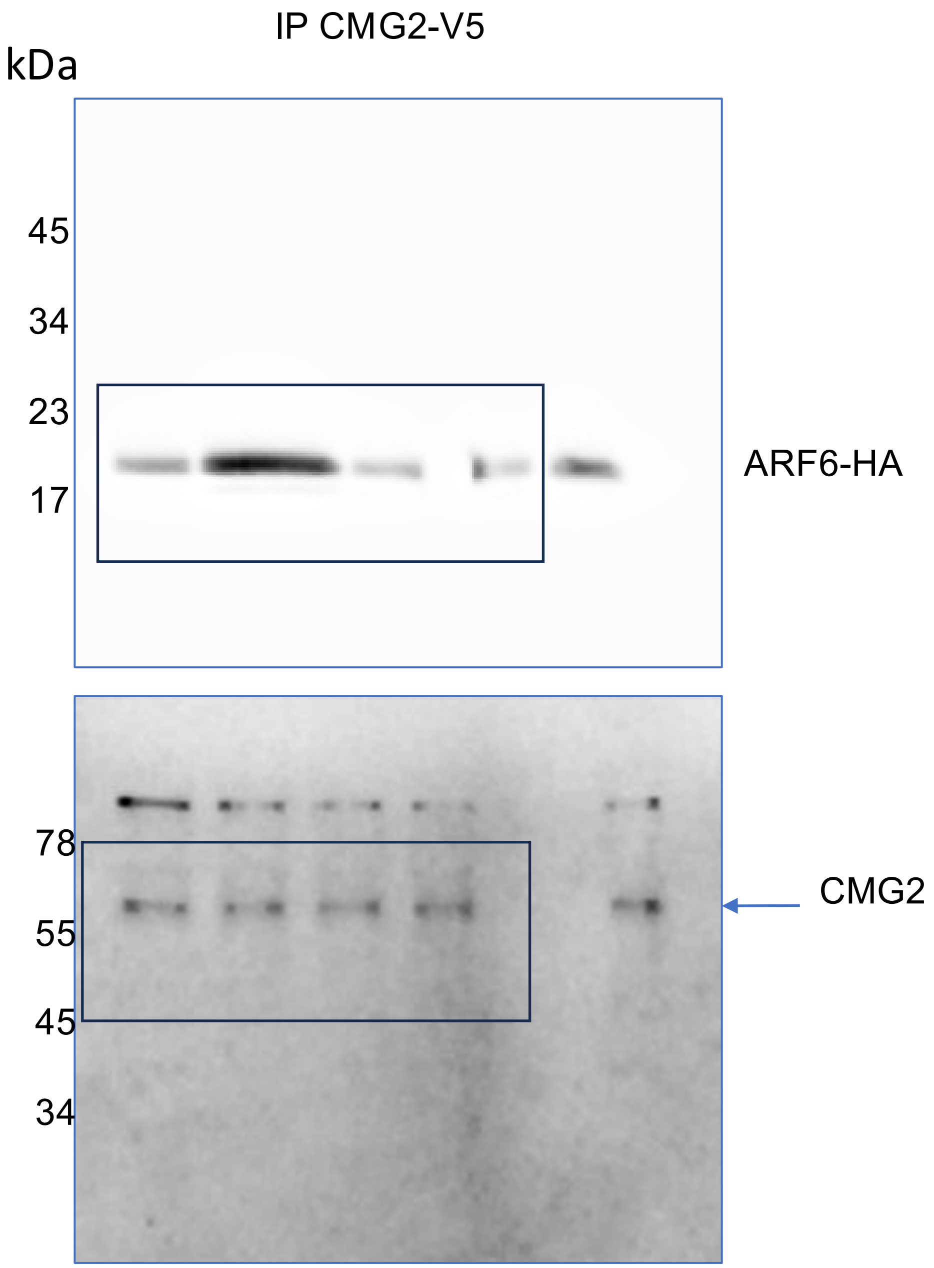

Extended Data Figure 4a

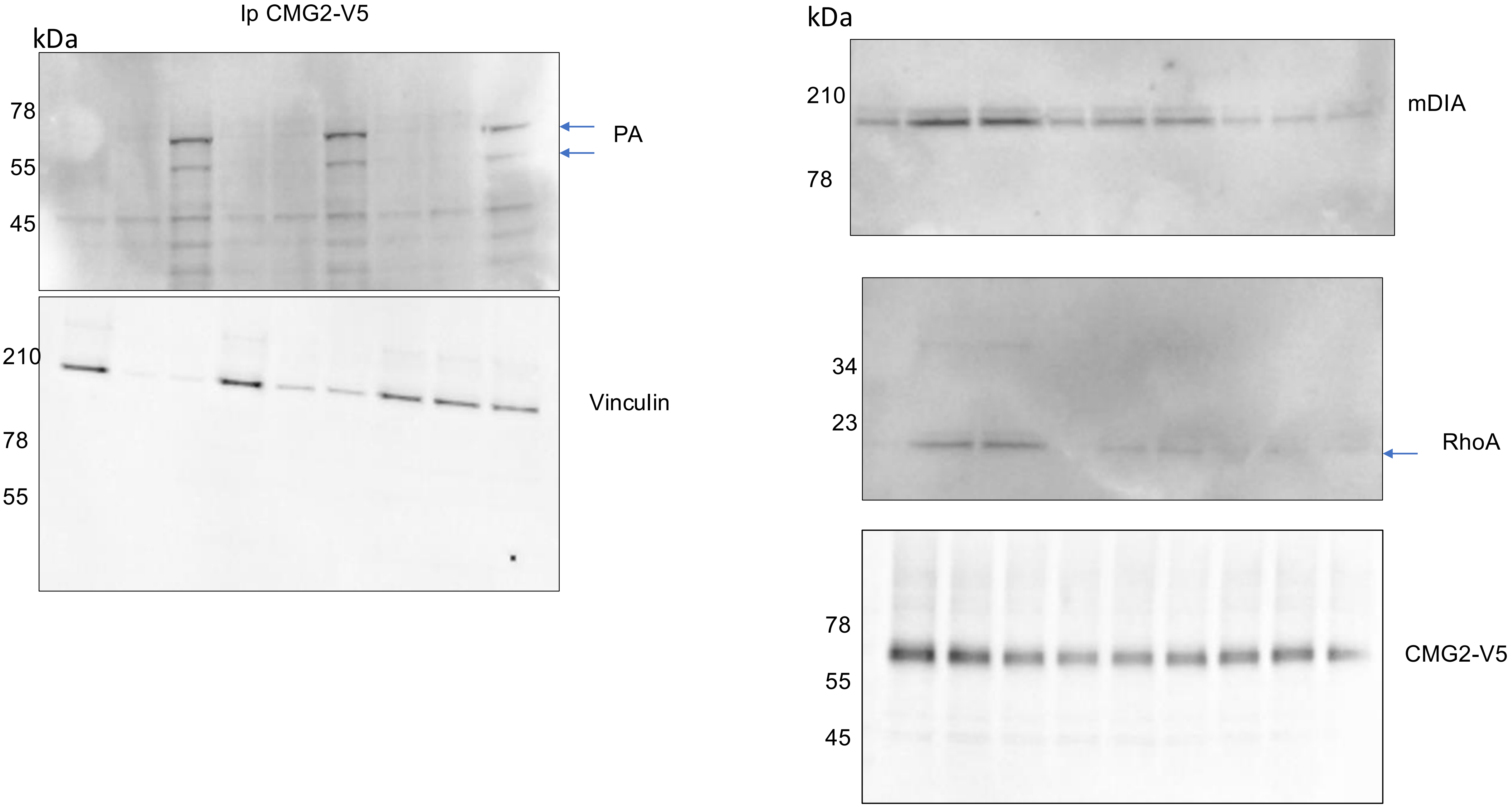

Extended Data Figure 4b

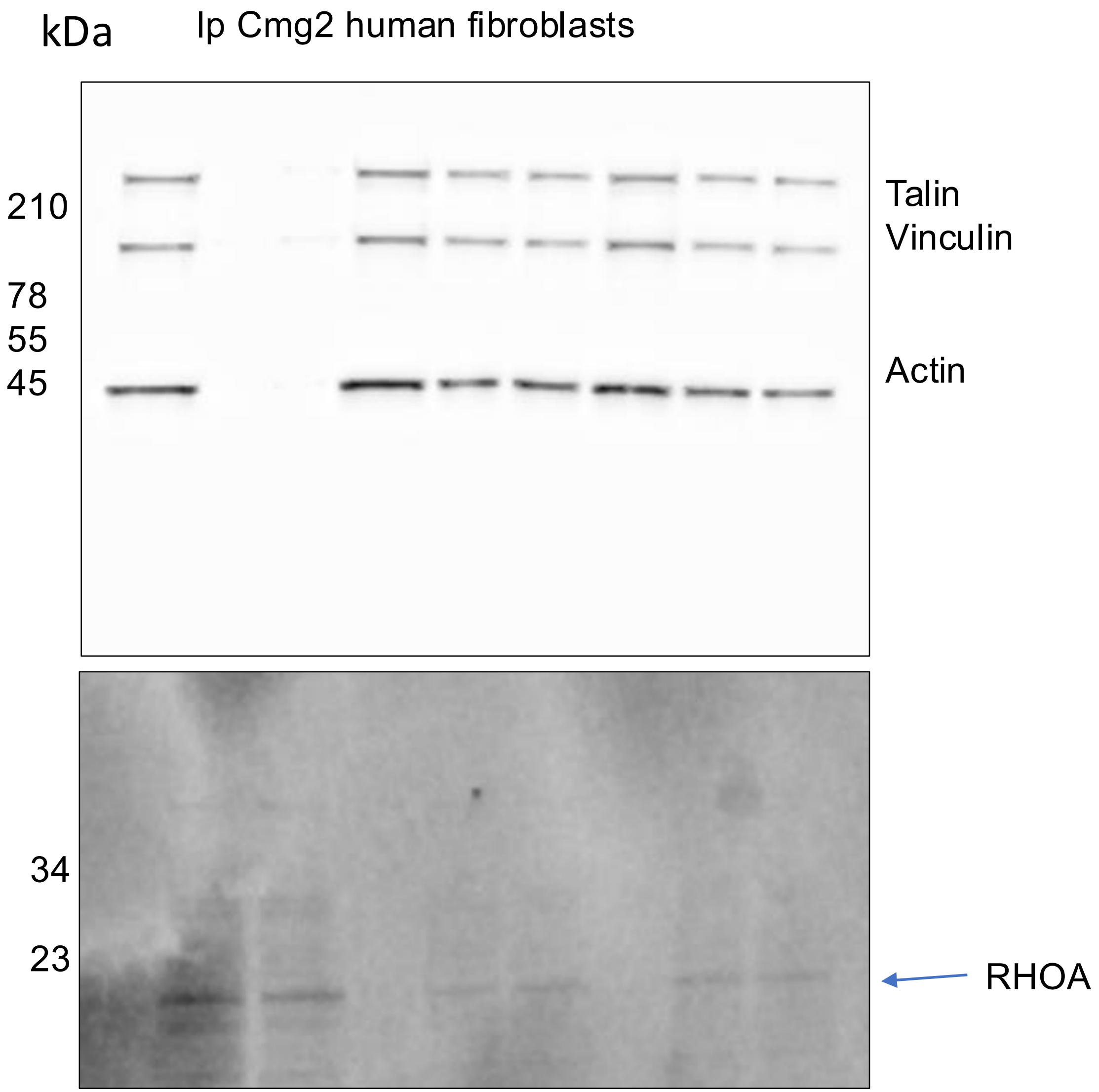

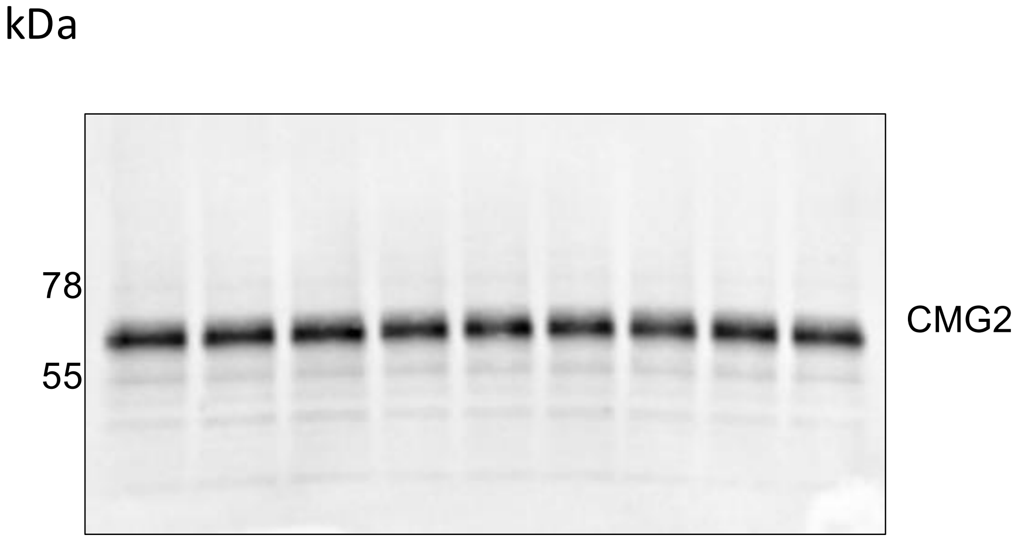

Extended Data Figure 4c

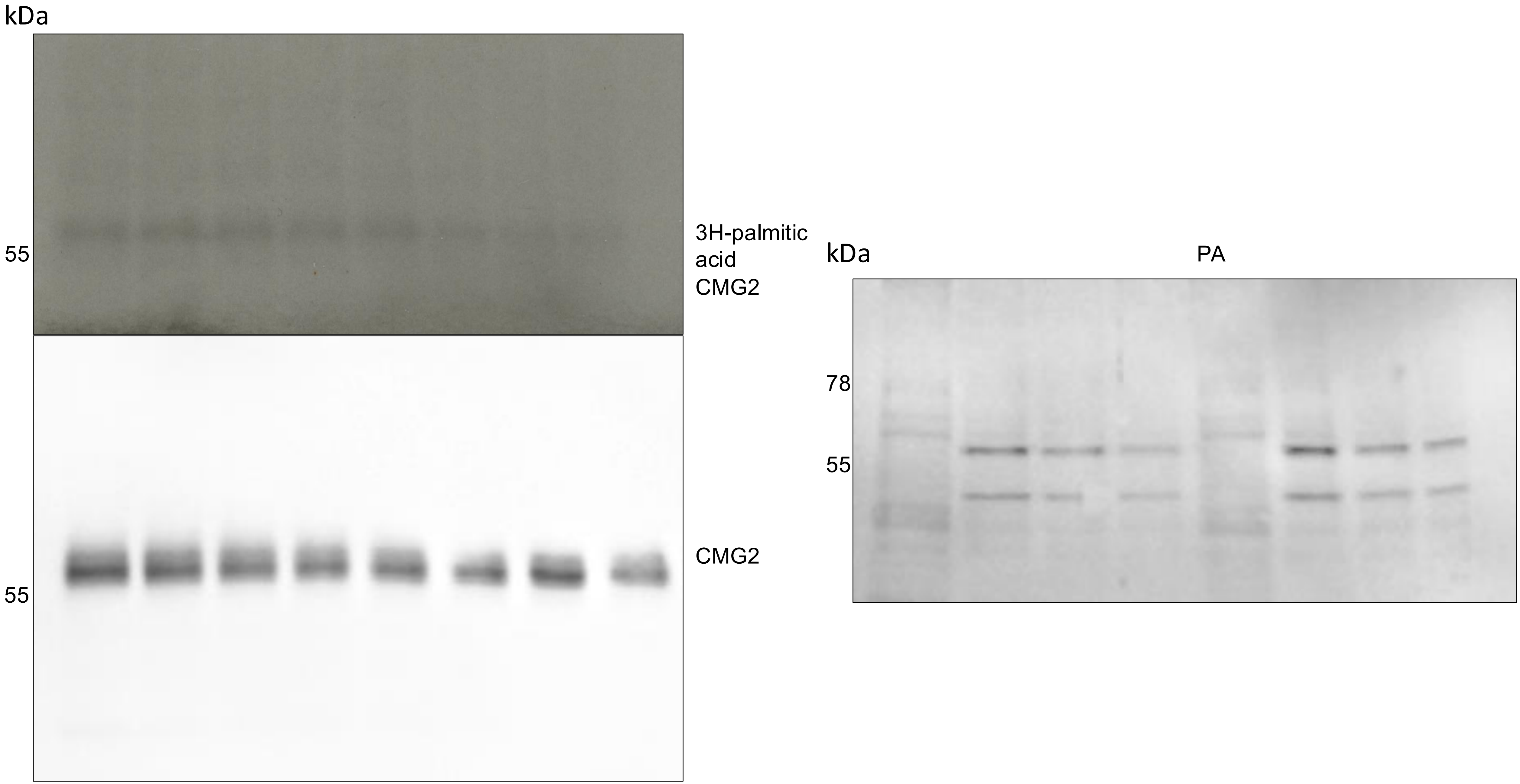

Figure 5b

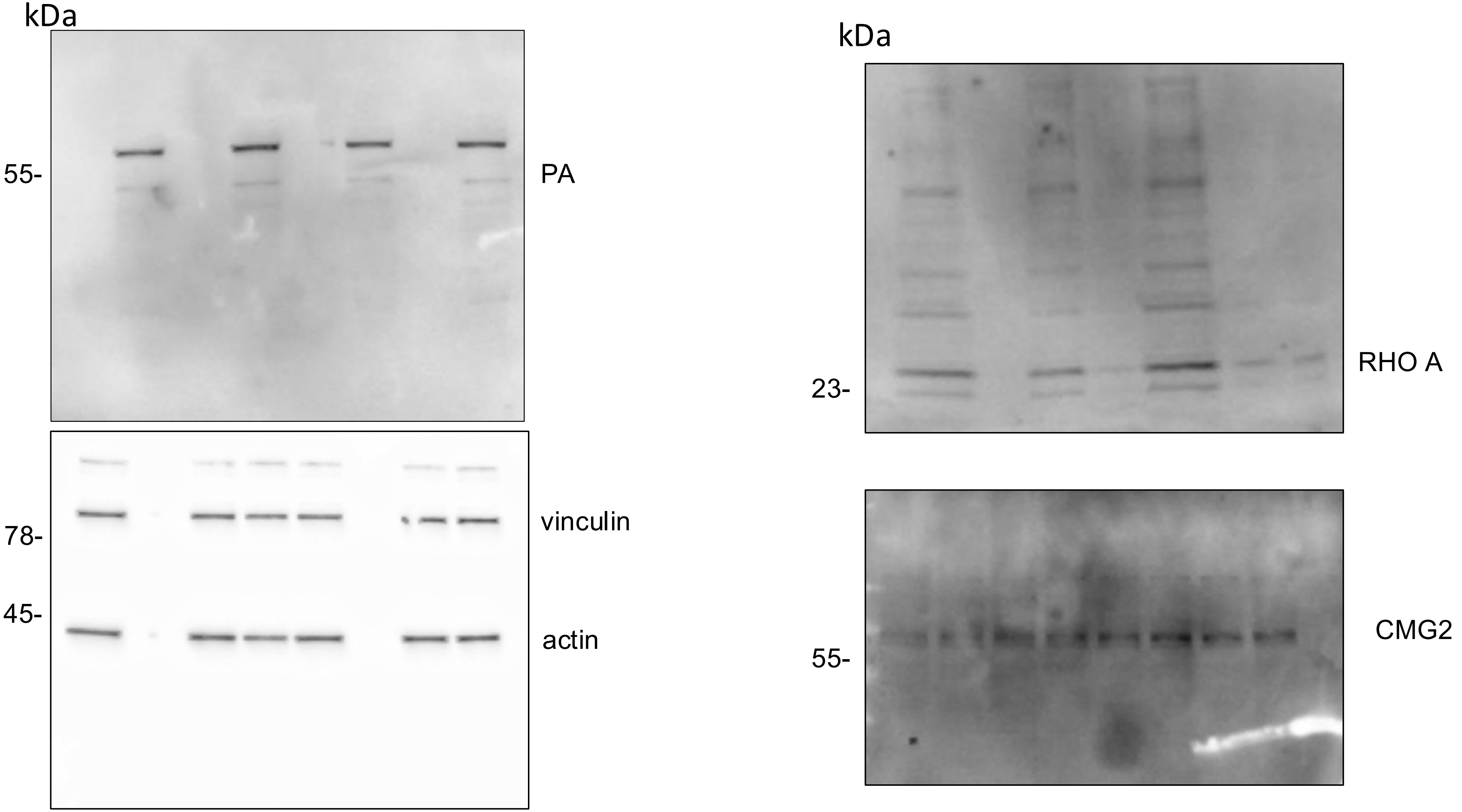

Figure 5d

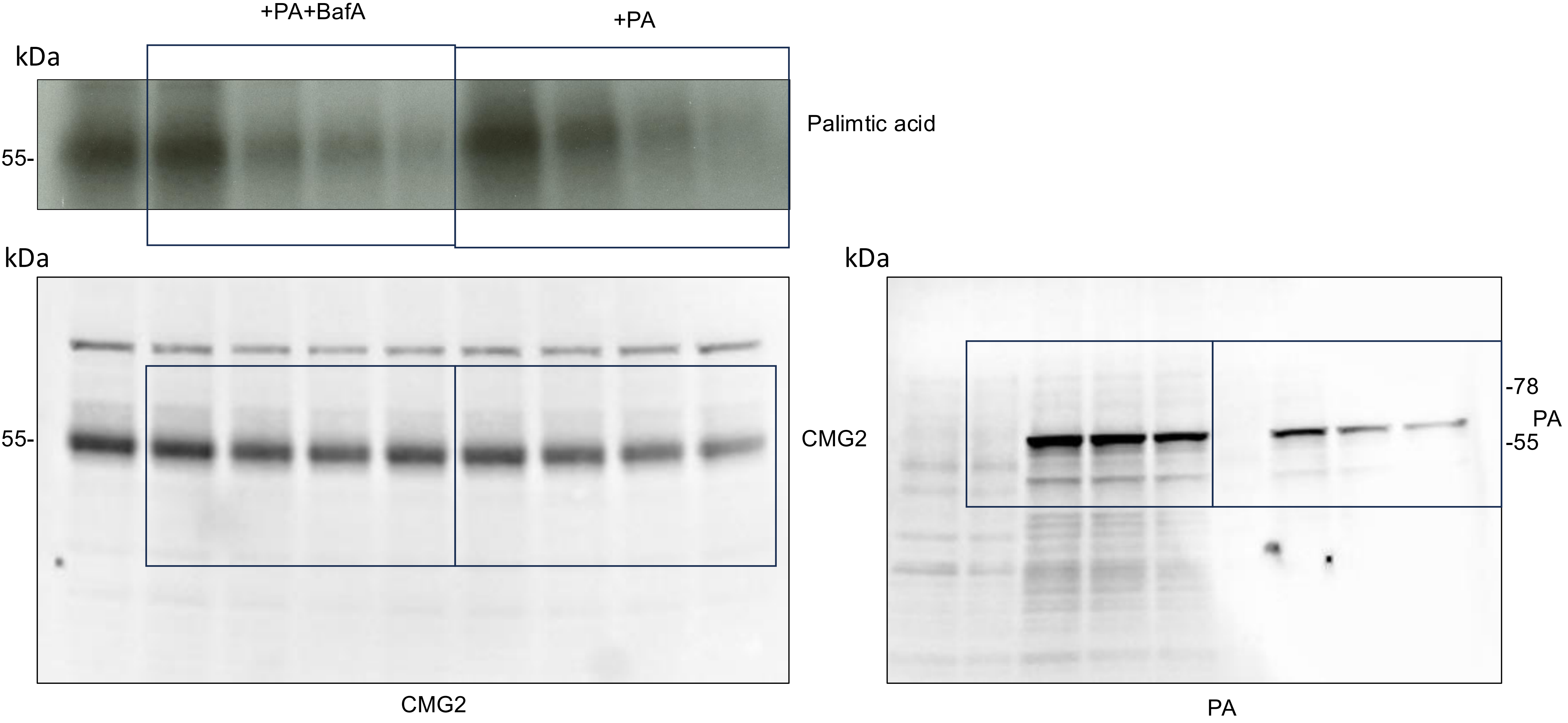

Extended Data Figure 5a

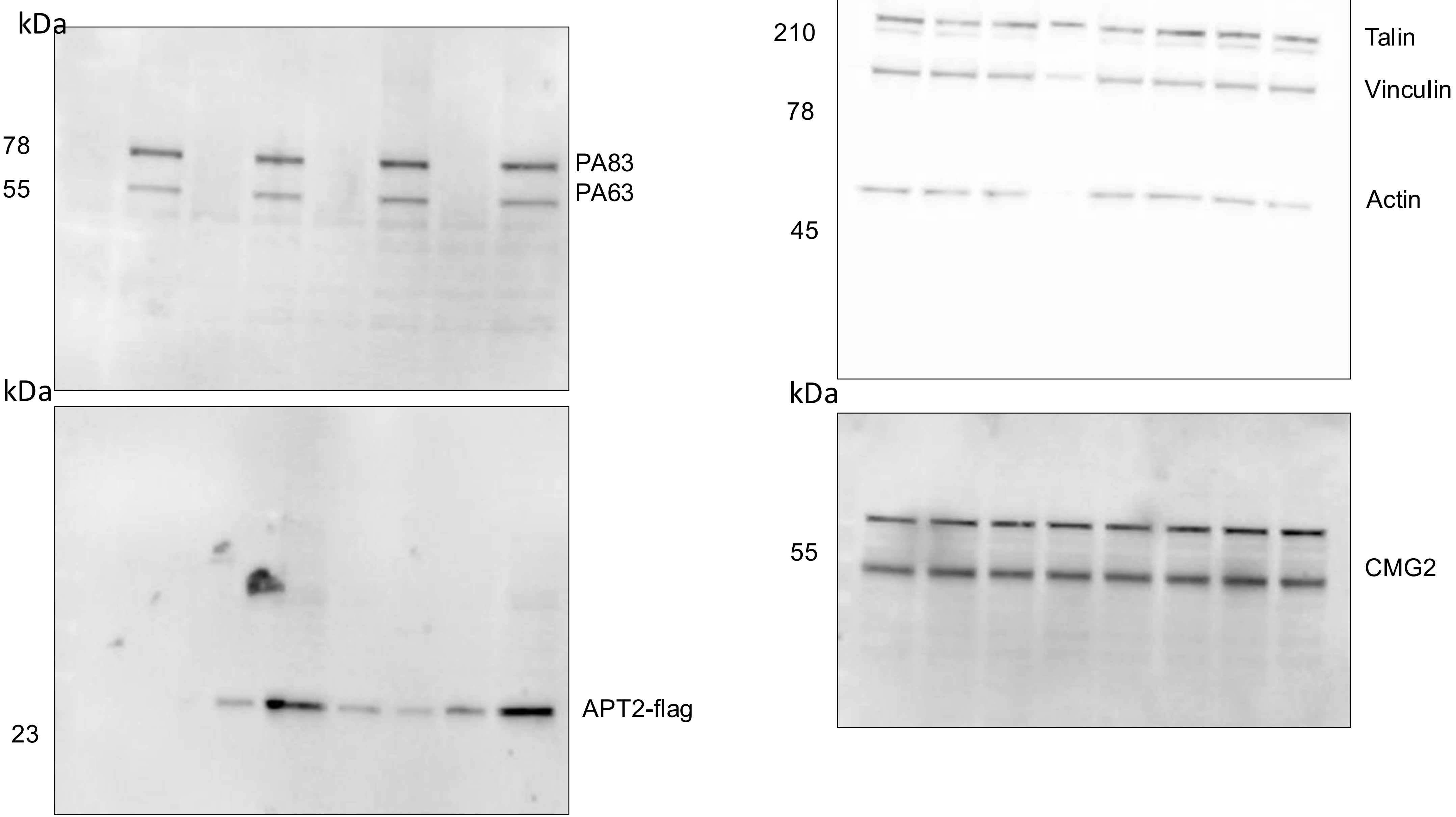

Extended Data Figure 5b

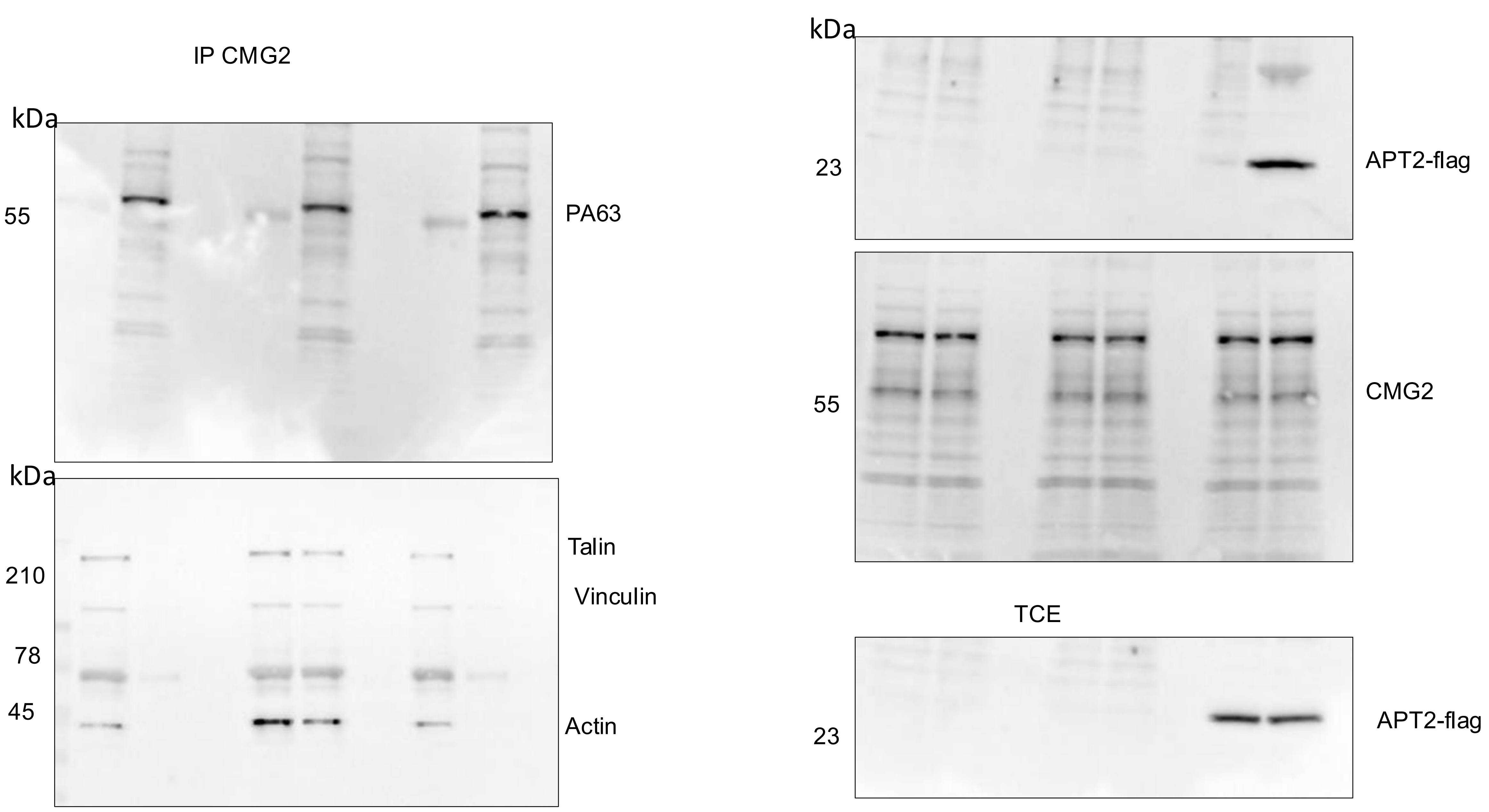

Extended Data Figure 5c

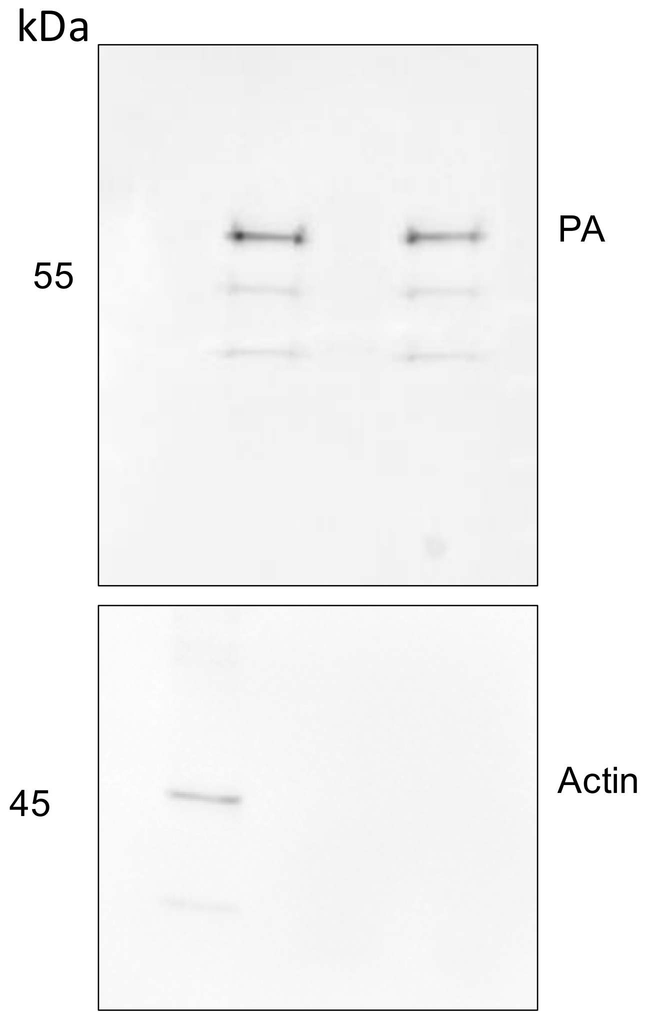

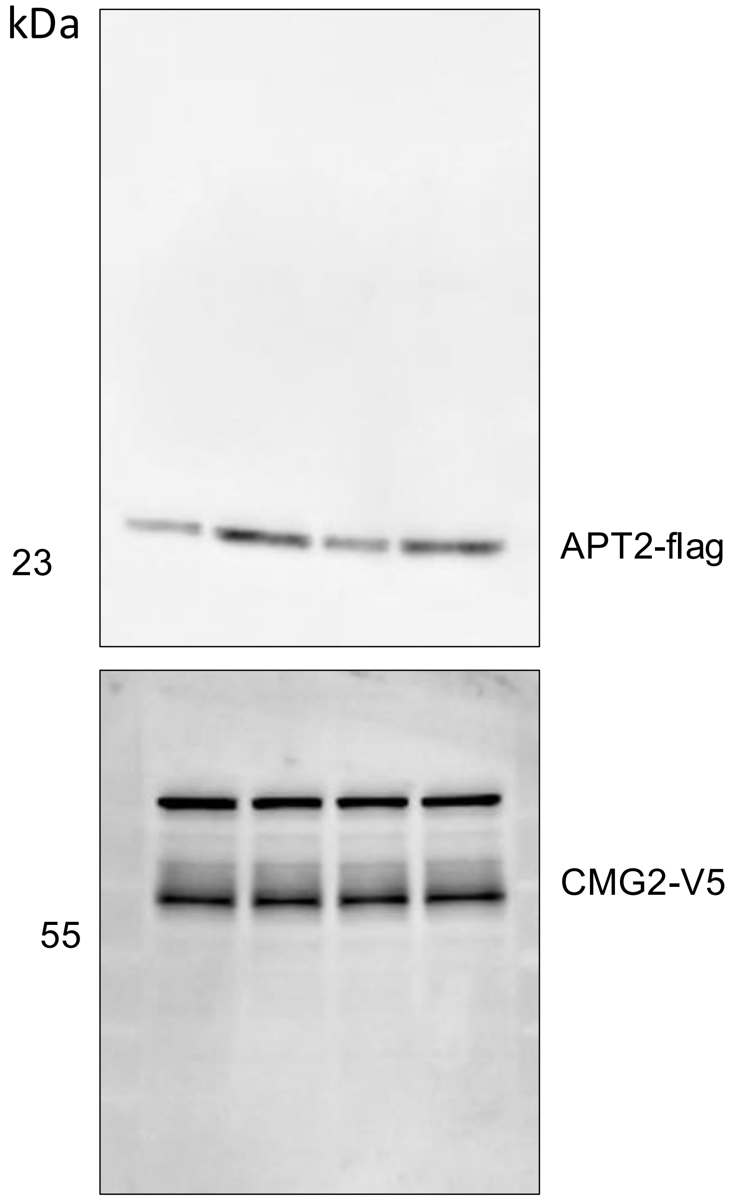

Extended Data Figure 5d

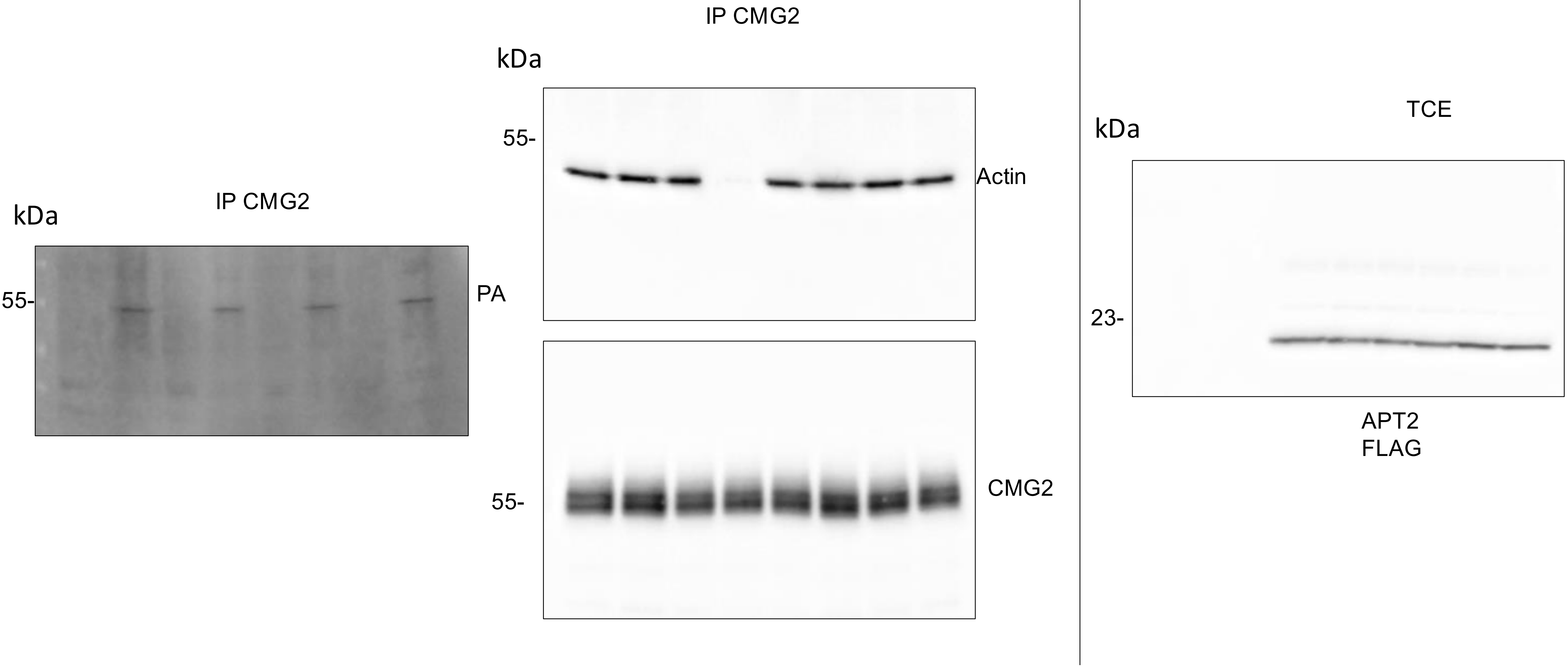

Extended Data Figure 5e

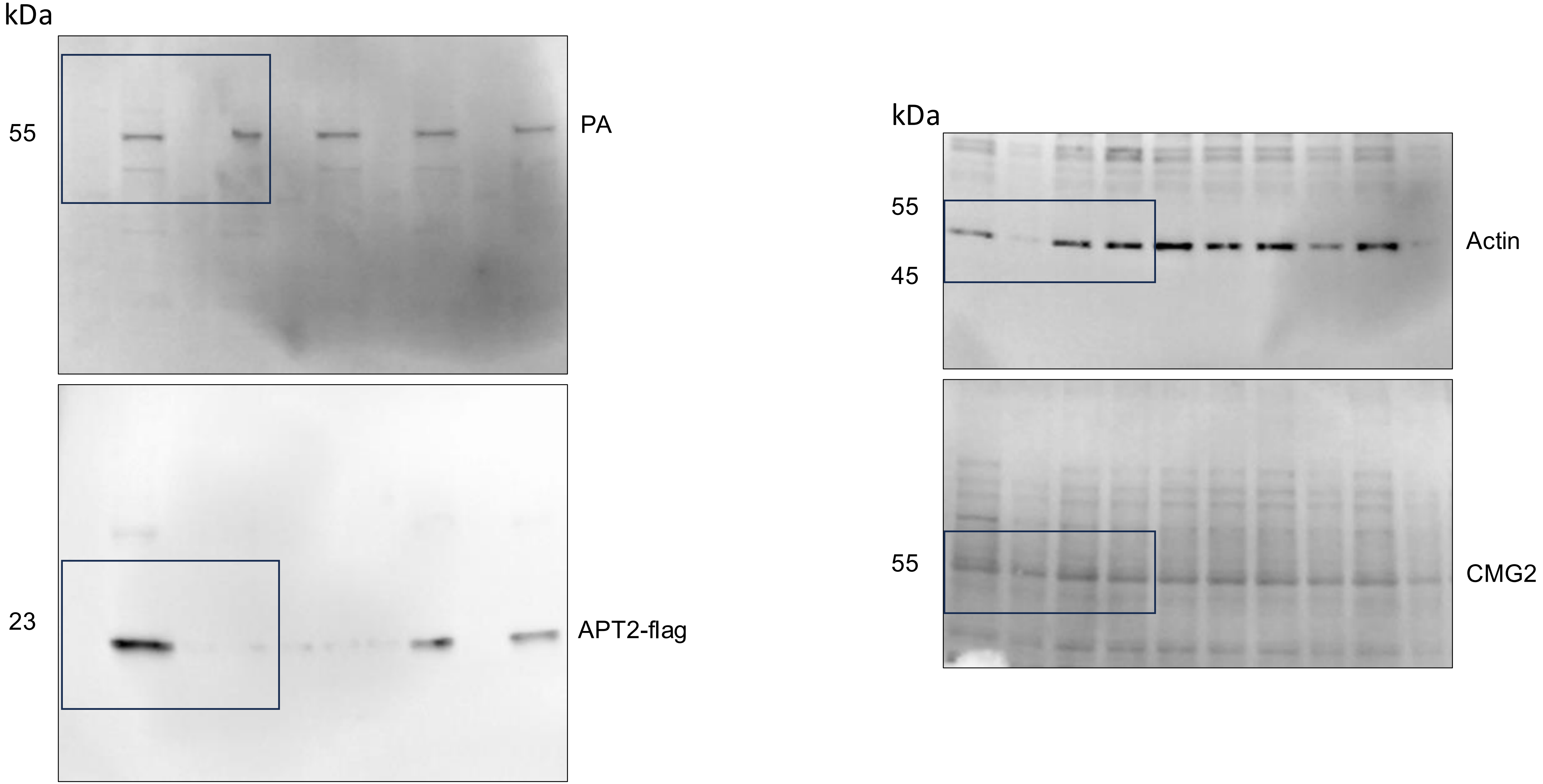

Extended Data Figure 5g

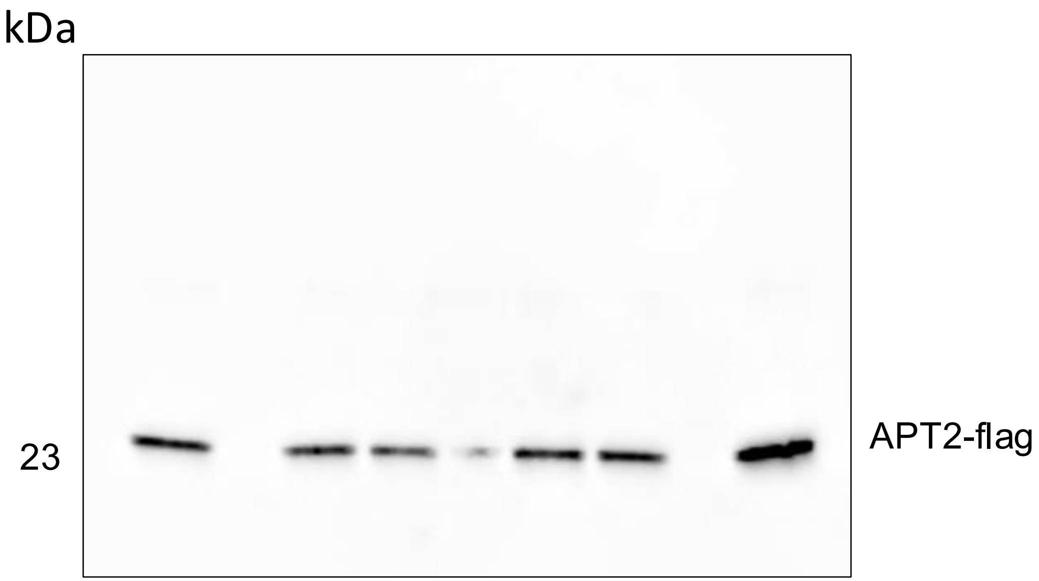

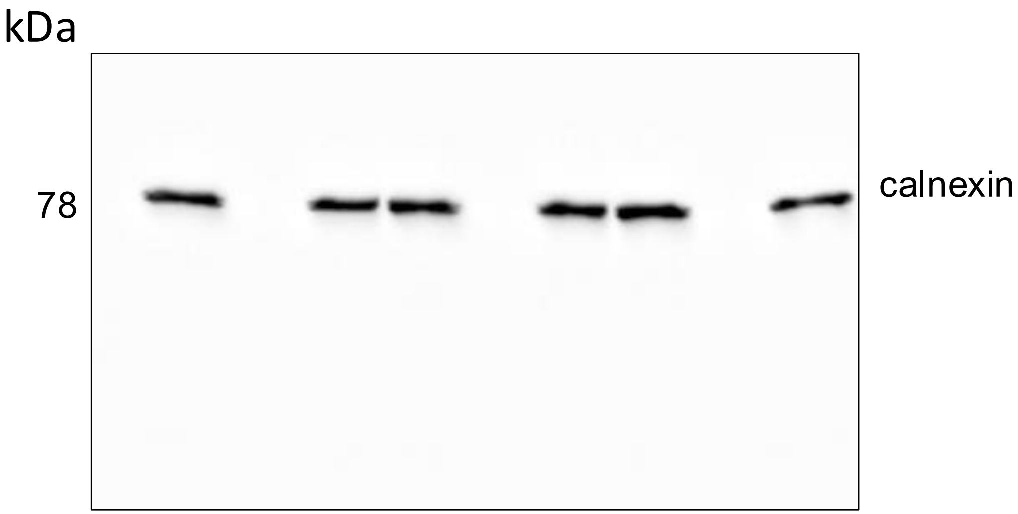

Extended Data Figure 5h

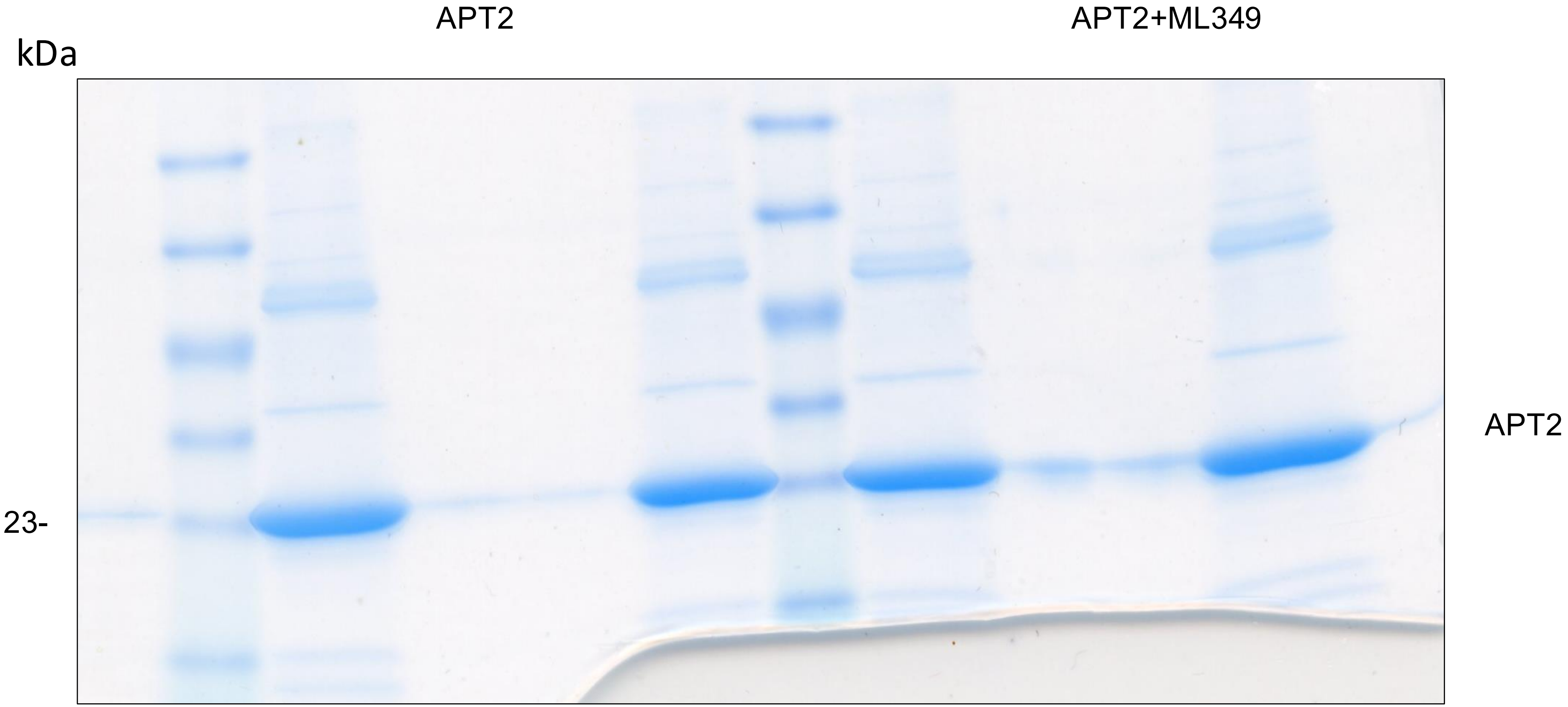

Figure 6a

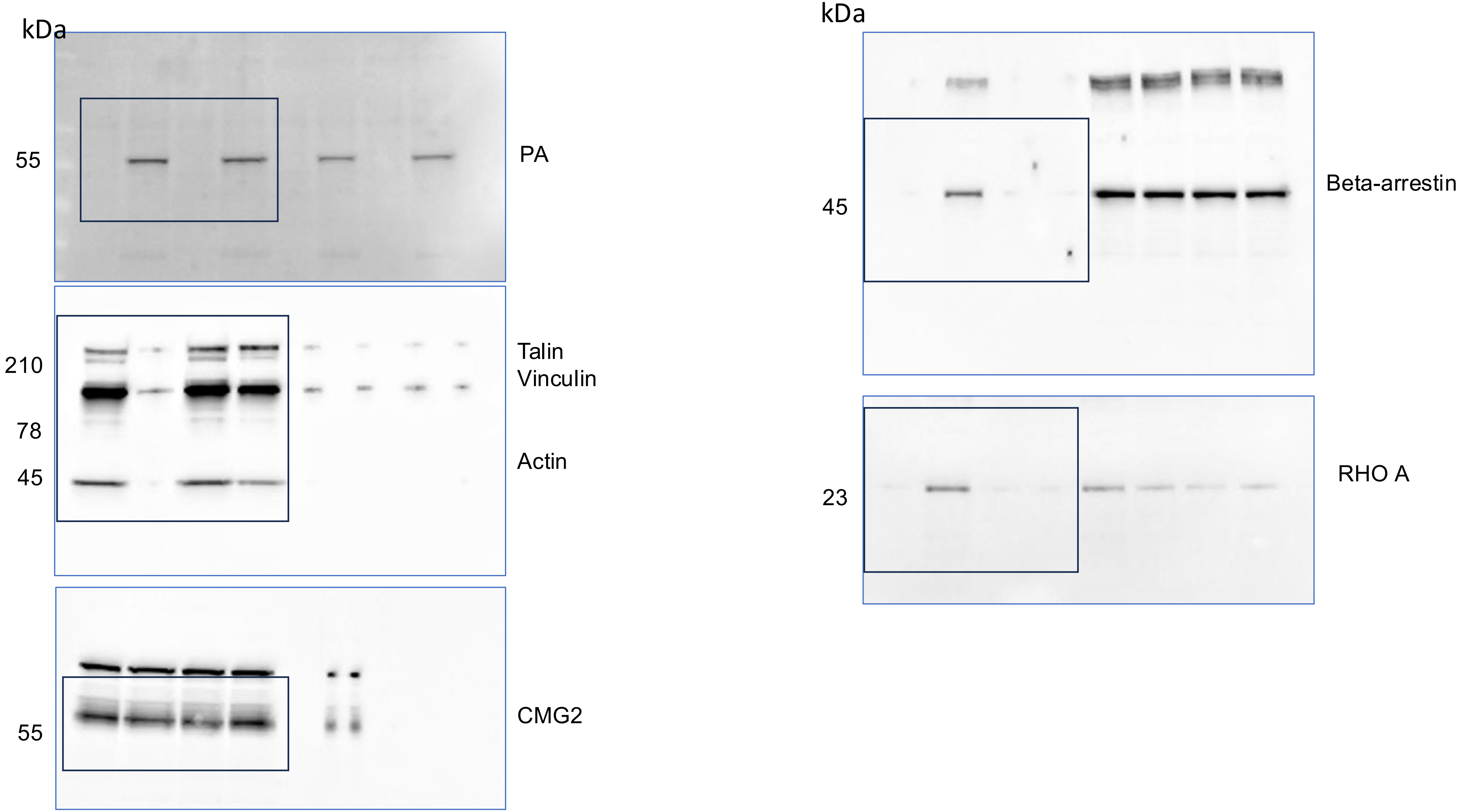

kDa
55
PA
210
78
45
Talin
Vinculin
Actin
55
CMG2
kDa
45
Beta-arrestin
23
RHO A

Figure 6b

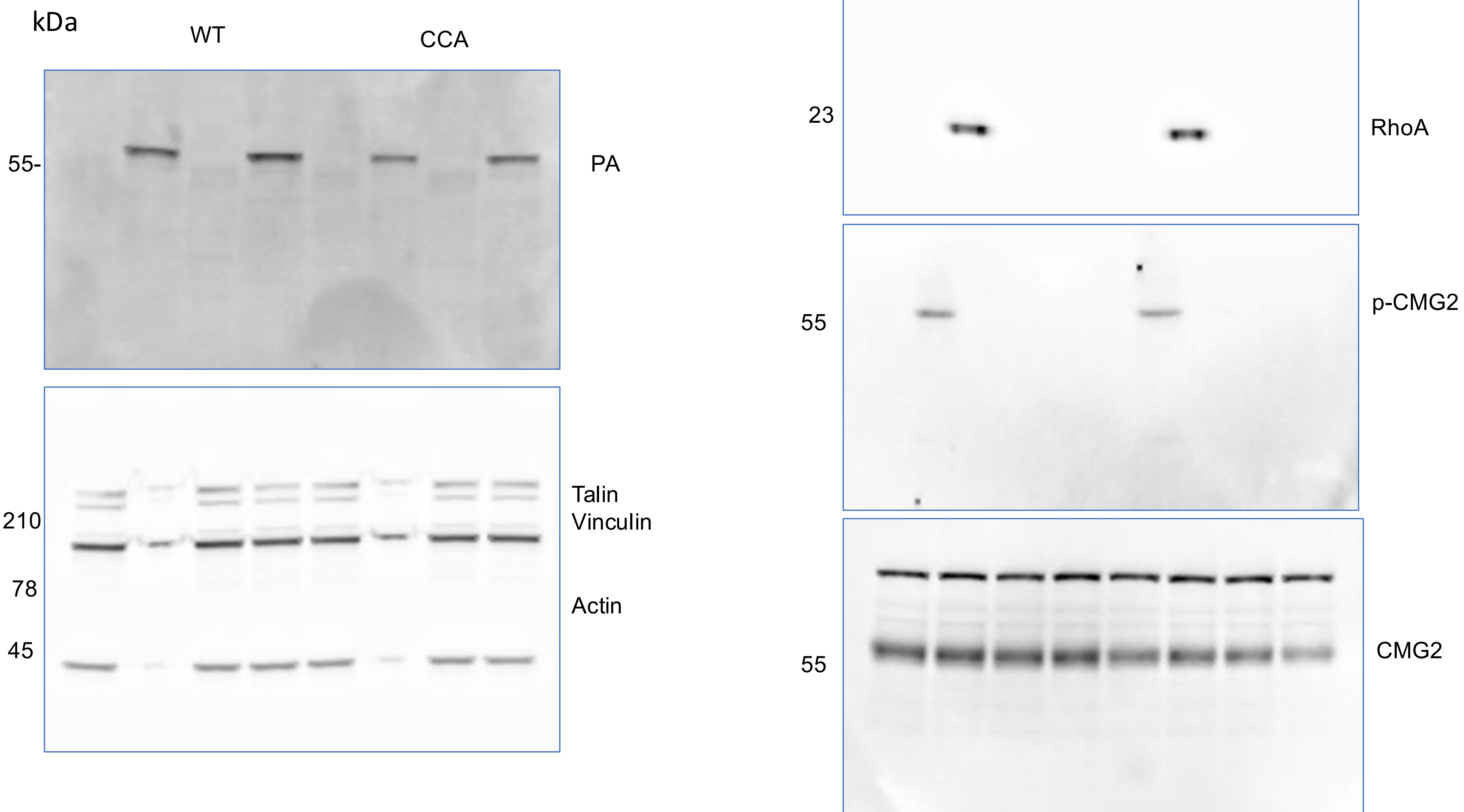
kDa
WT
CCA
55-
PA
210
78
45
Talin
Vinculin
Actin
kDa
23
RhoA
55
p-CMG2
55
CMG2

Figure 6b

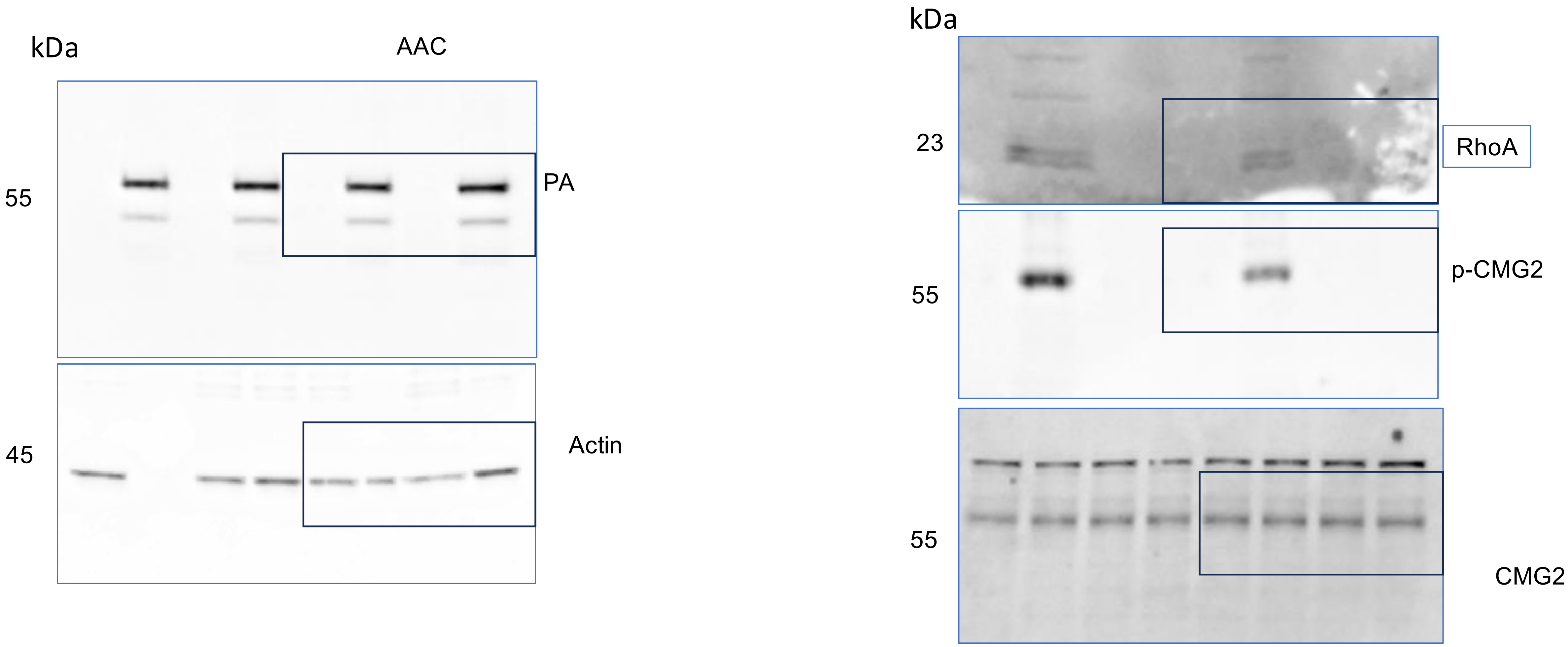

Figure 6d

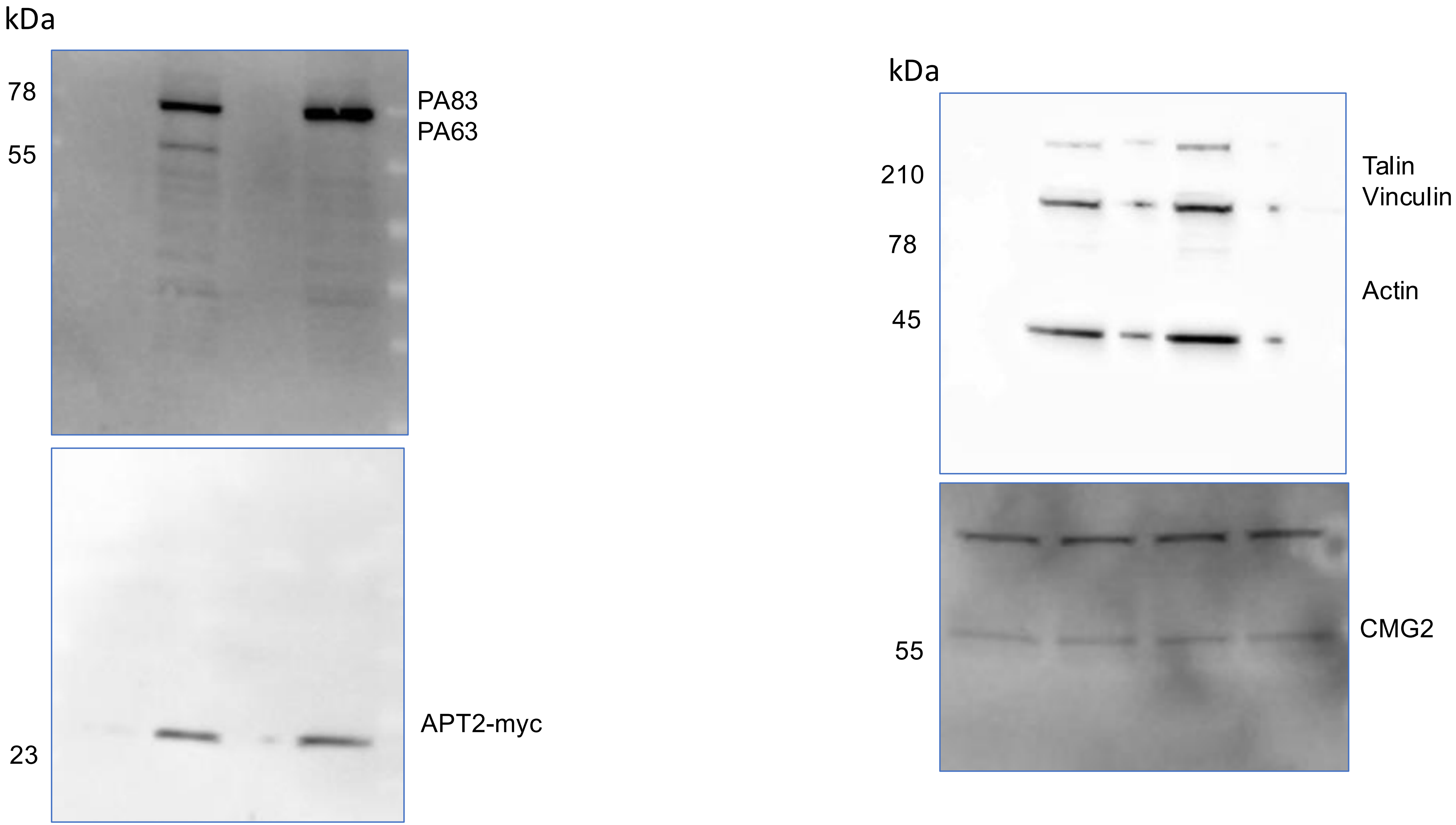
kDa
78
55
PA83
PA63
23
APT2-myc
kDa
210
78
45
Talin
Vinculin
Actin
55
CMG2

Figure 6e

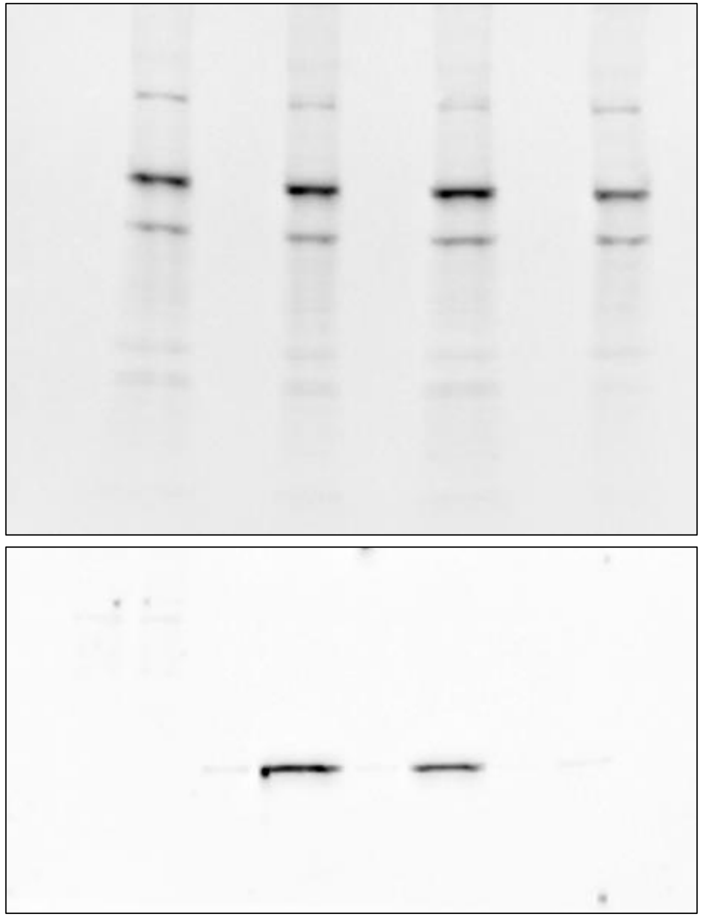

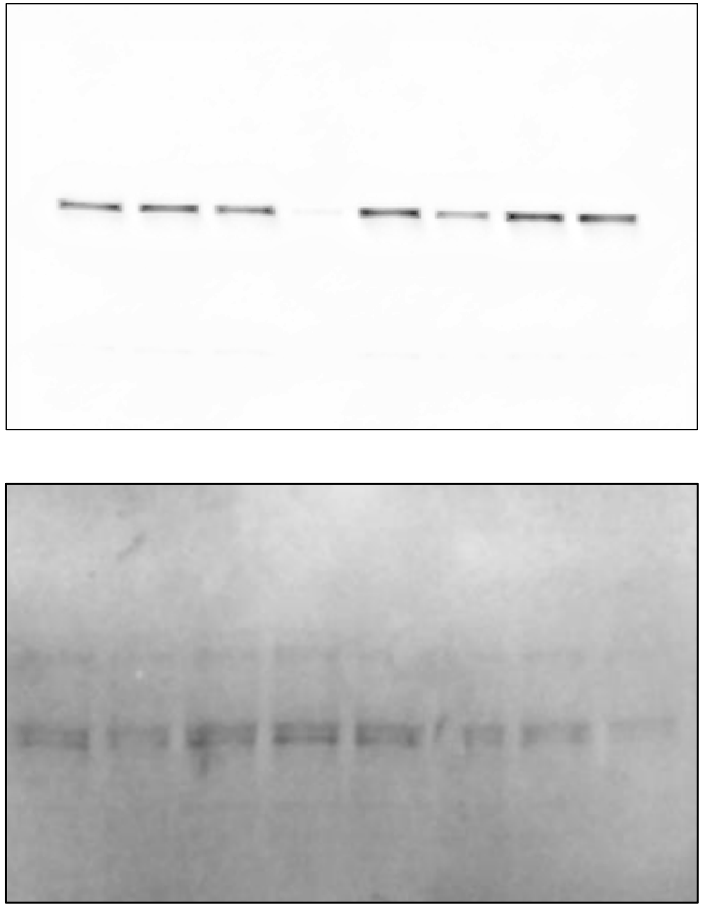

Extended Figure 6b

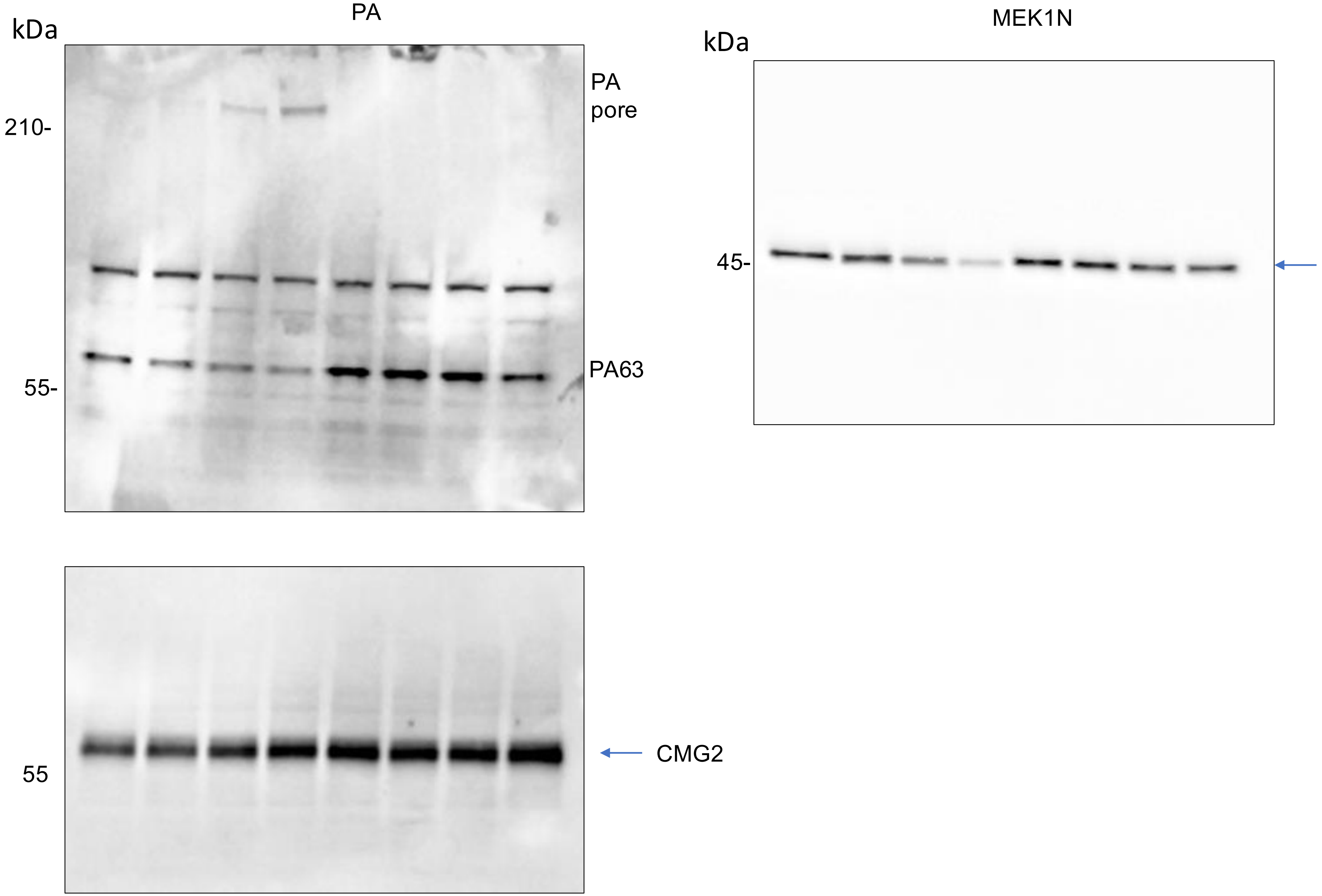

Extended Figure 6c

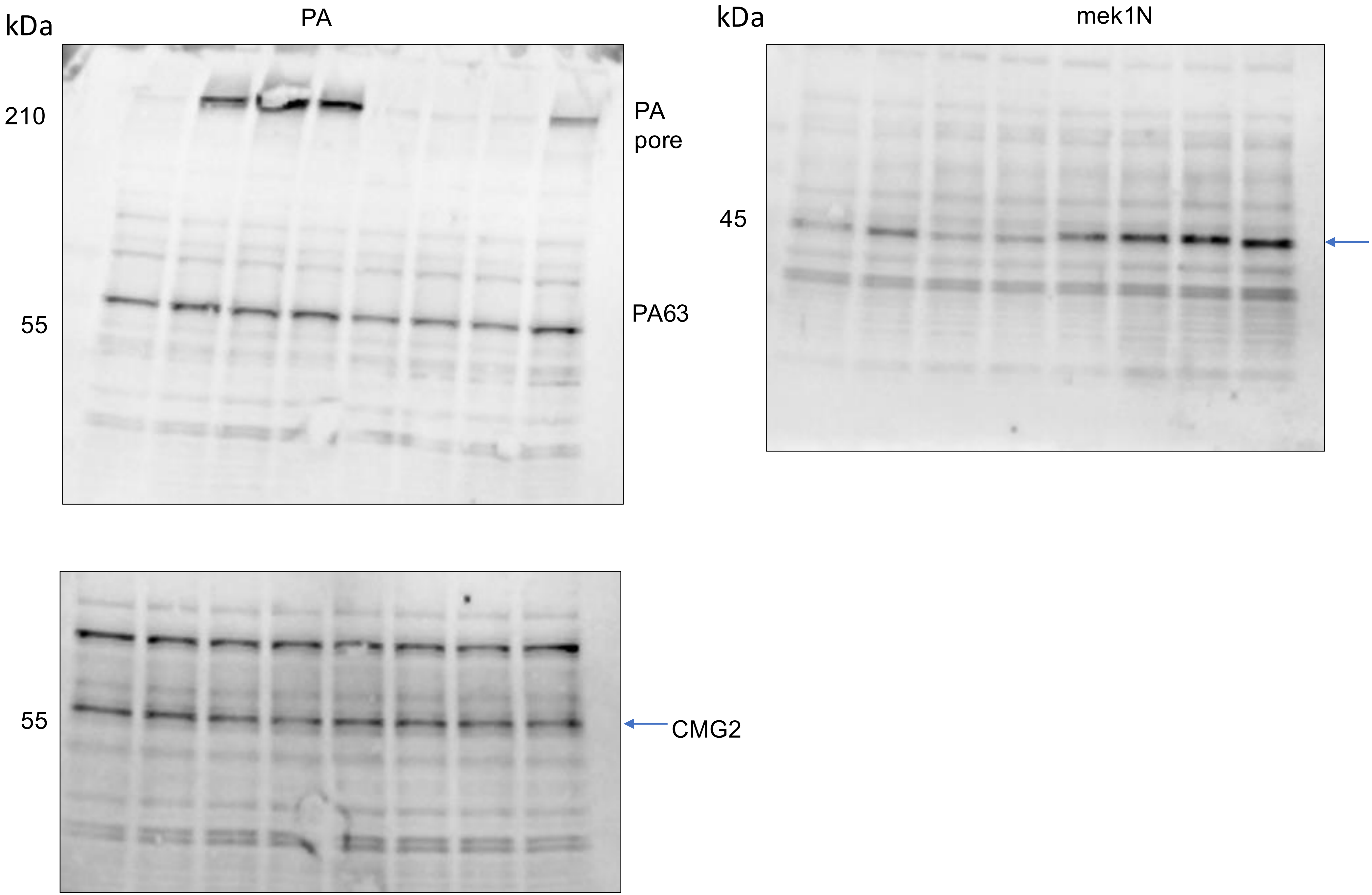

Extended Figure 6d

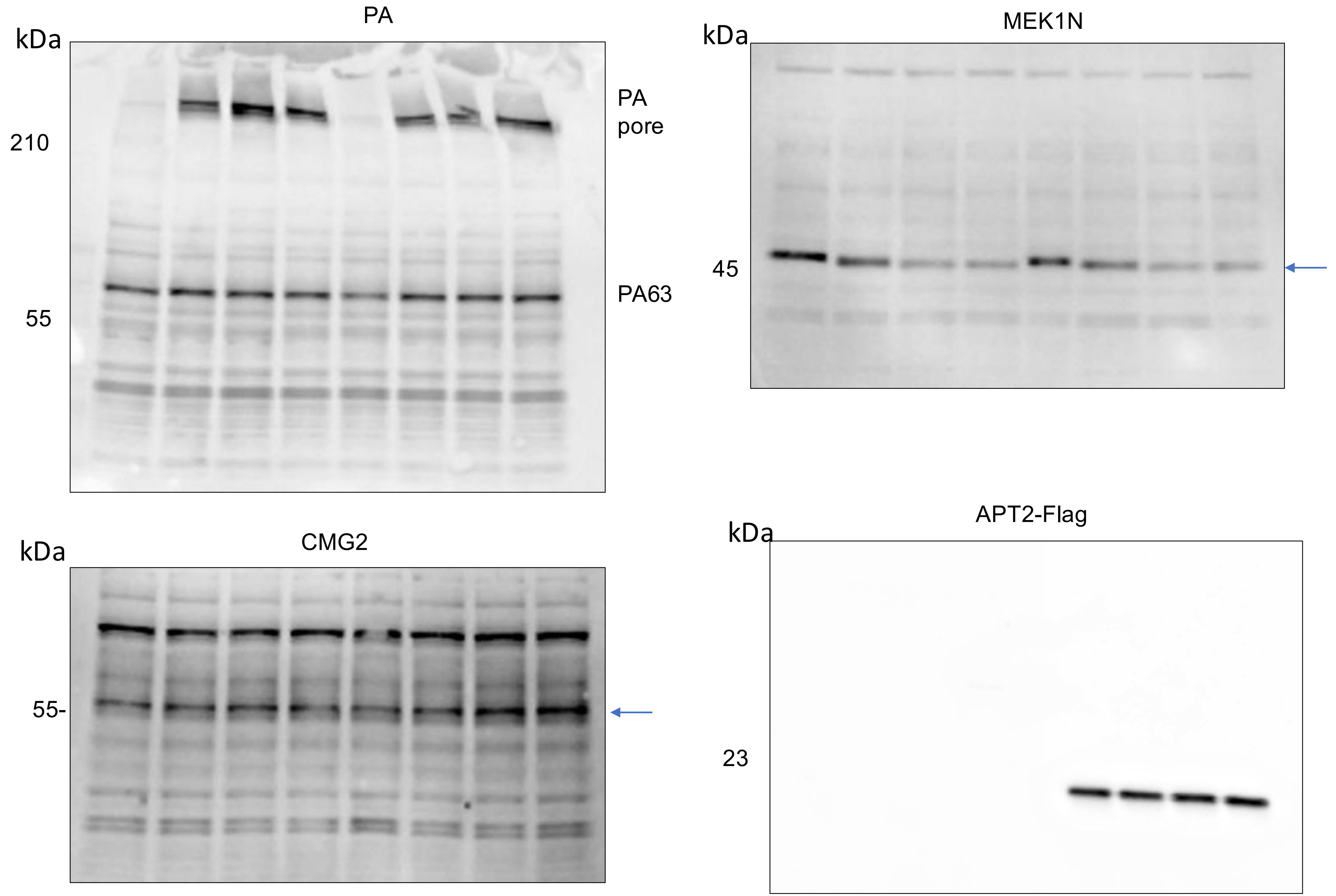

Figure 7c

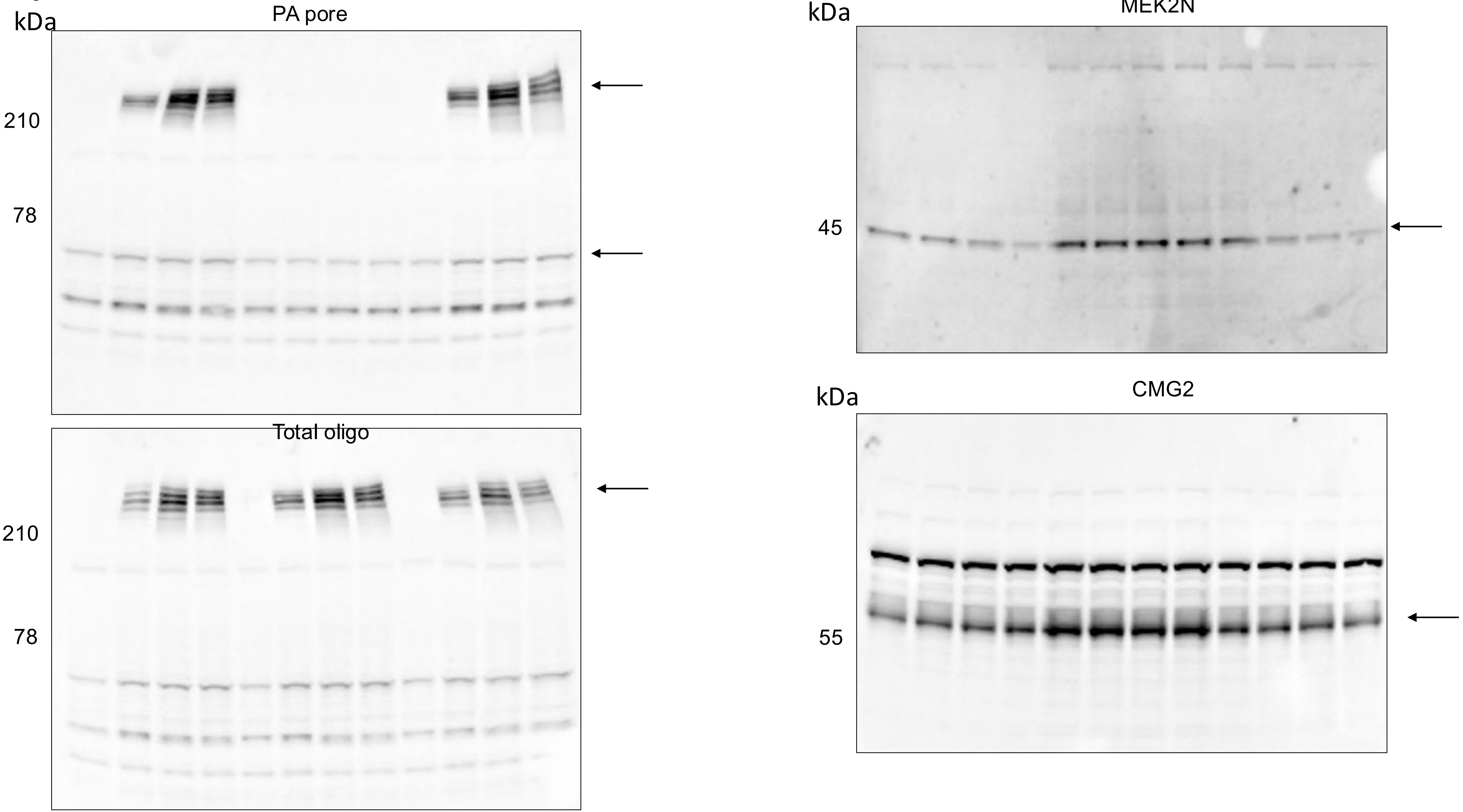

Figure 7f

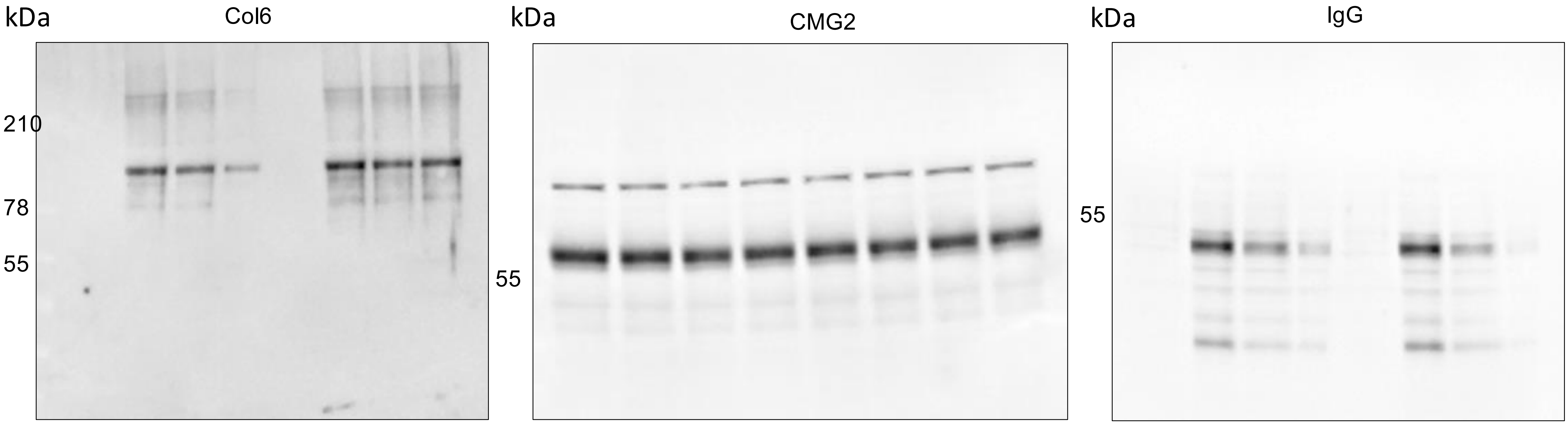

Supplement: Supplementary file 3 [file 41467_2026_75028_MOESM1_ESM.pdf]
