# Supplementary material for: Dynamic S-acylation controls CMG2 maturation extracellular matrix regulation and anthrax toxin susceptibility in vivo
Source: Nat Commun. 2026 Jul 6;17:8364. doi: 10.1038/s41467-026-75028-2 (PMC13473502; doi:10.1038/s41467-026-75028-2)
Supplement: Supplementary file 4 [file 41467_2026_75028_MOESM2_ESM.pdf]

Title: Supplementary Movie 1
Description: Red blood cell circulation throughout the vasculature in a control embryo injected with PBS. Scale bar represents 100µm.

Title: Supplementary Movie 2
Description: Red blood cell circulation is completely blocked throughout the embryonic vasculature upon Lethal toxin injection. Scale bar represents 100µm.

Title: Supplementary Movie 3
Description: Co-injection of Palmostatin B partially restores the circulation defects in the ISVs, aorta and caudal vein caused by Lethal Toxin intoxication. Scale bar represents 100µm.

Title: Supplementary Movie 4
Description: Co-injection of ML349 partially restores the circulation defects in the ISVs, aorta and caudal vein caused by Lethal Toxin intoxication. Scale bar represents 100µm.

Title: Supplementary Movie 5
Description: Red blood cell circulation throughout the vasculature in control embryos injected with PBS.

Title: Supplementary Movie 6
Description: Red blood cell circulation is dramatically impaired in the embryonic vasculature upon Lethal toxin injection.

Title: Supplementary Movie 7
Description: Co-injection of Palmostatin B significantly restores the circulation defects caused by Lethal Toxin in the embryonic vasculature.

Title: Supplementary Movie 8
Description: Co-injection of ML349 significantly restores the circulation defects caused by Lethal Toxin in the embryonic vasculature.
